# Composite effects of gene determinants on the translation speed and density of ribosomes

**DOI:** 10.1186/gb-2011-12-11-r110

**Published:** 2011-11-03

**Authors:** Tamir Tuller, Isana Veksler-Lublinsky, Nir Gazit, Martin Kupiec, Eytan Ruppin, Michal Ziv-Ukelson

**Affiliations:** 1 Department of Biomedical Engineering, Faculty of Engineering, Tel Aviv University, Ramat Aviv 69978, Israel; 2 Department of Computer Science, Ben Gurion University of the Negev, Beer-Sheva 84105, Israel; 3 Blavatnik School of Computer Science, Tel Aviv University, Ramat Aviv 69978, Israel; 4 Department of Molecular Microbiology and Biotechnology Tel Aviv University, Ramat Aviv 69978, Israel; 5 School of Medicine, Tel Aviv University, Ramat Aviv 69978, Israel

## Abstract

**Background:**

Translation is a central process of life, and its regulation is crucial for cell growth. In this article, focusing on two model organisms, *Escherichia coli *and *Saccharomyces cerevisiae*, we study how three major local features of a gene's coding sequence (its adaptation to the tRNA pool, its amino acid charge, and its mRNA folding energy) affect its translation elongation.

**Results:**

We find that each of these three different features has a non-negligible distinct correlation with the speed of translation elongation. In addition, each of these features might contribute independently to slowing down ribosomal speed at the beginning of genes, which was suggested in previous studies to improve ribosomal allocation and the cost of translation, and to decrease ribosomal jamming. Remarkably, a model of ribosomal translation based on these three basic features highly correlated with the genomic profile of ribosomal density. The robustness to transcription errors in terms of the values of these features is higher at the beginnings of genes, suggesting that this region is important for translation.

**Conclusions:**

The reported results support the conjecture that translation elongation speed is affected by the three coding sequence determinants mentioned above, and not only by adaptation to the tRNA pool; thus, evolution shapes all these determinants along the coding sequences and across genes to improve the organism's translation efficiency.

## Background

Gene translation is a central biological process in all living organisms by which an mRNA sequence is decoded by the ribosome to synthesize a specific protein. During the elongation stage of this process, each codon is iteratively translated by the ribosome to an amino acid. Translation elongation is known to be conserved in all living organisms (Bacteria, Archaea, and eukaryotes [1]); thus, understanding this process and the determinants related to it have important ramifications for human health [2-4], biotechnology [5-10], and evolution [4,8,11].

Indeed, gene translation has been the topic of an increasing number of studies in recent years (see, for example, [5,7,8,12-20]). Specifically, it was recently discovered that the efficiency of translation can be controlled by the codon order in the coding sequence [8,17]. This is partially achieved by a 'ramp' at the beginning of the coding sequences composed of less efficient codons. This ramp slows down ribosomal speed, and thus improves their allocation and minimizes the number of collisions between them. In addition, it was shown that there is global selection for weak mRNA folding at the beginning of the coding sequence to improve the binding of ribosomes [7,8,14,16,17,21,22]. Furthermore, recent, small-scale studies also suggested that positively charged amino acids slow down ribosomes as the electrostatic potential inside the exit tunnel is negative [23,24]. Finally, based on large scale measurements of ribosome densities [13,15] it has been demonstrated that the density (and thus the speed [8]) of ribosomes varies within a gene and across genes.

We have previously shown that the speed and allocation of ribosomes in genes is affected by the distribution of the adaptation of codons along them to the tRNA pool of the organism [8]. The goal of this paper is to study how the different features of coding sequences interact to affect the speed of ribosomal movement and allocation. Our results may suggest that selection forces act to slow down the speed of ribosomes at the beginning of genes. This is likely to improve allocation of ribosomes and prevent traffic jams and collisions between ribosomes [8]. This is achieved not only via selection for slower codons in these regions but also by increasing mRNA folding strength and the frequency of amino acids with a positive charge in these regions.

Furthermore, we show that there is selection to increase the robustness to transcriptional errors at the beginnings of ORFs, which might change these three features, pointing to the specific importance of this region in translation regulation.

## Results

### Computing the genomic profiles of codon bias, charge and folding energy

We defined three genomic profiles of coding sequence determinants: (1) a profile of codon bias co-adaptation to the tRNA pool; (2) the amino acid charge pattern; and (3) the profile of local mRNA folding energy.

The profile of co-adaptation of the codon bias to the tRNA pool is based on the tRNA adaptation index (tAI) measure [25] and represents the co-adaptation between the coding sequences and the tRNA pool of the organism. The tAI is superior to other measures of codon bias as it yields higher correlations with protein abundance than the alternative measures and is a more direct measure of adaptation to the tRNA pool. It is based on the coding sequences and the genomic copy numbers of tRNA molecules (which were shown to be highly correlated with their cellular tRNA levels; more details are provided in Materials and methods and Note S1 in Additional file 1).

The tAI of a codon is higher if it is recognized by more abundant tRNA molecules; thus, on average, the recognition time of the codon by the right tRNA is shorter [8]. The *i*-th entry in the genomic codon bias profile is computed as the mean tAI of the *i*-th codons across genes (of substantial length [8]; Materials and methods).

The charge profile represents the position-specific average charge of the amino acid chains across genes. The *i*-th entry in the charge profile is the mean charge of the *i*-th amino acid across genes where 1 represents a positive charge (amino acids Arg, His and Lys), -1 a negative charge (amino acids Asp and Glu), and 0 no charge (all other amino acids) (Materials and methods). The exit channel follows the peptidyl transferase center, where the catalytic reaction of the ribosome takes place; the polyleptide thus must traverse two negatively charged regions to exit [24,26]. Thus, charged amino acids that are encoded in the codons preceding (upstream) the translated codon should have electrostatical interactions with the ribosome.

The folding energy profile was computed as follows. First, we computed for each gene a profile of local folding energies (Materials and methods); the folding energy corresponding to the *i*-th codon is the folding energy of a 40-nucleotide window that begins with this codon. Folding energies corresponding to nucleotides before the start codon (that is, at the 5' UTRs) were defined in a similar way. In the next stage, we computed the mean folding energy for each entry (position) in a similar way to the tAI and charge profiles described above (Materials and methods). Stronger folding corresponds to lower (more negative) folding energy. It was shown before [14,27] that the correlation between the folding energy and protein abundance is very weak in endogenous genes. However, in this study we focus on the effect of folding energy on the density of ribosomes and their allocation.

In addition, due to a novel approach for measuring ribosomal density at single nucleotide resolution, which was performed for numerous *Saccharomyces cerevisiae *genes [15], it is possible to plot, in a similar manner, a genomic ribosomal density profile. The *i*-th entry in this profile is the mean ribosomal density of the *i*-th codon across genes (of substantial length).

**Figure 1:**
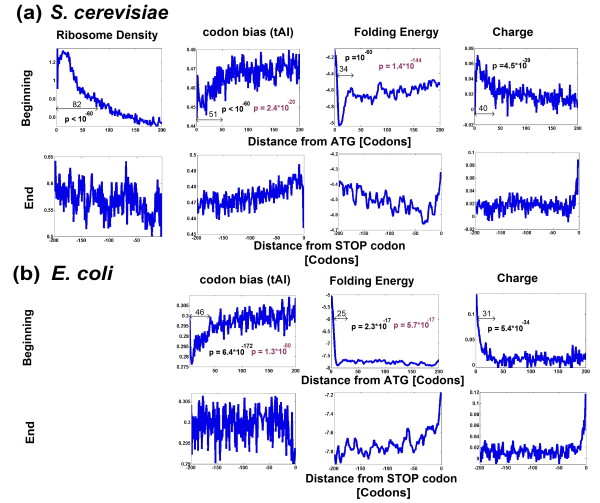
**Mean genomic profiles of three features of the coding sequences**. The mean genomic profiles of ribosome density, tAI, folding energy, and amino acid charge in *S. cerevisiae *and *E. coli *when aligning all the genes to their beginning (upper panels; Materials and methods) or end (lower panels; Materials and methods). In the case of the beginning profile, each panel also includes the region of the ramp (Materials and methods), a *P*-value corresponding to a comparison of the ramp to the rest of the profile (black; Materials and methods), and a *P*-value corresponding to a control for amino acid content (brown; Materials and methods).

The three genomic profiles of *S. cerevisiae *and *Escherichia coli*, and the genomic profile of ribosomal density in *S. cerevisiae *are shown in Figure 1. As we mentioned earlier, it was reported in previous studies that the co-adaptation profile of codons to the tRNA pool correlates with the ribosomal density profile, and that this profile has a 'ramp' of slower translation speed at the beginning of coding sequences [8]. Figure 1 demonstrates that the actual ramp has three dimensions.

However, there are two additional dimensions:

First, the genomic profile of folding energy contains a region of stronger folding (after an initial weak folding region that promotes ribosomal binding; see Figure S1 in Additional file 2 for the median profile). Similar results were obtained when analyzing measurements of mRNA folding [28]: at the beginning of the coding sequence there are usually nucleotides that are not involved in base-pairing (that is, weak mRNA folding; *P *= 8.9 × 10^-69^) as opposed to downstream nucleotides where the frequency of nucleotides involved in base-pairing increases (that is, strong folding; *P *= 5 × 10^-74^; Materials and methods; Figure S2 in Additional file 2). Second, genes tend to have more positively charged amino acids at their 5' end, which should also contribute to the deceleration of ribosomes. The length of the slower region in each of the three dimensions of the ramp is between 30 and 50 codons, similar to the length of the ribosome's exit channel [17] (the lengths of the ramps and the corresponding *P*-values are given in Figure 1; see Materials and methods for explanations about how these lengths were computed). As can be seen in Figure S3 in Additional file 2 the genomic charge profile is a superposition of the genomic profiles of the individual amino acid frequencies (that is, it is not a result of one specific amino acid).

All three genomic profiles were less coherent at the end of the sequences (lower rows of Figure 1a, b; the ribosomal profile is relatively flat; the tAI profile has an increased efficiency at the end of *S. cerevisiae *genes, but no trend emerges in the case of *E. coli*; the charge profile contributes to reduced speed at the end, and the folding profile contributes to elevated speed due to weak folding). Thus, it seems that the selection forces acting on the 3' UTR ends of the coding sequence are mainly related to amino acid bias and less to translation (as was suggested for part of the features in [8,14]; see also Note S2 in Additional file 1).

Taken together, these results suggest that the speed of ribosome movement and the efficiency of translation elongation result from a superposition of various features of the coding sequence. Thus, the regulation of translation elongation (for example, the 'ramp' at the beginning of genes) has more degrees of freedom than previously reported.

In the next sections we further examine this idea, demonstrating that these ramps are more striking for highly expressed genes, that they are more robust to transcription errors, and that each of the ramps makes a distinct contribution to the ribosomal density.

### The three dimensions of the 'ramp' are accentuated for highly expressed genes and for genes with higher ribosomal density

If the 'ramp's three dimensions are selected for in such a manner as to improve the allocation of ribosomes and prevent ribosome collisions, we expect a more prominent ramp for genes with higher mRNA levels and ribosomal densities, as such genes potentially consume more ribosomes (as was suggested in [8]). The ramp has additional potential advantages (Note S3 in Additional file 1). Thus, we expect to see it also in genes with lower ribosomal density [8].

The ramp's length is the slower region at the beginning of the genomic profile and it is measured relatively to the entire profile (Materials and methods); thus, the ramp region of highly expressed genes can in actuality be more efficient than that of lowly expressed genes, despite the fact that absolute translation rates for lowly expressed genes are lower.

**Figure 2:**
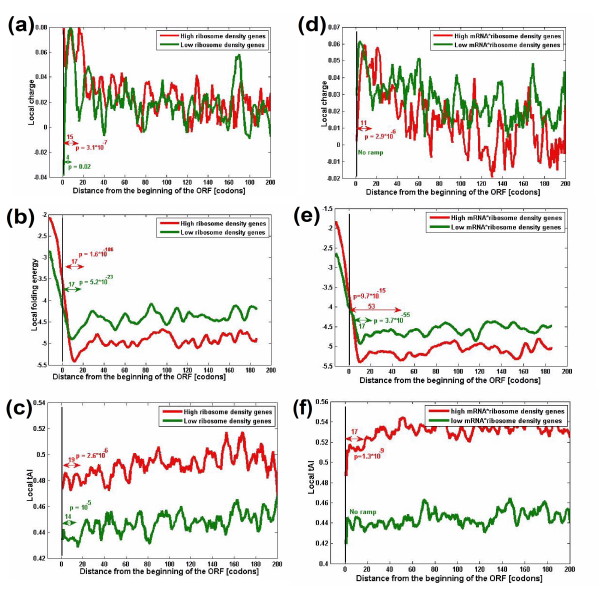
**Profiles of charge, folding energy, and co-adaptation between codon bias and the tRNA pool for genes with high and low ribosomal density**. **(a-c) **Profiles of charge (a), folding energy (b) and co-adaptation between codon bias and the tRNA pool (c) for genes with high ribosomal density (red; top 10%) and genes with low ribosomal density (green; bottom 10%) in *S. cerevisiae*. **(d-f) **Profiles of charge (d), folding energy (e) and co-adaptation between codon bias and the tRNA pool (f) for genes with high (mRNA levels) × (Ribosomal density) (red; top 10%) and genes with low (mRNA levels) × (ribosomal density) (green; bottom 10%) in *S. cerevisiae*. The ramps and corresponding *P*-values are shown in the figure. Similar graphs were obtained for *E. coli *and *C. elegans *(see graphs and corresponding *P*-values in Figures S4 and S5 in Additional file 2).

Figure 2a-c depicts the mean genomic profiles of charge, folding energy and translation efficiency for genes with the highest ribosomal density (the top 10%) versus genes with the lowest ribosomal density (lowest 10%). Indeed, the three dimensions of the ramp are more prominent (relative to the rest of the profile) for the group of genes with the highest values for the product ribosomal density (the charge, folding energy, and tAI ramp lengths are 15, 17, and 19, respectively, for the group with the uppermost values versus 4, 17, and 14, respectively, for the group with the lowest values). The gap between these two groups increases when considering the group of genes with the uppermost values for the product of (mRNA levels) × (Ribosomal density) versus the group of genes with the lowest values for this product; this value represents the actual number of ribosomes 'consumed' by the gene, and the charge, folding energy, and tAI ramp lengths are 11, 53, and 17, respectively, for the group with the uppermost values versus 0, 17, and 0, respectively, for the group with the lowest values (see Figure S2b, c in Additional file 2 for similar results based on folding energy measurements). Similar results were also obtained for other organisms whose mRNA levels are available (*E. coli *and *Caenorhabditis elegans*; Figures S4 and S5 in Additional file 2) or when we analyze measurements of mRNA folding [28] (Figure S2 in Additional file 2; Materials and methods). These results support the conclusions reported above. Specifically, the case of the folding energy profile is more complex as highly expressed genes and genes with higher ribosomal density should have stronger selection for weak folding at the first few codons to promote ribosomal binding and increase the rate of translation initiation [14,21,29]. However, as can be seen in Figure 2, the preceding codons have stronger mRNA folding in the case of highly expressed genes and genes with higher ribosomal density.

We further verified that the three observed profiles are not due to a small group of genes with a specific function(s) that may skew the results (for example, membrane proteins or heat shock proteins; see results in Figures S6 and S7 in Additional file 2; Additional files 3 and 4).

### The three genomic profiles exhibit stronger robustness to transcription errors at the beginning of genes

The error rate in the process of gene transcription is estimated to be 1 in every 10^4 ^nucleotides [30]. Thus, on average, one in every 67 windows with a length of 50 codons will have a transcriptional error. Considering the fact that there are thousands of copies of mRNA molecules in the cell (for example, the number of mRNA molecules in *S. cerevisiae *is around 60,000 [31], and in *E. coli *it is around 1,380 [32]) and that genes are transcribed and translated continuously, together this may amount to a non-negligible error probability (in terms of its effects on an organism's fitness).

**Figure 3:**
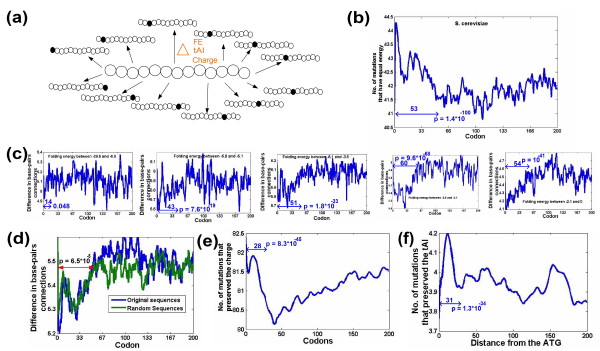
**Genomic profiles of robustness to transcription error demonstrate that there is an increased selection for robustness at the beginning of genes in *S. cerevisiae***. **(a) **An illustration of the robustness computation: for each sliding window (length 13 codons) and in every coding sequence we computed the mean distance (in terms of folding energy (FE), tAI, and charge) from all its single nucleotide point mutations. In the next stage, the genomic profiles of robustness were plotted and analyzed (see more details in the Materials and methods). **(b) **The genomic profiles of robustness to transcription error (mean number of mutations that do not change the mRNA folding). The number of transcription errors (point mutations) that change the folding energy is lower at the beginning of genes (*P *= 1.4 × 10^-100^, Kolmogorov-Smirnov (KS) test). **(c) **The profiles of robustness to transcription error for five bins of equal size corresponding to the folding energy of the windows (Materials and methods; the boundaries of each bin are reported in the figures). The increased robustness at the beginning of genes remains significant even when controlling for local folding energy of the mRNA sequences. **(d) **The robustness to transcriptional errors in terms of folding energy is stronger than in randomized sequences (that maintain the codon bias and amino acid content of the original sequences; Materials and methods) at the beginning of genes (*P *= 6.5 × 10^-5^, KS test). **(e, f) **Profile of the robustness to transcriptional errors in terms of charge (e) and tAI (f). There is increased robustness at the beginning of genes in terms of the charge (e) as well as in terms of tAI (f). Ramp length and corresponding *P*-values are reported in the figures.

To study the robustness to transcription error, we used three measures of the robustness of mRNA sequence to transcription errors in terms of its folding structure and energy. The first measure is the mean change (over all point mutations) in mRNA folding energy; the second measure is the number of errors causing modification of mRNA folding; and the third is the mean number of nucleotide-nucleotide connections that are present/absent in the two-dimensional folding structure of the original mRNA sequence but absent/present in the two-dimensional structure of the mutated one [33] (Figure 3A; Materials and methods). These measures were computed for all sliding windows of 40 nucleotides (close to the footprint of the ribosome on the mRNA sequence; Materials and methods) in all *S. cerevisiae *and *E. coli *genes.

**Figure 4:**
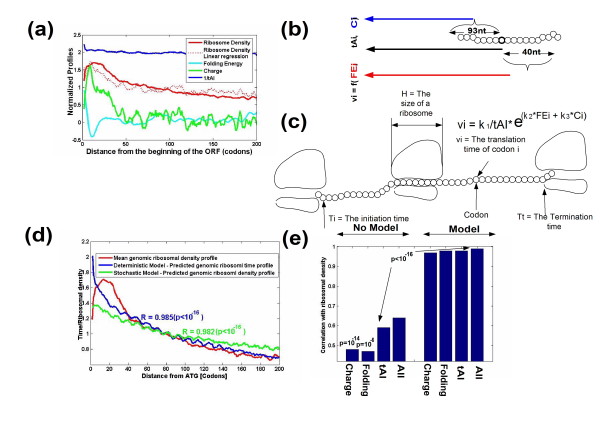
**Predictions and modeling of the ribosomal density profile based on the features of the coding sequence**. **(a) **Genomic profiles of tAI, folding, charge, a linear regressor based on all these variables, and ribosomal density. In the case of the linear regressor, the predicted ribosomal density is plotted as a function of the distance from the beginning of the ORF (x-axis). The linear regressor is a better predictor of the ribosomal density profile than each of these variables separately (dot plots in Figure S32 in Additional file 2). **(b, c) **Modeling of ribosomal velocity and density. (b) The velocity of translating the *i*-th codon is a function of the co-adaptation of the codon to the tRNA pool of the organism, the tAI, the folding energy (FE) after the codon (40 nucleotides), and the charge of the amino acids before the codon (31 amino acids; Materials and methods). (c) To compute the actual velocity we should also consider the initiation and termination times and the fact that a ribosome may be delayed by the ribosome in front of it due to 'traffic jams' (Materials and methods). **(d) **The profile of ribosomal density (red) versus the predicted profile of translation times based on a deterministic model (green; see (b, c)) and the predicted profile based on a stochastic model (blue; Materials and methods). **(e) **Correlations of various predictors of ribosomal density with the actual ribosomal density. The predictor that is based on the three variables and the model of ribosomal movement achieved the highest correlation.

We found a significant signal for increased robustness to transcription errors in terms of the folding energy at the beginning of genes (*P *= 1.4 × 10^-100^; Figure 3B; Materials and methods). The signal remains significant when controlling for the folding energy of the mRNA sequences (that is, to rule out the possibility that the robustness is a result of the more extreme folding in this region as mentioned in the previous sections; all *P*-values < 0.05; the most significant *P*-value = 9.6 × 10^-68^; Figure 3C; Materials and methods) and when comparing the profile to that of randomized sequences with identical amino acids (and maintaining the codon bias of the organisms; that is, controlling for amino acid bias; *P *= 6.5 × 10^-5^; Figure 4D; Materials and methods). Thus, these results suggest that at the beginning of genes there is selection for increased robustness to transcription errors in terms of changes in mRNA folding. Similar results were obtained for *E. coli *(see all the results, controls, and corresponding *P*-values in Figures S11 to S19 in Additional file 2).

Next, we performed a similar analysis with respect to the tAI and the charge for windows of 13 codons (the footprint of the ribosome on the mRNA [15,34], small variations in the window size did not alter the conclusion; Materials and methods). Our analysis demonstrates that also in these cases, the beginnings of genes tend to be more robust relative to other parts of the coding sequence (*P *= 8.3 × 10^-45 ^for charge and *P *= 1.3 × 10^-34 ^for the tAI; Figure 3e, f; see Figures S20 to S25 in Additional file 2 for various controls related to these genomic profiles of robustness, as was performed for the folding energy robustness). In the case of the charge robustness profile, the profile also includes a decrease in the robustness in the second half of the 'ramp' region, followed by a gradual return that is close to the baseline (but still lower). The unique shape of this profile suggests that it was influenced by additional determinants that are not necessarily related to the 'ramp'. Similar results were obtained for *E. coli *(see plots and *P*-values in Figures S20 to S25 in Additional file 2).

It is possible that different transcription errors have different occurrence probabilities. To the best of our knowledge, however, there are no measurements/estimations of these errors. Nevertheless, we show that the robustness profiles obtained under the assumption that the probability of a transition error (a change of a purine by a purine and of a pyrimidine by a pyrimidine) is higher than the probability of a transversion error (a change to a different base type) remain very similar (see Figures S26 to S31 in Additional file 2 and the corresponding *P*-values; see details in Materials and methods). In addition, in the case of the charge robustness profile, both transcription and translation errors are relevant. Thus, we also show that, when considering the fact that translation errors are very rare in the second nucleotide of a codon [35], the charge robustness profile remains very similar (Figures S30 and S31 in Additional file 2; see details in Materials and methods).

### The three genomic profiles explain the ribosomal density profile

#### Each of the three coding sequence profiles has significant partial correlation with the ribosomal density profile

Under the assumption of a constant flux of ribosomes, the density of ribosomes is higher for lower ribosomal velocity and it is proportional to 1/(Ribosomal velocity) [8]. In this section, we aim to verify that the three profiles of coding sequence features (previously reported) contribute to the genomic ribosomal density profile [8,15] and thus to the ribosomal speed profile. If there is correlation between the ribosomal speed or density and features of the coding sequence, it should appear along the entire sequence and not only at the beginning. As longer sequences should have larger statistical power (but the length should be shorter than most of the genes), we decided to check the three profiles along 200 codons.

First, we found that the three genomic profiles of the coding sequence correlate significantly with the profile of ribosomal density. The correlation of the tAI profile and the ribosomal density profile is -0.59 (*P *< 10^-16^; for the first 200 codons; as reported in [8]); the correlation of the folding energy profile and the ribosomal density profile is -0.4743 (*P *= 1.05 × 10^-6^; for codons 5 to 100; as reported in [14]); the correlation of the charge profile and the ribosomal density profile is 0.48 (*P *< 5.25 × 10^-13^; for the first 200 codons; dot plots in Figure S32 in Additional file 2).

Second, we verified that these correlations are maintained even if we control for the other two variables (Materials and methods; Note S4 in Additional file 1). Indeed, all partial correlations were significant: the partial correlation of the charge and the ribosomal density profile given the other two variables, *R*(*Charge*, *Ribosomal density | tAI*, *Folding*), is 0.314 (*P *= 6.5 × 10^-6^; empirical *P *< 0.01); the partial correlation of the tAI and the ribosomal density profile given the other two variables, *R*(*tAI*, *Ribosomal density | Charge*, *Folding*), is -0.47 (*P *= 3.35 × 10^-12^; empirical *P *< 0.01); the partial correlation of the local folding and the ribosomal density profile given the other two variables, *R*(*Folding*, *Ribosomal density | Charge*, *tAI*), is -0.224 (*P *= 0.0015; empirical *P *< 0.01).

In addition, when we inferred a linear regressor (Materials and methods) based on the three features of the coding sequence we obtained an improved correlation with ribosomal density compared to the correlation with each of the features separately, resulting in a plot that significantly resembles the ribosomal density graph (Spearman correlation 0.87, *P *< 10^-16^; Figure 4a; Figure S32d in Additional file 2; when we did not consider the first 50 codons (the region of the ramp) the correlation was only 0.33, *P *= 4 × 10^-5^). The formula of the regressor was: (*1*/*tAI*) × 3.18 *+ Folding energy *× (-0.177) *+ Charge *× 5 - 3.034. In addition the *P*-values (confidence intervals) of the three features were significant (folding *P *= 0.01; charge and tAI *P*-values < 0.005), suggesting that all three have a significant contribution to the regressor.

The results remained robust when we performed leave-one-out iterations, where in each iteration the regressor was inferred based on 50% of the sequences and was applied on the remaining sequences (*P *< 0.01; Materials and methods).

As a whole, the results reported in this section demonstrate that each of the features of the coding sequence makes a distinct contribution to the translation rate and density of ribosomes. In addition, the results reported in this section suggest that the tAI makes the most substantial contribution, whilst the folding energy makes the smallest, to the ribosomal density profile.

#### An integrated model of ribosomal density and translation rate

We investigated the possibility of improving the correlation with the genomic profile of ribosomal density by employing a model based on: 1) the three features of the coding sequence; and 2) ribosomal size and the interactions between them [8,34].

A depiction of the model is shown in Figure 4b, c: the nominal velocity of each codon is a superposition of its tAI, the charge of the amino acid before the codon and the folding energy before and after the codon (see the exact details in Materials and methods). In addition, a ribosome translating slower codons may block the advancement of ribosomes moving behind it (Materials and methods). Assuming constant ribosomal flux and no ribosomal abortion, the length of time a ribosome translates each codon should be proportional to the ribosomal density of the codon [8]. Indeed, when we correlated the predictions of this model (the mean genomic translation time of codons) with the genomic profile of ribosomal density, the correlation was near maximal (r = 0.982; *P *< 10^-16^; Figure 4d, e) and was significantly better when we considered all three genomic features rather than a subset of them (*P *< 0.05; Materials and methods); a similar correlation (r = 0.98; *P *< 10^-16^) was obtained when we performed a cross-validation (Materials and methods). We found that small changes in the model (regarding the subsequences near the codon that affect its translation; Materials and methods) have minor effect on the results of the model (all correlations between 0.984 and 0.985) but the slope of the current model (Figure 4b, c) better resembles the slope of the measured ribosomal density profile.

In addition, when we used a stochastic model of gene translation [36] (Materials and methods) we were able to get a slope of the predicted genomic profile of ribosomal density that better resembles the slope measured from the ribosomal density profile (Figure 4d). However, the correlation remained as in the deterministic case (r = 0.985; *P *< 10^-16^).

### Ribosomal densities versus the three coding sequence features: a site-by-site comparison

In this section, we establish the existence of a relationship between coding sequence features and ribosomal density in each individual gene. However, we expect that such correlations will be much lower than those with the genomic profiles, due to the noisiness of ribosomal density measurements (Materials and methods). In addition, the measures used for estimating the adaptation to the tRNA pool and the effect of charge/folding are only approximations of the real measures.

**Figure 5:**
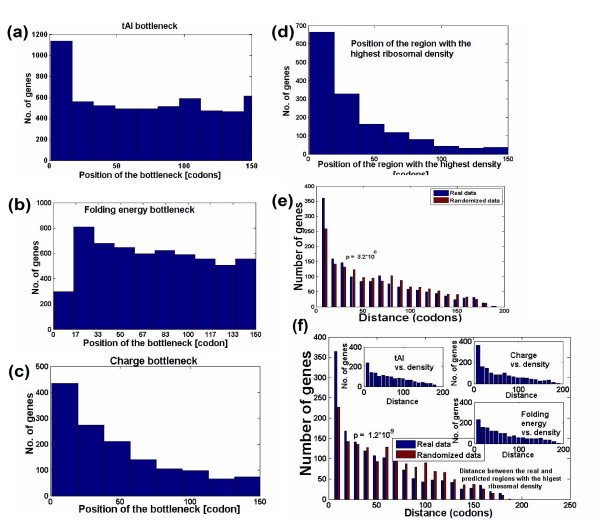
**Relationship between ribosomal density, local tAI, folding, and charge in single genes of *S. cerevisiae***. **(a) **Histogram of the positions of the tAI bottleneck (the region with the slowest tAI). **(b) **Histogram of the positions of the folding energy bottleneck (the region with the strongest folding energy). **(c) **Histogram of the positions of the charge bottleneck (the region with the highest positive charge). **(d) **Histogram of the positions of the region with the highest ribosomal density. **(e) **Histogram of the distance between the composite bottleneck (based on the regressor that weighs the tAI, charge, and folding energy; Materials and methods) and the region with the highest ribosomal density positions. **(f) **Histogram of the distance between the composite bottleneck (based on the ribosome movement model; Materials and methods) and the region with the highest ribosomal density positions. Similar results were observed when we performed cross-validations.

First, we computed the 'bottleneck' for each gene, that is, the slowest region (10 codons; small changes in the window gave similar results) in terms of adaptation to the tRNA pool, charge, and folding energy (considering the first 200 codons of the gene). As expected (Figure 5a-d), most genes exhibit these three 'bottlenecks' at the beginning of the ORF (first 40 codons), rather than in any other region. The result demonstrates that the three dimensions of the 'ramp' previously reported can be observed at the single gene level, and that the genomic profiles are not the result of a minute set of genes with a large impact.

We then searched for the window with the highest ribosomal density in each individual gene and compared its position to the position with the slowest translation rate, based on the three genomic features. Figure 5e, f shows that, in addition, this window tends to be the one predicted based on the combination (that is, the linear regressor (Figure 5e) or the model (Figure 5f)) of the three genomic features (*P *= 3.2 × 10^-6 ^and *P *< 1.2 × 10^-9^, respectively; Materials and methods). Thus, again, this result suggests that the correlation between ribosomal density and the three genomic features can be observed at the single gene level.

## Conclusions

In this study we have rigorously shown that the rate of translation elongation on native genes can be largely determined by knowledge of the three primary features of the coding sequence: the folding energy of the mRNA, its codon bias and the amino acid charge. More precisely, our analysis shows that the translation rate of a ribosome at a certain codon along the coding sequence can be determined by the codons before it (the amino acids that are in its exit channel) and after it (the unfolding of the mRNA structure by mRNA helicases). These features are not distributed uniformly along the coding sequence, probably due to selection for slower ribosomal translation rates (higher ribosomal density) at the beginning of the coding sequences in order to improve ribosomal allocation and decrease ribosomal jamming [8]. Furthermore, we have ascertained that these results remain significant under various controls, in various organisms, and for different sets of genes (Note S5 in Additional file 1 covers additional genomic profiles that relate to translation).

It is important to note that although the predicted genomic ribosomal density profile highly correlated with the measured profile, there is still a gap between the shapes of both profiles (for example, the slope of the measured profile is higher; Figures 4 and 5). This gap may be the result of additional factors that are related to ribosomal translation speed that were not taken into account in this paper; among these factors are the initiation rate, ribosomal abortion, and condition-specific tRNA abundance and mRNA folding. Furthermore, this gap might also be partially related to noise and bias in the ribosomal density measurements (Materials and methods).

One important aspect of gene translation that clearly can be improved in the model presented in this study is the initiation step. In the future we plan to improve our model by taking into account different features of the 5'UTR (for example, mRNA folding energy, lengths of the 5'UTR, the Kozak context of the first ATG of the ORF [37], and the number of times the sequence ATG appears in the 5'UTR) and by modeling ribosomal abortions; we believe that these changes in the model will improve the ribosomal density predictions.

It is important to remember that in this paper we analyze native genes; thus, it is possible that part of the reported effect of the coding features on the ribosomal density profile is not causal. To verify this point, further experimental studies of ribosomal profiles based on variants of the same non-native protein(s) should be performed (as was done in [7,38] for studying determinants of protein abundance).

In addition, we demonstrate that coding sequences have increased robustness to transcription errors at their beginning, in terms of these three features. While robustness to mutations related to DNA mutation in terms of their effect on the properties of the amino acid they encode have been demonstrated before [35], here we suggest a new type of robustness - increased robustness to transcription error in terms of the effect of such mutations on translation at the beginning of the coding sequences. The results reported in this paper and in [35] may suggest that the robustness of the genetic code is partially related to the resilience of the ribosome processing speed.

The results reported in this paper may suggest that mutations/errors at the beginning of the coding sequence that alter the tAI/folding energy/charge usually have a higher influence on the fitness of the organism than mutations/errors occurring in other regions of the coding sequence. As we can not prove causality by analyzing endogenous genes, further experimental analysis is needed to verify if this is indeed the case.

This increased robustness can be related directly to ribosomal allocation, which is more affected by mutations in the ramp, but may also be indirectly related, for example, to an increased effect on misfolding of proteins and the production of toxic proteins (see, for example, [39]). Thus, it is not clear how to evaluate the contribution of ramp robustness to the fitness of an organism.

Thus, the increased robustness to transcription errors in the first 30 to 50 codons may suggest that this is the most critical region of the coding sequence for the regulation of gene translation and ribosomal allocation. A possible explanation of this result is the fact that this region is occupied by relatively more ribosomes (see, for example, Figure 1), that is, it is subject to heavier ribosomal traffic. Thus, it is possible that changes in elongation rates due to transcription errors in coding regions that are occupied by more ribosomes have a larger effect on an organism's fitness.

The results reported in this paper suggest practical ways to optimize heterologous coding sequences in order to express them in a new host, a common biotechnological task (see, for example, [6,7,38]). Since the rate of translation elongation is affected by not just codon bias, one should also consider the effect of the chosen codons on the folding energy (and/or the charge) of the sequences. Specifically, a 'ramp' that slows down ribosomes is helpful to increase the fitness of the host and thus the protein production rate [8]. This ramp should be shaped according to the combined effect of the folding energy, charge and codon bias of the coding sequences.

## Materials and methods

### Various sources of information

#### tRNA copy numbers

The tRNA copy numbers of *S. cerevisiae *were downloaded from [40]; other tRNA copy numbers were downloaded from [41].

#### Coding sequences

The coding sequences of the analyzed organisms were downloaded from the FTP site of the National Center for Biotechnology Information (NCBI).

#### Protein abundance

Protein abundance measurements were downloaded from [42].

#### Gene Ontology associations

The Gene Ontology (GO) associations of *S. cerevisiae *genes are from [43].

#### Gene expression

mRNA levels of *E. coli *were downloaded from [18]; mRNA levels of *S. cerevisiae *were downloaded from [15]; mRNA levels of *C. elegans *come from the Gene Expression Omnibus (GEO) [44] (GDS1786).

#### Lists of ribosomal proteins

The lists of ribosomal proteins were downloaded from [43].

#### Measurements of mRNA folding

Measurements of mRNA folding in *S. cerevisiae *genes are from [28].

#### Ribosomal densities

We used two data sources of ribosomal density in *S. cerevisiae*. The first dataset comes from Arava *et al*. [13] and includes measurements of ribosome number on each mRNA molecule (without information about the per-codon density); they were used to generate Figure 2. To obtain ribosomal densities, we normalized these values by the length of the ORFs.

The second dataset [15] includes measurements of ribosomal density at a single nucleotide resolution. This dataset is noisy at the single gene level (for example, the ribosomal density along a gene may change from a positive number to zero and, again, to a positive number) but when considering large enough sets of genes, it enables a good estimation of the spatial ribosomal density trend.

#### Data generated in this paper

The data that were generated in this study can be downloaded from [45].

### Computing folding

Folding energy was calculated using the Vienna package [46].

### Computing the tRNA adaptation index

We computed the tAI similarly to the way it was computed in the work of dos Reis *et al*. [25]. This measure gauges the availability of tRNAs for each codon along an mRNA. As codon-anti-codon coupling is not unique due to wobble interactions, several anti-codons can recognize the same codon, with different efficiency weights (see dos Reis *et al*. for all the inter-codon-anti-codon relations).

Let *n_i _*be the number of tRNA isoacceptors recognizing codon *i*. Let *tCGNij *be the copy number of the *j*-th tRNA that recognizes the *i*-th codon, and let *S_ij _*be the selective constraint on the efficiency of the codon-anti-codon coupling. We define the absolute adaptiveness, *W*_*i *_, for each codon *i *as:

$$W_{i}=\overset{n_{i}}{\underset{j=1}{\sum }}\left(1-S_{ij}\right)tCGN_{ij}$$

From *W_i _*we obtain *w_i_*, which is the relative adaptiveness value of codon *i*, by normalizing the *W_i _*values (dividing them by the maximum of all 61 *W_i _*values).

The final tAI of a gene *g*, is the following geometric mean:

$$tAIg={\left(\overset{l_{g}}{\underset{k=1}{\prod }}w_{ikg}\right)}^{1∕l_{g}}$$

where *i_kg _*is the codon defined by the *k*-th triplet on gene *g*; and *lg *is the length of the gene (excluding stop codons).

We implemented one alteration compared to the computations of dos Reis *et al*.; we re-inferred the *S_ij _*values (appearing in the equation above) by performing a hill-climbing optimization of the Spearman correlation between protein abundance and translation efficiency in *S. cerevisiae*.

To this end we used the protein abundance measurements mentioned above.

The *S_ij _*values can be organized in a vector (S vector) as described in [25]; each component of this vector is related to one wobble nucleoside-nucleoside pairing: I:U, G:U, G:C, I:C, U:A, I:A, and so on.

### Computing profiles of tRNA adaptation index, folding and charge

The local folding profile of a gene was defined as the vector of the folding values assigned to the sliding windows of length 40 nucleotides, that is:

$$Local\text{\_}FE_{Gene_{i}}=\left(FE_{1},FE_{2},...,FE_{n}\right)$$

where *FE *is the folding energy.

All the genes in the genome were lined up once according to their start codon, and once according to their stop codon. The two profiles of mean folding energy were calculated as:

$$\begin{array}{c} \bar{Local\text{\_}FE_{start}}=\left(\bar{FE_{2}},\bar{FE_{3}},\bar{FE_{4}},...\right)\\ \bar{Local\text{\_}FE_{end}}=\left(\bar{FE_{n}},\bar{FE_{n-1}},\bar{FE_{n-2}},...\right) \end{array}$$

where:

$$\bar{FE_{i}}=\underset{Genes_{i}}{\sum }FE_{i}∕\left|Genes_{i}\right|$$

and *Genes_i _*is the number of genes with at least *i *+ 1 40-nucleotide windows.

Local profiles of amino acid charge were computed in a similar way. First, we computed for each gene a vector of the charge assigned to the amino acids of the gene (+1 for a positive charge, -1 for a negative charge, 0 for a neutral amino acid). Next, we lined up the genes once according to their start codon, and once according to their stop codon, and computed the mean charge at each position.

The local profiles of tAI were computed in a similar way [8]. First, we computed for each gene a vector of the tAI assigned to the codons of the gene. Next, we lined up the genes once according to their start codon, and once according to their stop codon, and computed the mean tAI at each position (codon).

When we computed the reported profiles we considered all the genes (that is, we did not filter short genes) as we believe that the important feature in our context is the distance from the ATG in codons and not in percentage of the coding sequences (see also [8]). Most of the analyzed genes are longer than 200 codons; in *S. cerevisiae*, for example, less than 20% of the genes are shorter than 200 codons (1,119 out of 5,861; a histogram of the *S. cerevisiae *gene lengths is shown in Figure S46 in Additional file 2).

### Computing the profile of ribosomal density

The data for the ribosomal density profile were kindly supplied to us by Dr Ingolia. He sent us the data that were used for generating Figure 2d in their paper [15]. The data included read density at single nucleotide resolution as a function of position along the gene for well-expressed genes. The read density of each gene was normalized compared to itself (see details in [15]). We averaged the ribosomal density values of the nucleotides of each codon (as the data of Ingolia *et al*. was at the at single nucleotide resolution) in each gene to obtain a per-codon measurement of ribosomal density. The genomic ribosomal density profile was computed in a way similar to the tAI, folding energy and charge profiles (the values of each codon were averaged over all the relevant genes).

### Profiles of tRNA adaptation index, folding and charge for groups of genes

Profiles of coding sequence determinants for specific gene groups (for example, ribosomal proteins and GO slim groups) were computed as reported above. In these cases, however, we only considered the genes in the group.

### The tRNA adaptation index as a predictor of protein abundance

Highly expressed genes have more efficient codons to improve their translation rate, the allocation of ribosomes, and the fitness of the organism [7,14]. Thus, it is not surprising that in many organisms measures of codon bias such as the tAI exhibit significant correlation with protein abundance [5,14,40,47]. In *S. cerevisiae*, for example, the correlation between tAI and protein abundance is higher than 0.6 [5,40].

### Linear regression and partial correlations

Let *X *and *Y *denote two variables and *Z *= [*Z1*, *Z2*, *Z3*,..] denote a set of variables. The non-parametric multivariate analysis that is reported in this paper includes partial Spearman correlations of the from *R*(*X,Y|Z*). Roughly, if such a correlation is significant, it means that there is a relationship between *X *and *Y *that can not be explained by the variables in *Z*. Specifically, we computed the correlation between ribosomal density (*X*) and one of the three coding sequence determinants (*Y*; tAI, charge, or folding energy) given the rest of the coding sequence's determinants. This analysis was performed by the commercial MATLAB software (see more details in MATLAB help. Founded in 1984, MathWorks employs 2200 people in 15 countries, with headquarters in Natick, Massachusetts, USA).

Let *rxy *denote the matrix of the correlation coefficient corresponding to the vectors *x *and *y*. The partial correlation for two variables (*x *and *y*) when controlling for a third variable (*z*), *rxy_z*, is computed according to the following formula (see, for example, [48]):

(1) $$rxy\text{\_}z=\frac{rxy-rxz*ryz}{\sqrt{\left(1-{\left(rxz\right)}^{2}\right)*\left(1-{\left(ryz\right)}^{2}\right)}}$$

When we want to control for more than one variable we can use the formula above in a recursive way. For example, the correlation between *x, y *when controlling for *z *and *w *(the case that was reported in the paper) is:

(2) $$rxy\text{\_}zw=\frac{rxy\text{\_}z-rxw\text{\_}z*ryw\text{\_}z}{\sqrt{\left(1-{\left(rxw\text{\_}z\right)}^{2}\right)*\left(1-{\left(ryw\text{\_}z\right)}^{2}\right)}}$$

In Equation 2, *rxy_z*, *rwy_z*, *ryw_z*, *rxw_z*, and *ryw_z *are computed using Equation 1.

The *P*-values are computed for linear and rank partial correlations using a Student's *t *distribution for a transformation of the correlation. This is exact for linear partial correlations when the variables are normal, but if this is not the case it is a large-sample approximation. We also computed an empirical *P*-value that was based on 100 permutations of *x *and *y *(the variables that are not controlled for). The empirical *P*-value is the frequency of the times that the partial correlation of the permutated vector was larger than the original one (it was significant for all variables).

The regressor mentioned in the main text is a linear regressor, where the explained variable is the ribosomal density and it is explained by the three coding sequence determinants (tAI, charge, and folding energy). As we mentioned above, the ribosomal density data are noisy; thus, we utilized the smoothed version of all the profiles (five-point moving average; the default parameter in MATLAB), but obtained very similar results without smoothing.

### Simulation of ribosomal movement

The ribosomal movement model was based on the work of [34] (Figure 4c). According to this model, the nominal translation time of a codon is determined by the charge of the amino acid encoded by the codon (and the charge of the amino acids encoded by the neighboring codons upstream), the co-adaptation of the codon to the tRNA pool, and the strength of mRNA folding near (upstream of) the codon (see more details in the next section).

The actual translation time of a codon is also related to the potential presence of a ribosome downstream of it. If there is a proximal ribosome in front of it, the ribosome translating the codon is delayed until the ribosome downstream of it proceeds.

Other parameters of the simulations are: the minimum distance between two consecutive ribosomes (that is, the size of the ribosome); the ribosome binding time (initiation time); and the termination time (the time required for the ribosome to release the mRNA). The properties (for example, translation time) of the ribosome movement regime were computed at steady state (that is, when there was a negligible change in the translation time between consecutive ribosomes and after at least one ribosome completed the translation).

### Stochastic model of translation elongation

This model is based on [36]. We model an mRNA with *N *codons as a chain of sites, each of which is labeled by *i*. The first and last codons, *i *= 1, *i *= *N*, are associated with the start and stop codons, respectively. At any time, *t*, attached to the mRNA are *M*(*t*) ribosomes. Each ribosome will cover *l *codons. Any codon may be covered by a single ribosome or none. To locate a ribosome, we arbitrarily assume that the codon being translated is the one in the middle of the ribosome. For example, if the first (*l + *1)/2 codons are not covered, a ribosome can bind to the first codon on the mRNA strand, and then it is said to be 'on codon *i *= 1'. A complete specification of the configuration of the mRNA strand is given by the codon occupation number: *n_i _*= 1 if codon *i *is being translated and *n_i _*= 0 otherwise. Note that when *n_i _*= 1 the (*l - *1)/2 codons before and after codon *i *are covered by the ribosome that is on site *i *but since they are not the ones being translated the codon occupation number for them is equal to zero.

We will now specify the dynamics of this model. A free ribosome will attach to codon *i *= 1 with rate *λ*, provided that the first (*l + *1)/2 codons on the mRNA are empty. An attached ribosome located at codon *i *will move to the next codon *i *+ 1 with rate *λ_i_*, provided codon *i *+ (*l + *1)/2 is not covered by another ribosome. In case *i *+ (*l + *1)/2 >*N *(the ribosome is bulging out of the mRNA strand) an attached ribosome will move to the next codon with rate *λ_i_*. The translation rates *λ_i _*are inversely proportional to the mean translation times *t_i_*.

In order to simulate these dynamics, we assume that the time between initiation attempts is distributed exponentially with rate *λ*. Similarly, the time between jump attempts from site *i *to *i + *1 is assumed to be exponentially distributed with rate *λ_i_*. Note that in the case of *i *= *N *the jump attempt is in fact a termination step. We define an 'event' as an initiation, jump attempt, or termination step. From our definition it follows that the time between events is exponentially distributed (minimum of exponentially distributed random variables) with rate:

$$\mu \left(\left\{n_{i}\right\}\right)=\lambda +\overset{N}{\underset{i=1}{\sum }}n_{i}\lambda_{i}$$

Note that a jump attempt from codon *i *can only be made if there is a ribosome translating this codon and hence the rate *μ*({*n_i_*}) depends on the set of site occupation numbers.

The probability that a specific event was an initiation attempt is given by *λ/μ*({*n_i_*}). Similarly, the probability that a specific event was a jump attempt (or termination event) from site *i *to site *i *+ 1 is given by *n_i_λ_i_*/*μ*({*n_i_*}).

At each step of the simulation, we determine the nature of the event and the time passed till its occurrence by these rules. The set of site occupation numbers is then updated accordingly and the simulation proceeds to the next event. For example, if an initiation attempt was made, we check if the first (*l *+ 1)/2 codons on the mRNA are not covered. If so, we set *n_i _*= 1, otherwise the attempt fails and *n_i _*remains as is. If a jump attempt from codon *i *to codon *i *+ 1 was made, we check if site *i *+ (*l *+ 1)/2 is not covered. If so, we set *n_i _*= 0 and *n*_*i*+1 _= 1, otherwise the attempt fails and *n_i_*, *n*_*i*+1 _remain as is.

Starting with an empty mRNA strand we simulated the system for 250,000 steps. The system was then simulated for an additional 1,000,000 steps where we kept track of the total number of terminations and the total time that have passed from the point this phase started. The steady state rate of protein production was determined by dividing the number of termination events by the total time that has passed. The number of steps in the second stage was taken after observing that increasing the number of steps fourfold had a negligible effect on the predicted protein production rate.

### Simulation of ribosomal movement: translation time of a codon

The translation time (or rate) of a codon (or the nominal speed of its translation) is based on three features of the coding sequence. First is the co-adaptation of the codon to the tRNA pool; this value was based on the tAI. Second is the charge of the amino acids corresponding to the 31 neighboring upstream codons. The exit tunnel of a ribosome has negative charge and its length is around 31 amino acids [24]; thus, amino acids with positive charge should slow the translation time of a ribosome [24]. At the beginning of the gene we considered the *l *< 32 amino acids before the codon. Third is the folding energy of the neighboring downstream mRNA (40 nucleotides from the start of the codon). Stronger folding should slow the ribosome [49,50]. When the ribosome translates a codon the A site of the ribosome lies in the middle of a stretch of mRNA that is physically occupied and unwound by the ribosome; however, we are interested in modeling the delay/speed of the ribosome when it is translating this codon. At this stage, the mRNA folding before the ribosome is not relevant (the ribosome already translated these codons); the mRNA folding after the ribosome is relevant as this part of the mRNA should be unfolded by the helicase before the ribosome continues and moves forward.

The non-normalized time corresponding to the adaptation to the tRNA pool of the organism (tAI*i*) of codon *i *is: 1/tAI*i*.

The non-normalized time corresponding to the charge upstream of codon *i *is the sum of 'amino acid charges' among the 32 amino acids before the codon (where a neutral amino acid adds 0 to the sum, a positively charged amino acid adds 1 to the sum, and a negatively charged amino acid adds -1 to the sum; the amino acids with positive charge are Arg and His, and Lys, while the amino acids with negative charge are Asp and Glu).

The non-normalized time corresponding to the folding energy downstream of codon *i *is the folding energy of the 40-nucleotides starting from the beginning of the codon (at the end of the sequence consider the 3' UTR).

The three normalized times were computed as follows: for each of the three features we divide their non-normalized value by their mean value along all the coding sequences and all windows, such that the mean of each of the normalized features will be 1.

Let *Ntai*(*i*) denote the 'normalized tRNA pool adaptation time' of codon *i*; let *Nch*(*i*) denote the 'normalized charge time' of codon *i*; let *Nfe*(*i*) denote the 'normalized folding energy time' of codon *i*.

The total time corresponding to the the *i*-th codon is $\frac{a1}{Ntai\left(i\right)}\cdot e^{a2\cdot Nch\left(i\right)+a3\cdot Nfe\left(i\right)}$.

We checked *a1*, *a2*, *a3 *in the range 0[1] and chose the values that optimized the correlation between the prediction of the ribosomal movement model (with the times above) and the actual ribosomal density. The correlation was based on the smoothed version of the real and predicted profiles (five-point moving average).

We obtained similar correlations when we used charge and folding before or after the codon, probably since the charge and folding in close windows in a gene tend to be similar and thus correspond to relatively similar speeds.

### The size of the ribosome

Based on previous studies [8,15,30,34,51], the footprint of the ribosome on the transcript is 10 to 20 codons. As was mentioned before, the exit channel, which is in a different compartment of the ribosome, is longer (31 codons).

### Profiles of mRNA secondary structure robustness

An mRNA sequence is robust to errors (point mutations) if point mutations tend to maintain its two dimensional structure (compared to random sequences with similar features).

We computed profiles of secondary structure robustness by performing the following steps for each window of length 40 nucleotides in each mRNA sequence. First, compute the folding structure and folding energy for each of the 40 × 3 one-nucleotide point mutations of the sub-sequence. Second, compute the distance of each mutated sequence from the original one in terms of absolute change in folding energy and the number of changes in the base-pair connections required for transferring one structure to the other (see, for example, [33]); we also plotted the mean number of point mutations (errors) that do not change the mRNA structure. Third, average the distances for each window.

As a control, we generated a randomized genome maintaining the codon bias and the amino acid sequences in the original genome. We compared the distribution of robustness obtained in the original genome and the randomized one.

To control for folding energy we divided the windows into five groups of equal size; each group includes windows (over all genes) with similar folding energy. We plotted the profiles of folding energy robustness for each group separately.

To manage the extensive amount of computations needed for performing so many predictions of secondary structure, we employed a cluster of eight computers (each of which had an AMD Opteron(tm) 252 2. GHz processor, two cores, and 6 GB RAM) and a six-core computer (AMD Phenom(tm) II X6 1090T 3.2 GHz processor, six cores, 16 GB RAM) for several weeks.

### Profiles of tRNA adaptation index and charge robustness

tRNA adaptation index and charge robustness profiles were computed in a similar manner. In the case of the charge, we computed for each window of 13 codons the number of point mutations that change the charge of the corresponding amino acids, and the mean change in the charge due to point mutations. In the case of the tAI, we computed for each window of 13 codons the average (over all point mutations) change in the tAI score of the codon, and the number of mutations that do not change the tAI of the codon. At the next step, we plotted the corresponding genomic profiles of robustness as was described for the folding energy robustness.

### Robustness profiles: control for folding, tRNA adaptation index, and charge

To make sure that the robustness profiles are not trivially a result of the fact that the folding, tAI, and charge values are more extreme at the beginning of the coding sequences, we also analyzed the robustness profiles when considering only windows in certain ranges of folding, tAI, and charge, respectively (five bins of equal size).

It is also important to note that the codons (and similarly the folding or the charge) at the beginning of the coding sequence are less optimal than those at the end of it; that is, relative to the immediate context, these codons are not necessarily the universally least efficient ones. For example, in highly expressed genes, these codons can be more efficient than all the codons of lowly expressed genes (Figure 2c).

### Robustness profiles: assuming different probabilities of transition and transversion errors; assuming that translation errors are relatively rare in the second position of the codons

To consider the fact that transcription errors that result in transition may have higher probability than transcription errors that result in transversion, we gave higher weights to the first type of errors when we computed the robustness scores. For example, if we assume that the probability of a transition error is twice the probability of a transversion error, the weight of such an error/mutation in the folding/charge/tAI robustness score of an mRNA window is two times the weight of transversion.

Similarly, in the case of charge robustness, to consider the fact that translation errors are very rare in the second position of codons [35], the weight of such an error/mutation in the charge robustness score of a mRNA window is lower (for example, 0.1) than the weight of an error in the first/third positions of the codons.

### The length of the ramps

The length of the ramp (for a profile of tAI, charge, folding energy, or robustness) was computed similarly to [8] by comparing the mean (KS test) of sliding windows of length 13 codons to the mean of the rest of the corresponding profile (we considered the first 200 codons). The region at the beginning corresponding to a set of consecutive windows with a mean value significantly lower (*P *≤ 0.05) than the mean of the entire profile was defined as the length of the ramp (the length of the ramp is the number of significant consecutive windows plus 12). We allowed this region to begin in the first five codons (if there was no significant window in this region, we declared that there is no ramp).

### *P*-values for the folding, charge, and tAI profiles and the corresponding robustness profiles

We performed two statistical tests to check if the coding sequence determinants of a certain position were significant.

In the first test we checked if the value is more extreme than in other positions. This test does not take into account constraints on amino acid sequences. Firstly, however, we believe that selection for translation efficiency can also occur at the amino acid level - that is, there are many pairs of amino acids that, when substituted, do not change the function of the protein but can improve translation (see, for example, [52-54] about various distances between amino acids). The effect on translation and the coding sequence function is determined by the position of the codon along the coding sequence. Secondly, the effect of the various features of a codon on the translation rate of the ribosome can be significant, even though these profiles are not selected for.

The first test was performed by comparing (KS test) all the values in the positions within the ramp (see the previous section) to the rest of the positions. A similar test was performed also while testing subgroups of genes (for example, ribosomal proteins and GO groups).

In the second test we checked if the value is more extreme than the randomized version of the position. The randomized version of the genome was generated by maintaining the amino acid composition of each coding sequence and the codon bias of the genome, and sampling for each gene, a randomized version under these constraints. In this case, we compared the values of the positions within the ramp of the real genome and the randomized one by a KS test.

### Predicted ribosomal density versus real ribosomal density in single genes

We used the linear regressor and the model that gave the best genomic ribosomal density to predict the ribosomal density at a resolution of single codons in each of the *S. cerevisiae *genes. Next, we plotted the graph (histograms) of distances between the window with the highest ribosomal density, and the window with highest predicted ribosomal density. We show that the distances tend to be small. We compared the distribution of distances corresponding to the real genome to a randomized genome, where the vectors of ribosomal densities were randomly permutated and show (by KS test) that the original mean is significantly smaller than the randomized mean.

### Cross-validation tests and evaluation of the ribosomal movement model and the regressor

We compared the model and regressor with all three features to models that include only some of the features (for example, only charge and tAI without folding) by performing 20 cross-validation tests. In each test the model was trained based on 50% of the data (training sets) and was implemented on the second 50% of the data (test sets). We performed correlations between the ribosomal density and the predicted ribosomal density (by the model or the regressor) for the test sets in the full model and compared them to the correlations obtained for the partial models. The empirical *P*-value was computed as percentage of cases where the partial model was better then the full one on the same test set. Differences (between pairs of correlations) larger than 0.4% were assumed to be significant. We also compared, in a similar manner, the absolute sum of distances between the predicted and real genomic profile of ribosomal density.

In the case of the predicted profile of ribosomal density in single genes, we divided the genes into two groups as before, inferred the parameters of the model based on one of the groups and applied it to the second group (as reported above).

### Site-by-site comparisons of predicted versus real ribosomal density

We wanted to test whether there is a direct relationship between the coding sequence determinants and the ribosomal translation rate (speed). Thus, we aimed at showing a significant relationship between local density of ribosomes [15] and charge, tAI, and folding profiles in single genes. Such a task comes with several caveats: 1) as mentioned, the measurements of ribosomal density are very noisy; 2) ribosomes may interact with each other (jam); 3) often, the rate-limiting step may be initiation, which varies across genes.

To overcome these problems, we searched for the window with the highest ribosomal density in each individual gene and compared its position to the position with the slowest translation rate based on the three genomic features. This relationship should hold also when initiation is rate limiting and varies among genes, or when there are interactions between ribosomes.

### Randomized profiles

To show that the genomic profiles reported in this study (the three ramps at the beginning of genes and the increased robustness to transcription errors at the beginning of genes) are not due to amino acid bias, we compared the genomic profile of folding energy with a profile of folding energy observed for a randomization of the genome. The genome was randomized in the following manner. Each codon was replaced by a random codon, according to the distribution (frequency) of codons coding the same amino acid in the genome of the organism. Thus, the randomized genomes maintained both the amino acid content of each coding sequence and the codon frequencies of the original genome.

### Genomic profiles based on measurements of mRNA folding

We downloaded the mRNA measurement data from [28]. These data include for each nucleotide, in thousands of *S. cerevisiae *transcripts, the log ratio between the probability that it is in a double-stranded conformation and the probability that it is in a single-stranded conformation (parallel analysis of RNA structure (PARS) score [28]). If this value is higher, the position is involved in a double-stranded conformation and this is related to a higher folding energy. At the first stage (Figure S2 in Additional file 2), we computed for each position the mean PARS score (over all the genes) and plotted two profiles: 1) a simple average; and 2) a weighted average (in which the weight of G or C, which are involved in pairings with three hydrogen bonds, is 3, while the weight of A or T, which are involved in pairings with two hydrogen bonds, is 2). We performed a KS test and compared each position to the remaining 600 'first' positions along the genes. We found that the first three positions have a significantly low PARS score (weak mRNA folding) while the next two positions have a significantly high PARS score (strong mRNA folding).

At the second stage we plotted this profile for highly expressed genes (top 10% of the genes in terms of mRNA-levels × ribosomal density) and lowly expressed genes (bottom 10% of the genes in terms of mRNA-levels × ribosomal density) and demonstrated that the high/low PARS score reported above is stronger for highly expressed genes (Figure S2 in Additional file 2; similar results were obtained for the weighted profile).

One problem of the PARS score is that it is global and not local (like the predicted local folding energy measure reported in this study) - a nucleotide has a higher PARS score even if it is connected to another nucleotide inside or outside the 40-nucleotide window. Thus, we used the inferred folding of complete mRNA sequences of yeast based on the PARS scores that are reported in the study of Kertesz *et al*. [28]. We computed for each sliding window in each gene how many pairs of nucleotides where both nucleotides are within the window are connected. We plotted the mean genomic graph of these values (as we did with the predicted folding energy). The new graphs indeed look similar to the graph obtained based on predictions of mRNA folding (Figure S2 in Additional file 2).

### Genomic profiles of pairs of identical slow codons

To study the distribution of pairs of identical slow codons along the coding sequences, we divided the codons into slow (the lowest 10% in terms of the tAI) and fast ones (the remaining codons). We computed the profile of the mean number of pairs of identical slow codons in each position in *E. coli *and *S. cerevisiae*. We compared this profile to those obtained under two randomization regimes: 1) when controlling for amino acid content and codon bias as mentioned above; and 2) when permutating only the slow codons in each gene (that is, a control that considers the fact that there are positions, such as the ramp, with more slow codons). We also computed this profile separately for highly and lowly expressed genes in these organisms.

## Abbreviations

GO: Gene Ontology; KS: Kolmogorov-Smirnov; ORF: open reading frame; PARS: parallel analysis of RNA structure; tAI: tRNA adaptation index; UTR: untranslated region.

## Authors' contributions

TT designed the experiments, conducted the data analysis and wrote the manuscript. IV conducted the data analysis and helped to write the manuscript. NG conducted the data analysis. MK helped to write the manuscript. ER helped to write the manuscript. MZ helped to write the manuscript. ER and MZ contributed equally to this work. All authors have read and approved the manuscript for publication.

## Supplementary Material

Additional file 1 **Supplementary Notes S1 to S5**: [55-61] Click here for file

Additional file 2 **Supplementary Figures S1 to S7 and S9 to S46**. Click here for file

Additional file 3 **Supplementary Table S1 - properties of ramps for GO groups**. Click here for file

Additional file 4 **Supplementary Figure S8**. Click here for file
