# Supplementary material for: Composite effects of gene determinants on the translation speed and density of ribosomes
Source: Genome Biol. 2011 Nov 3;12(11):R110. doi: 10.1186/gb-2011-12-11-r110 (PMC3334596; doi:10.1186/gb-2011-12-11-r110)
Supplement: Additional file 1 — Supplementary Notes S1 to S5: [55-61] [file gb-2011-12-11-r110-S1.PDF]

Note S1: the justification for using the tAI as an predictor of the co-adaptation between codon bias and tRNA pool

The tAI are based on the genomic tRNA copy number (tGCN) as a surrogate measure for the cellular abundances of tRNAs (as the expression of tRNA genes is unknown); it is justified by several observations.

First, in the past, in many organisms, it has been observed that the *in vivo* concentration of a tRNA bearing a certain anticodon is highly proportional to the number of gene copies coding for this tRNA type. Specifically, in *S. cerevisiae* a correlation of up to r=0.91 [8, 55] was reported. In *B. subtilis*, a correlation of 0.86 between tRNA copy number and tRNA abundance was reported [56]. Similarly, previous papers reported about significant correlation between genomic tRNA copy number and tRNA abundance in *E. coli* [57, 58]. A related interesting result is the analysis of [59] who measured the translation rate of two glutamate codons: GAA and GAG. They found them to have a threefold difference in translation rate (21.6 and 6.4 codons per second, respectively). Remarkably, the w_i of these codons, which is based on the tRNA pool and affinity of codon-anti-codon coupling and is the basis for the tAI calculation, captures the ratio of translation rate between the two codons. Calculating w_i values for *E. coli*, we found that the ratio between the w_i of GAA and GAG is 3.125 (0.5/0.16) as compared to the 3.34 reported in the experiments (21.4/6.4). This result suggests that there is a direct relation between the adaptation of a codon to the tRNA pool, based on the genomic tRNA copy number, and the time it takes to translate it.

Second, a recent study showed that in *S. cerevisiae* the promoters of many of the tRNA genes have a low predicted affinity to the nucleosome, suggesting a constitutive expression with little transcriptional regulation capacity [60]. Thus, for fully sequenced genomes, the relative concentrations of the various tRNAs in the cell, and therefore the optimality of the various codons in terms of translation, can be approximated using the respective tRNA gene copy numbers in the genome. Additionally, as was shown in previous papers, measures that are based on tRNA copy number are highly correlated with protein expression levels (see, for example,

[5, 40]). It was found that even among genes with similar transcript levels, higher tAI often corresponds to higher protein abundance [40].

Note S2: Genomic profiles at the end of genes.

We also computed the three profiles after aligning the coding sequences to the stop codon (Methods). At the end of the coding sequences the signals were less clear suggesting that the important region in our context (translation elongation) is the beginning of the coding sequences (see for example, supplementary figures 33-39).

For example, *in S. cerevisiae*, the differences between highly expressed genes and lowly expressed genes remain for the tAI profile (*only* highly expressed genes have more efficient codons at the end of genes). In the case of the charge, there is *small* region (a few codons) with *positive* charge at the end of genes. In the cases of the folding energy there is small region of lower folding energy at the end of all genes, but this region is probably related to amino acid bias [14] (see supplementary figures 33-35).

In addition, the mean average absolute difference in the energy between the randomized coding sequences and real coding sequences is lower at the few last codons at the end of genes (see supplementary figures 36) but when we control for the folding energy most of the signal disappears (supplementary figures 37). Both the the tAI and the charge at the end of the coding sequences tend to be less robust (supplementary figures 38-39). All together, the results suggest that the robustness in terms of charge, tAI, and folding is more important at the beginnings of the coding sequences.

Note S3: Potential advantages of the ramp for genes with lower ribosomal density

A ramp of slower translation rate at the beginning of the coding resgion has advantages also for genes with low ribosomal density. For example, such a ramp may effectively increase the initiation time of the gene and thus, as there are more available ribosomes, the global allocation of ribosomes.

In addition, slow codons increase the probability of ribosomal abortion without relation to the number of additional ribosomes on the transcript. Thus, slow codons that are *not* at the beginning may result in abortion after translating longer peptides – a situation that 'costs' more energy (in comparison to the case where the slow codons are at the beginning of the ORF - ramp). Thus, there is stronger selection *against* slower codons *after* the ramp, also in genes that are *not* occupied with many ribosomes.

Other advantages of the ramp that are not necessarily related to high ribosomal density includes: 1) initiation control -- couple between initiation and elongation; 2) Gene-specific and condition-specific control (see discussion with more explanations in [8]).

Note S4. The correlations between the three genomic features for randomized sequences is low.

We analyzed the correlation between charge, folding energy and codon bias for 10,000 random sub-sequences of length 13 codons that were sampled from the coding sequences of *S. cerevisiae, E. coli:, and C. elegans*. In all cases, the correlations were very low:

In *S. cerevisiae: R(charge,tAI)* = -0.1026 *(p* < 10^{-16} *); R(charge,Folding energy)* = 0.0888*; (p* < 10^{-16}*); R(Folding energy,tAI)* = -0.0245 *(p = 0.014).*

In *E. coli: R(charge,tAI)* = 0.0075*(p=0.45); R(charge,Folding energy) =- 0.0052 (p =0.6); R(Folding energy,tAI) =-0.0071(p = 0.47).*

In *C. elegans: R(charge,tAI)* = -0.0547 *(p = 4.3**10^{-8}*); R(charge,Folding energy) =0.102 (p* < 10^{-16}*); R(Folding energy, tAI) = -0.102 (p* < 10^{-16}*).*

Note S5. Genomic profiles of pairs of identical slow codons.

We believe that there are additional genomic profiles that apply to translation, which have not formerly been reported. For example, one such profile is related to slow consecutive identical codons. Such pairs of codons should have an increased effect on the deceleration of translation rates, and it was also suggested that such pairs of

codons have an effect on frame shifts [61]. In *E. coli* such pairs of identical slow codons tend to appear more frequently at the beginning of the coding sequence to increase the effect of the ramp. However, in highly expressed genes this signal is lower than in randomized sequences (probably to prevent frame-shifts; see Methods, supplementary figures 40-45).
