# Supplementary material for: Composite effects of gene determinants on the translation speed and density of ribosomes
Source: Genome Biol. 2011 Nov 3;12(11):R110. doi: 10.1186/gb-2011-12-11-r110 (PMC3334596; doi:10.1186/gb-2011-12-11-r110)

Supplementary Figure S1. Median profile of local folding energy in *S. cerevisiae*. The profile was computed in a similar way to the mean profile reported in the main text but in each position we took the median instead of the mean.
As can be seen, the profile is similar to the one reported in the main text for the mean. This result demonstrates that the folding 'ramp' of stronger mRNA folding at the beginning of genes is not due to small number of genes.

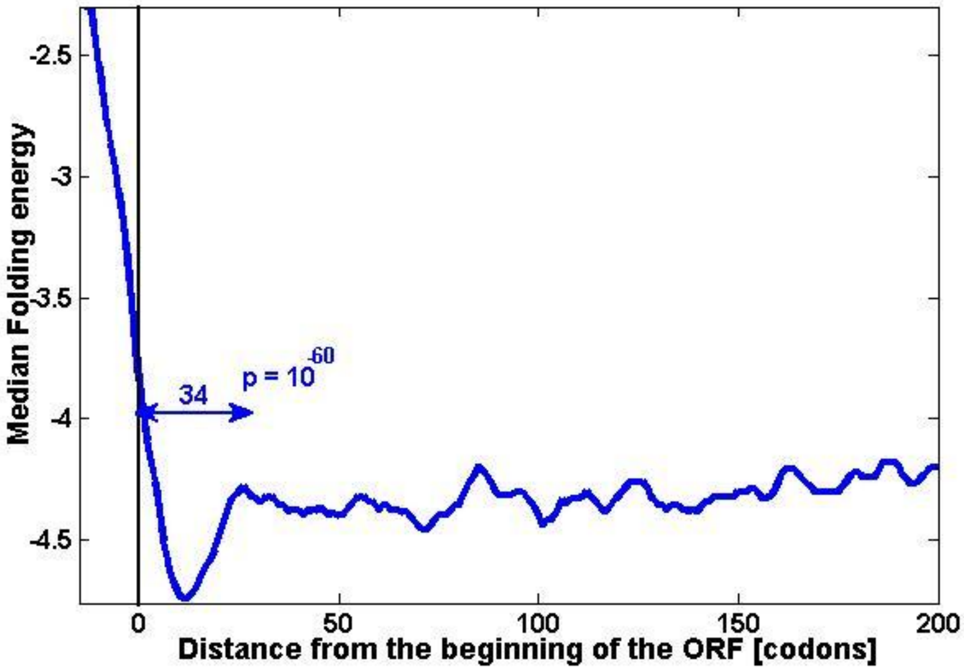

Supplementary Figure S2. Genomic profiles based on folding energy *measurements* in *S. cerevisiae*. A. Genomic profile of the number of pair-bases that are connected *within* each window based on the folding of entire mRNA from (Kertesz et al., 2010 (Methods). There is a region (24 codons) with larger local folding (more bp-connections within a window) at the beginning of the ORF (similar to the one reported in figure 1A) B.-C. The genomic profile as in A. but for genes with high ribosomal density (red; top 10%) and genes with low ribosomal density (green; bottom 10%) (B) and for genes with high (mRNA levels)*(ribosomal density) (red; top 10%) and genes with low (mRNA levels)*(ribosomal density) (green; bottom 10%). As in Figure 2; the ramp is longer for genes with high ribosomal density and mRNA levels)*(ribosomal density). D.-E. The PARS (Methods) score of the nucleotides near the beginning of the ORF has a distribution that may explain A-D. Higher values denote higher probability to be in a double- strand conformation (*i.e.* stronger folding). Significant p-value are marked in the figures. D. The overall genomic profile of energy measurements when weighting G-C content (blue; Methods) and not weighting G-C content (green). In both cases the first three positions have significantly low PARS score (Methods) and the two points afterwards have significantly high PARS score. E. The mean profile of energy measurements for highly expressed genes (red; Methods) and lowly expressed genes (green; Methods) the signal of low PARS score followed by a high PARS score is stronger for highly expressed genes.

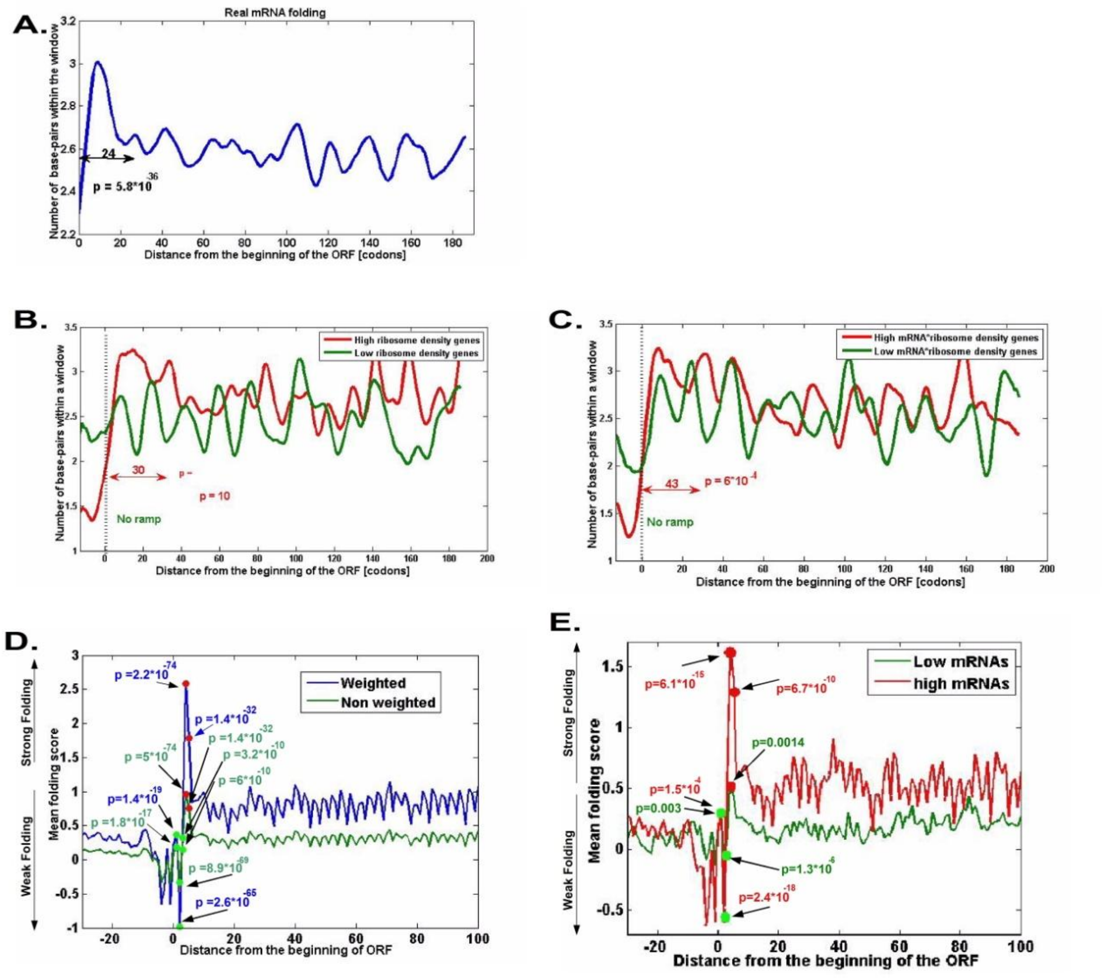

Supplementary Figure S3. Genomic profiles of charged amino acid (AA) in *S. cerevisiae*. *Lys* and *Arg* (AA with positive charge) tend to have higher frequency at the beginning of ORFs while the AA with negative charge (*Asp*, *Glu*) have lower frequency at the beginning.

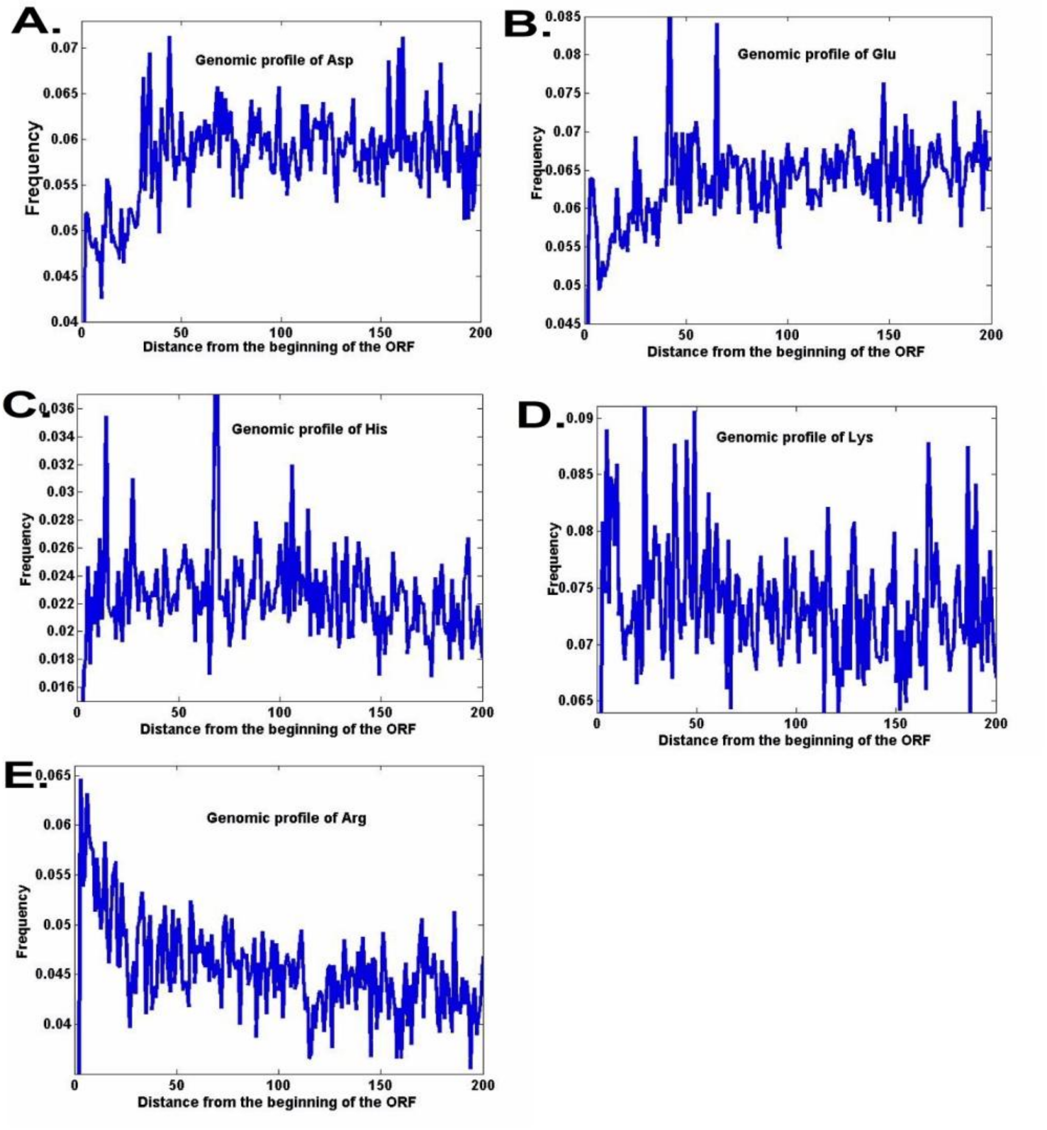

Supplementary Figure S4. The profiles of charge (A.), folding energy (B.) and co-adaptation between the codon bias and the tRNA pool (C.) for genes with high mRNA levels (red; top 15%) and genes with low mRNA levels (blue; lowest 15%) in *E. coli*.

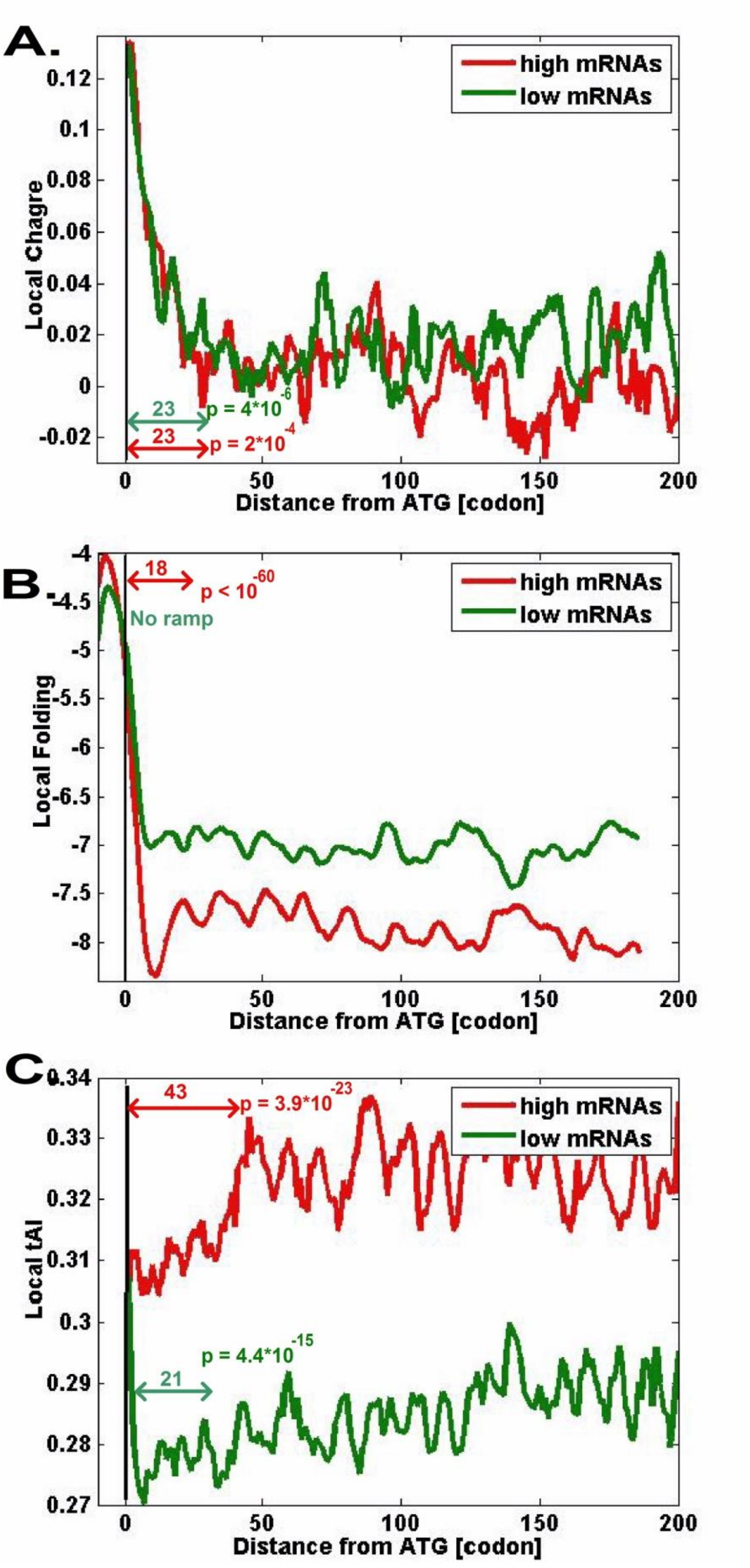

Supplementary Figure S5. The profiles of charge (A.), folding energy (B.) and co-adaptation between the codon bias and the tRNA pool (C.) for genes with high mRNA levels (red; top 15%) and genes with low mRNA levels (blue; lowest 15%) in *C. elegans*.

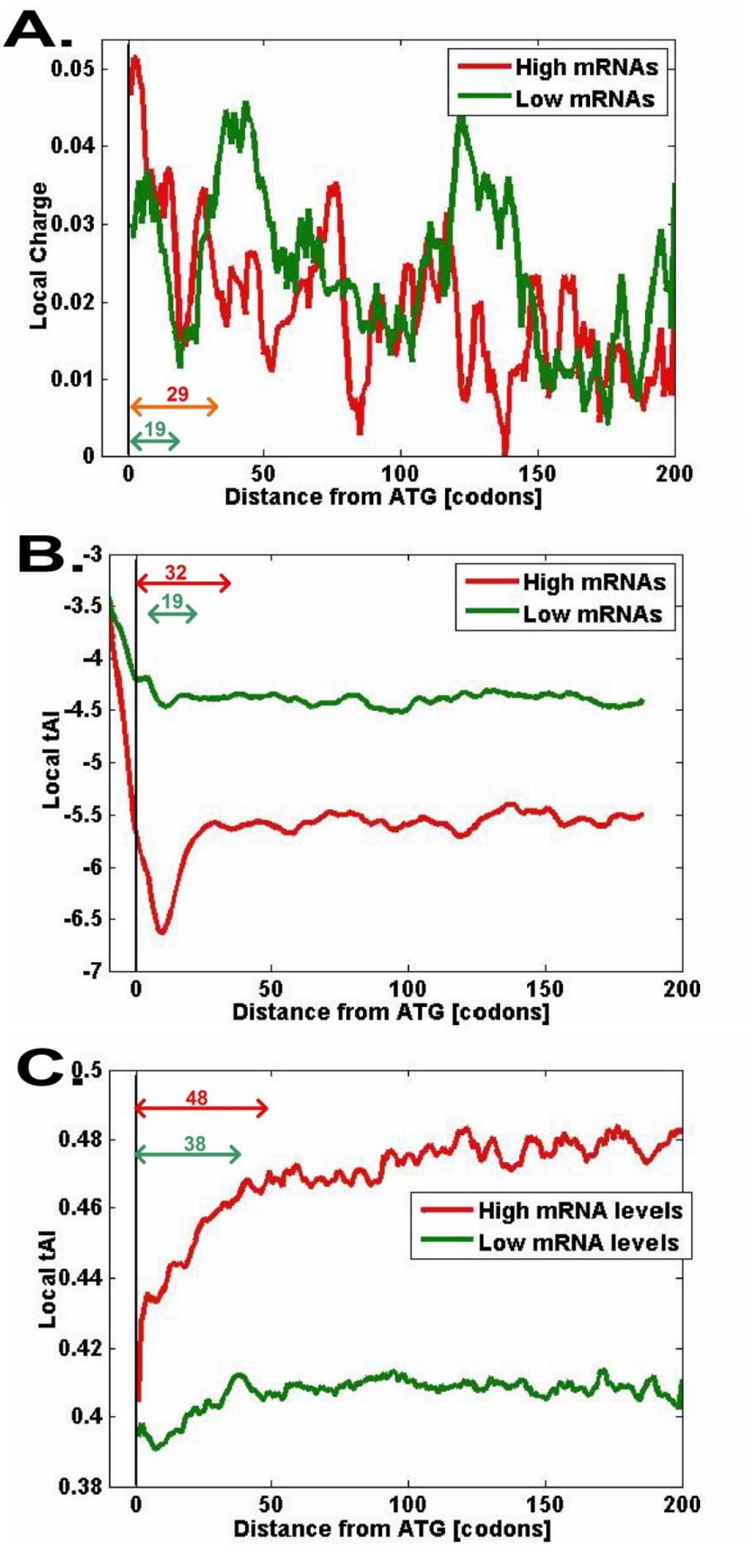

Supplementary Figure S6 - The profiles of charge (A.), folding energy (B.), tAI (C.) for cytosolic ribosomal proteins.

A.

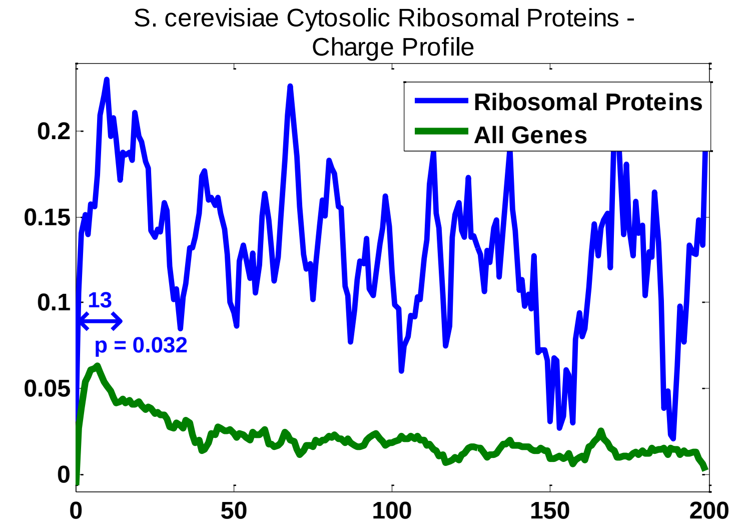

B.

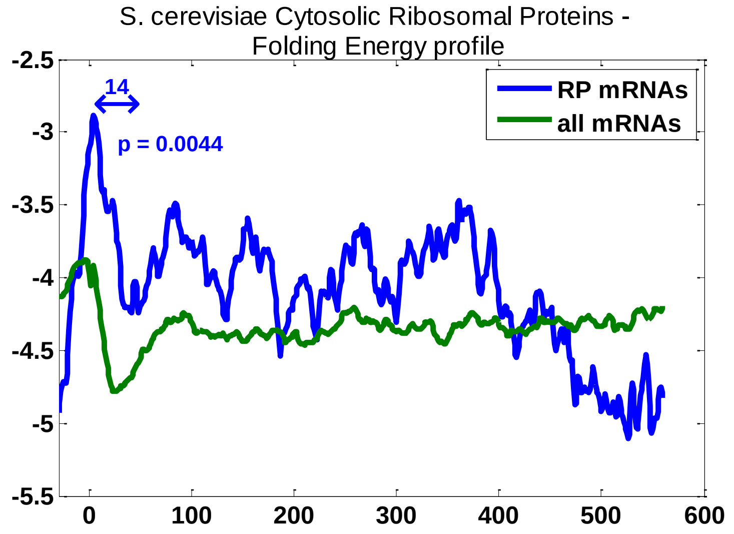

C.

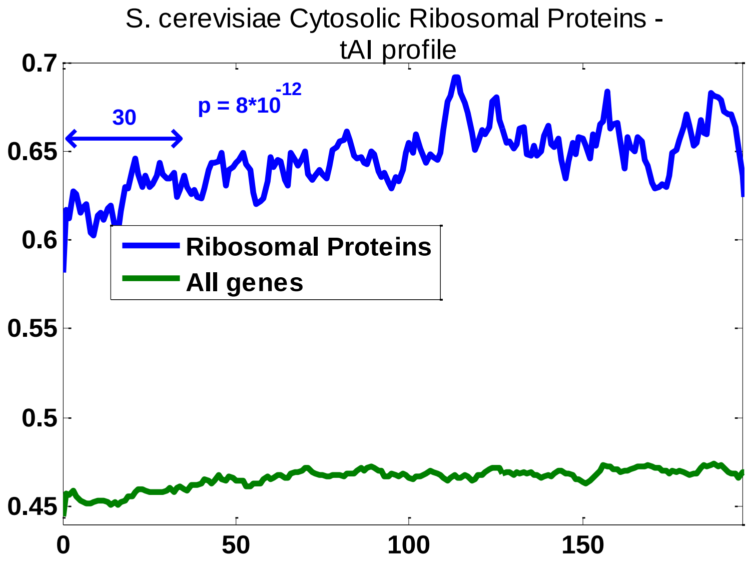

Supplementary Figure S7 - The profiles of charge (A.), folding energy (B.), tAI (C.) for mitochondrial ribosomal proteins.

A.

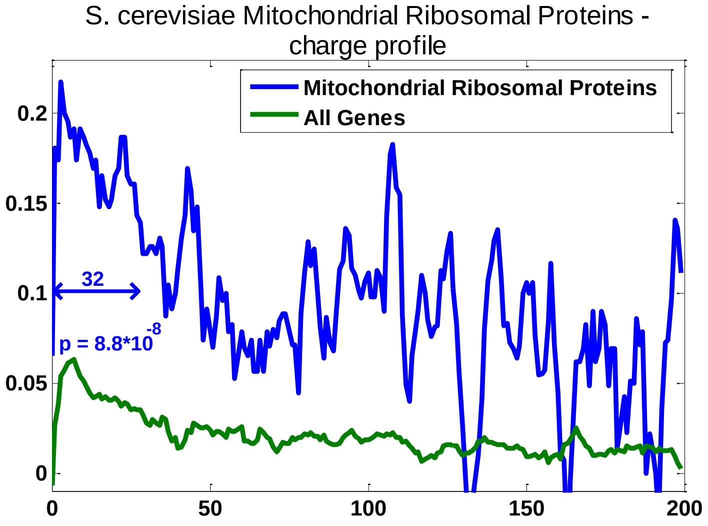

B.

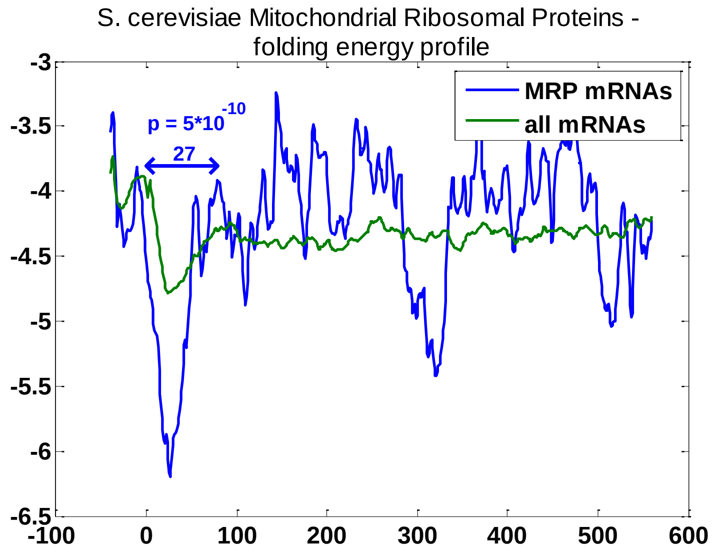

C.

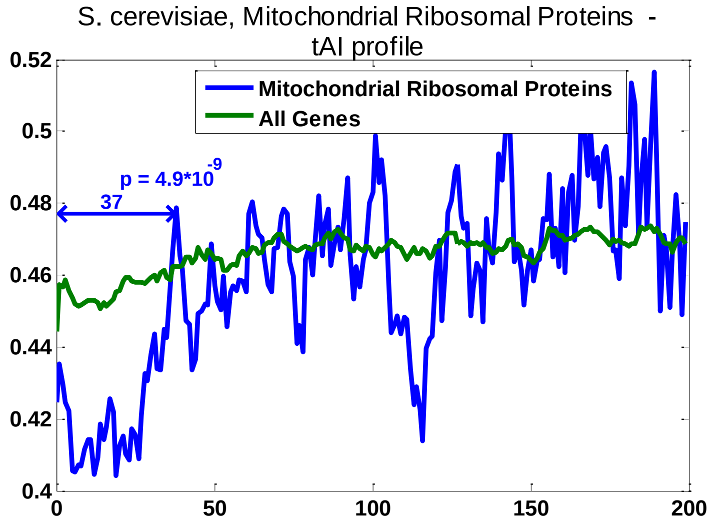

Supplementary Figure S8. appears in a different file.

Supplementary Figure S9. mRNA folding robustness profiles (number of mutations with identical folding energy) in *S. cerevisiae* for different ranges of folding energy (control for folding energy). In all ranges there is a region of increased robustness at the beginning of the genes (p-values appear in the figure).

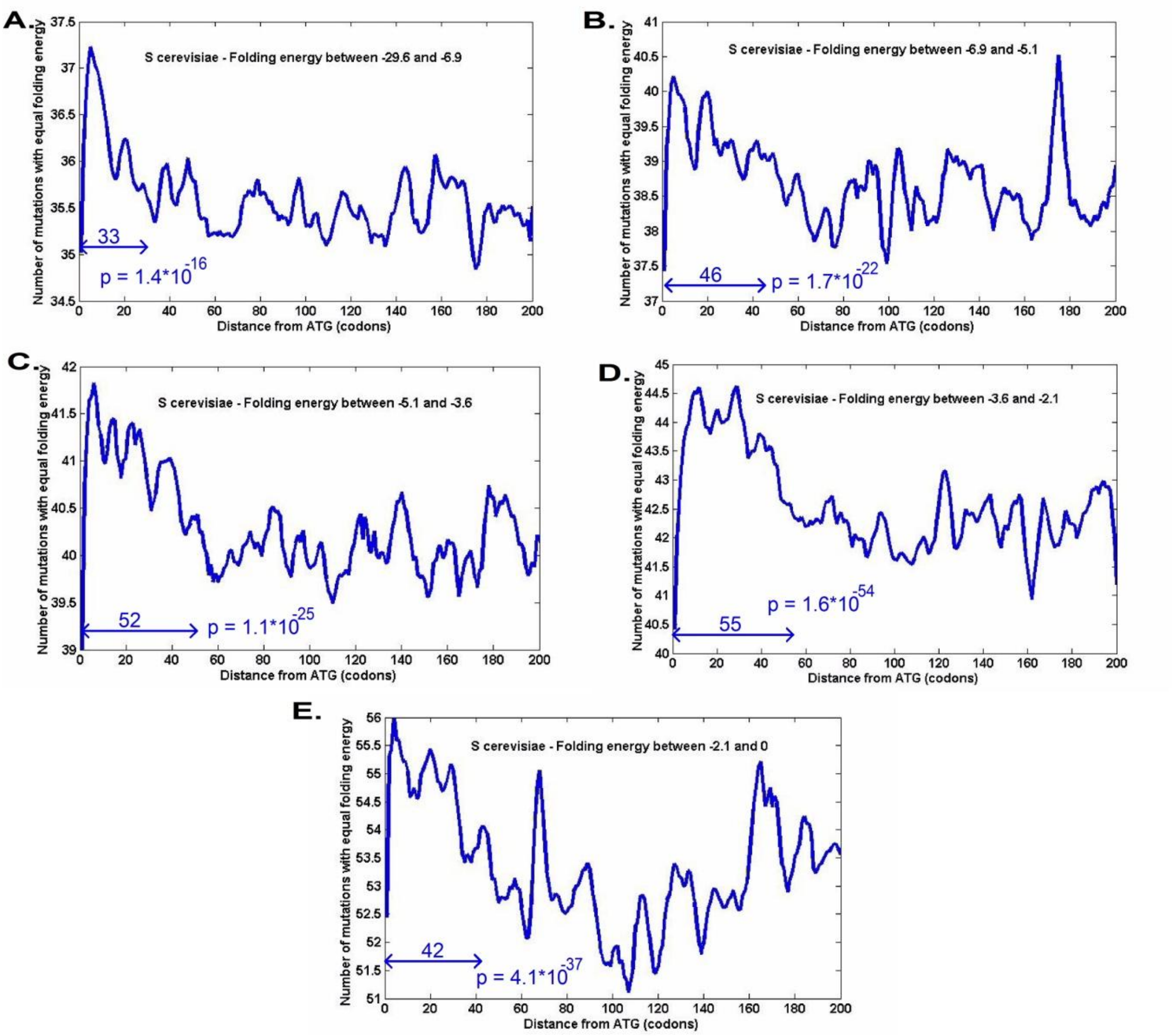

Supplementary Figure S10. mRNA folding robustness profiles (number of mutations with identical folding energy) in *E. coli* for different ranges of folding energy (control for folding energy). In all ranges the robustness at the beginning of the coding sequence (ramp) is significantly larger than the robustness of the rest of the codons. The ramps and p-values are marked in the Figure.

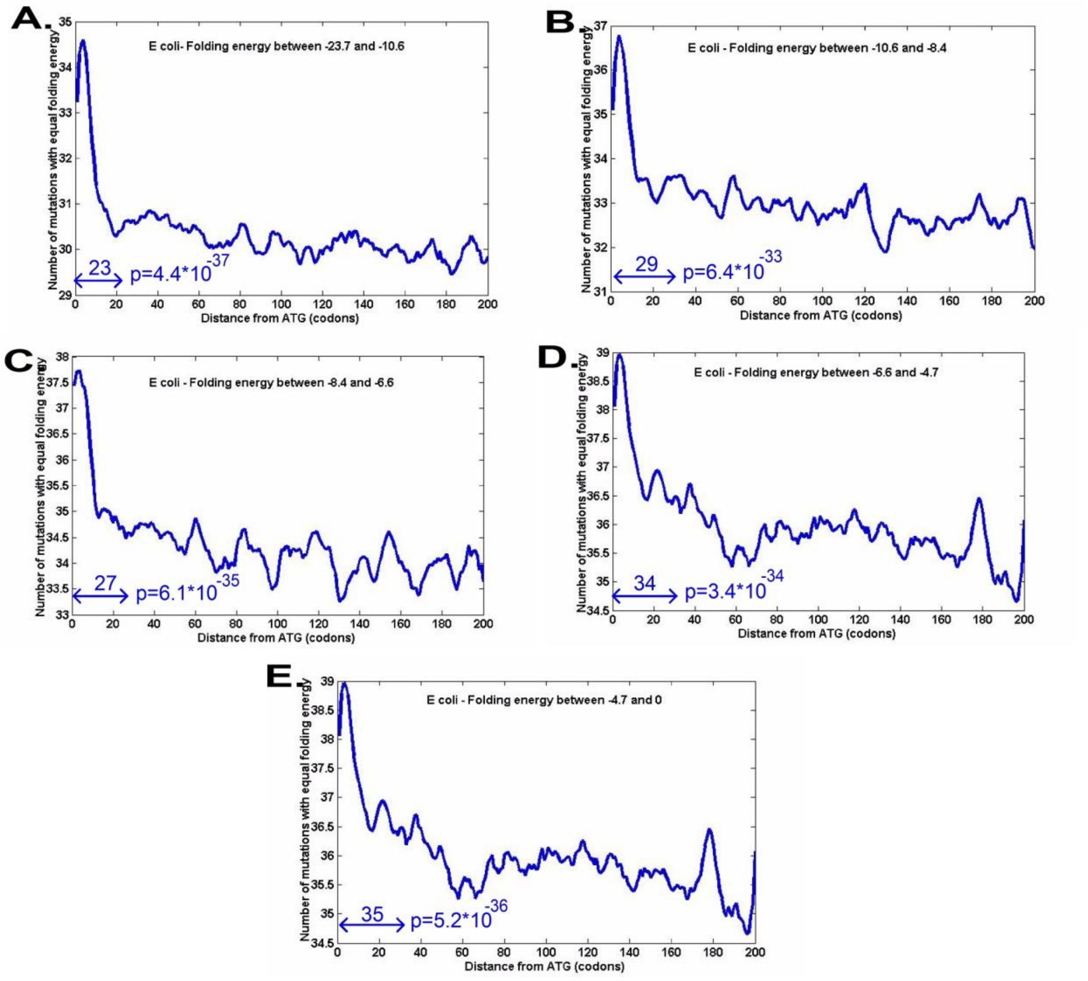

Supplementary Figure S11. mRNA folding robustness profiles (mean difference in folding energy between the original sequence and its mutants) in *S. cerevisiae* for different ranges of folding energy (control for folding energy). In all ranges the robustness of the first codons (the ramp) is significantly larger than the robustness of the rest of the codons. The ramps and p-values are marked in the figure.

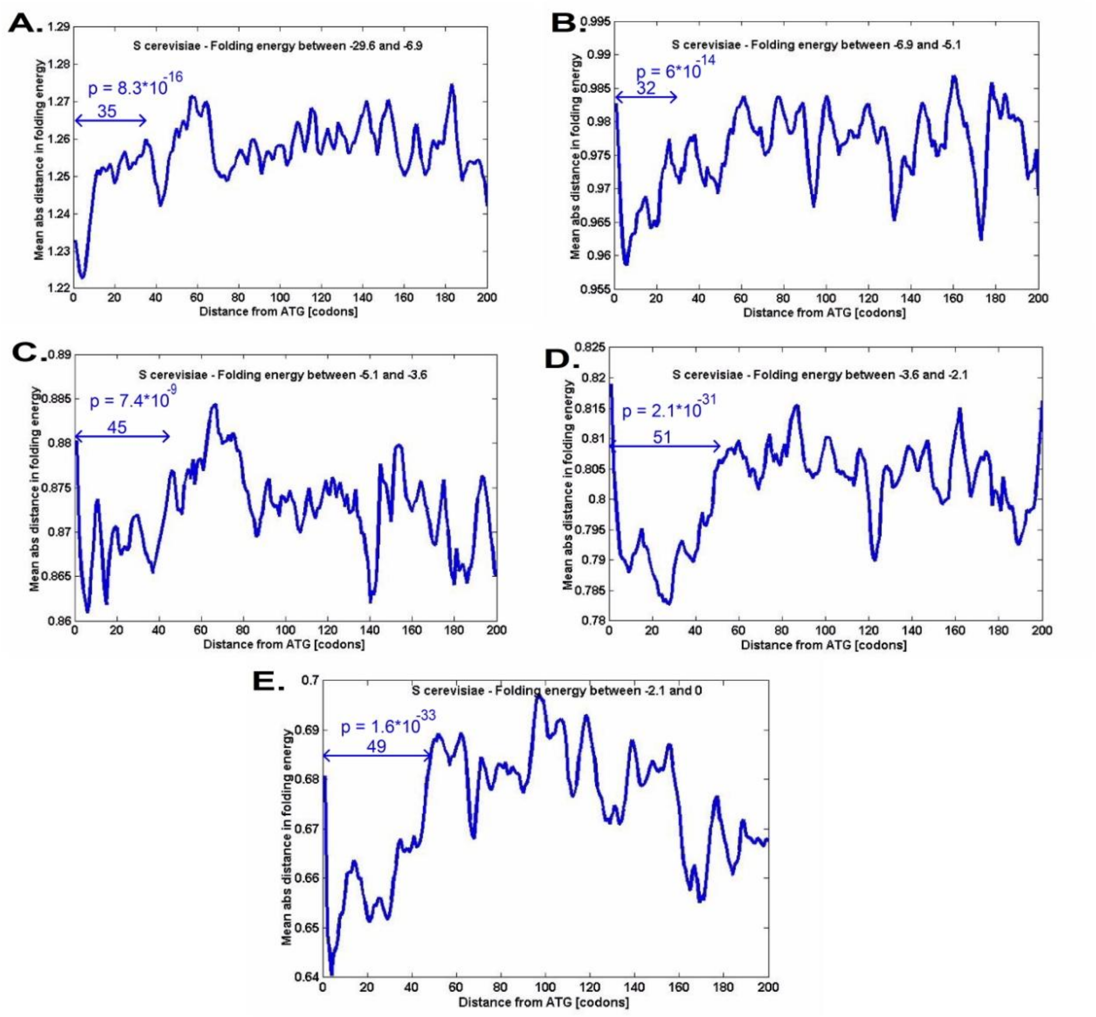

Supplementary Figure S12. mRNA folding robustness profiles (mean difference in folding energy between the original sequence and its mutants) in *E. coli* for different ranges of folding energy (control for folding energy). In four ranges the robustness of the first codons (the ramp) is significantly larger than the robustness of the rest of the codons. The ramps and the p-values are marked in the figure.

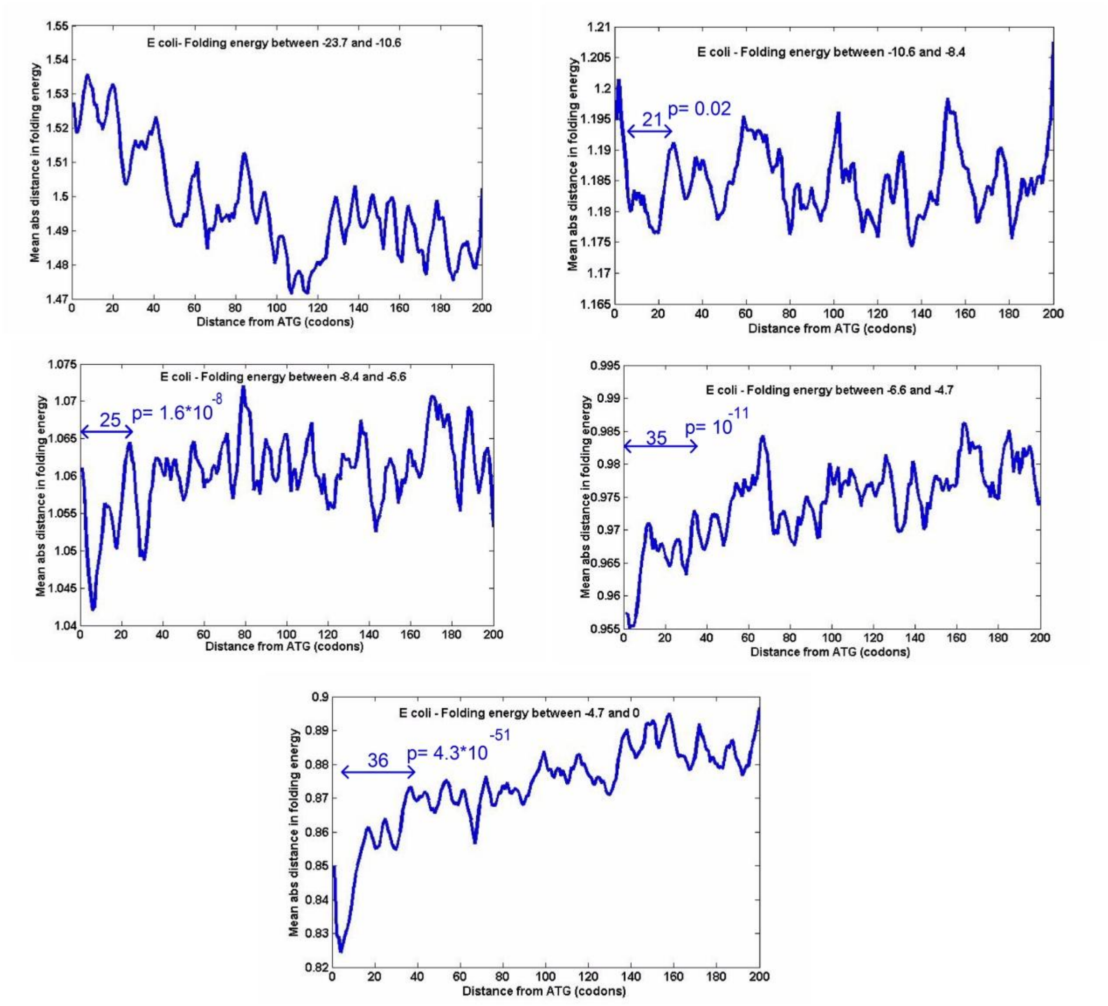

Supplementary Figure S13. mRNA folding robustness profiles (number of changes in base-pairs) in *E. coli* for different ranges of folding energy (control for folding energy). In all ranges the robustness of the first codons (the ramp) is significantly larger than the robustness of the rest of the codons. The length of the ramp and the corresponding p-values appear in the figures.

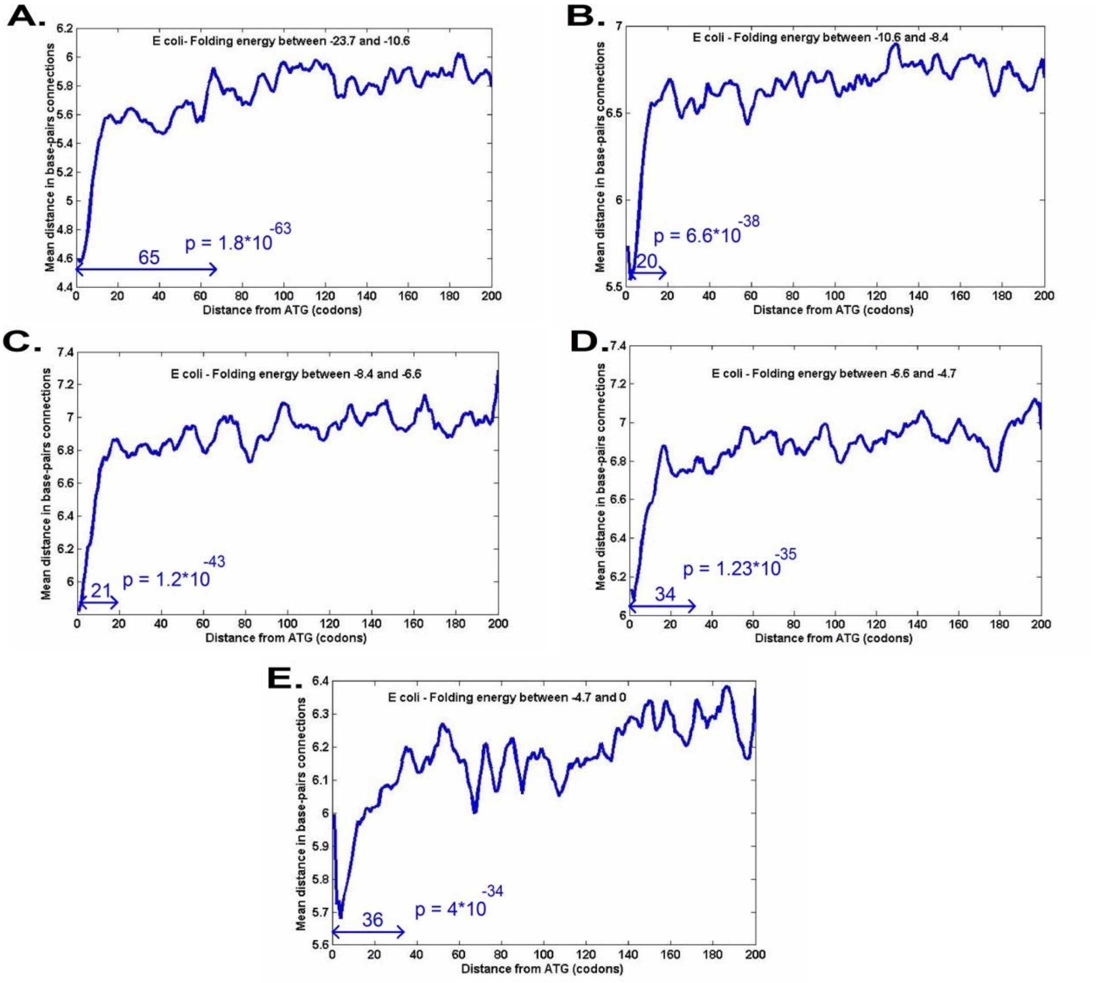

Supplementary Figure S14. Folding energy robustness profile in *S. cerevisiae* - comparison of each position to all other positions. We considered the three measures of robustness: Mean absolute difference in folding energy between the original sequence and all the mutation (A.), Mean difference in base pairs between the original sequence and all the mutation (B.), Number of mutations that preserve the folding energy (C.). Length of the ramps and p-values appear in the figures.

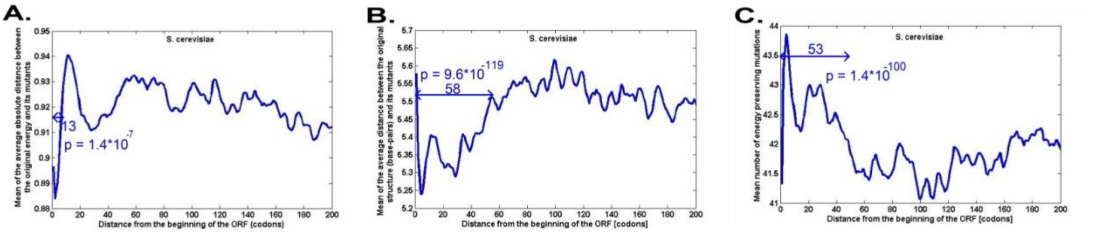

Supplementary Figure S15. Folding energy robustness profile in E. coli - comparison of each position to all other positions. We considered the three measures of robustness: Mean absolute difference in folding energy between the original sequence and all the mutation (A.), Mean difference in base pairs between the original sequence and all the mutation (B.), Number of mutations that preserve the folding energy (C.). The length of the ramps and corresponding p-values appear in the figure.

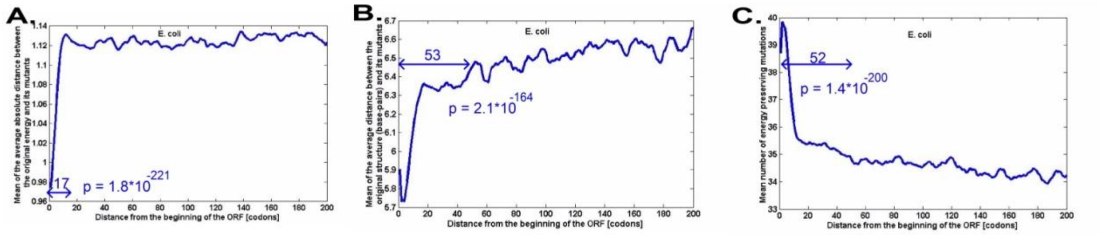

Supplementary Figure S16. Folding energy robustness profile in *S. cerevisiae* - comparison of each position to the corresponding position in a randomized version of the genome (control for amino acid bias). We considered the three measures of robustness: Mean absolute difference in folding energy between the original sequence and all the mutation (A.), Mean difference in base pairs between the original sequence and all the mutation (B.), Number of mutations that preserve the folding energy (C.). The robustness of the first 50 codons is significantly higher for the real sequences. The ramps and corresponding p-values are marked in the figure.

To control for folding energy in case A., for position where the mean folding energy is higher for the real sequences vs. the randomized sequences, we considered the *k* (smallest possible *k*) windows with the lowest folding energy for the real sequences and compared it to the k randomized sequences with the highest folding energy. We chose the smallest k such that the two means of folding energy were similar (similar procedure was performed for position where the mean folding energy is lower for the real sequences *vs.* the randomized sequences).

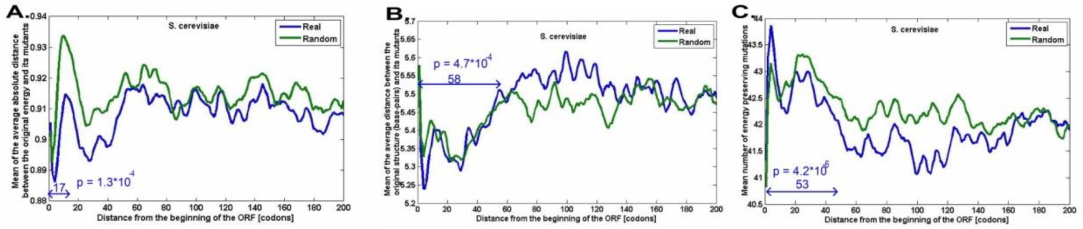

Supplementary Figure S17. Folding energy robustness profile in E. coli - comparison of each position to the corresponding position in a randomized version of the genome (control for amino acid bias). We considered the three measures of robustness: Mean absolute difference in folding energy between the original sequence and all the mutation (A.), Mean difference in base pairs between the original sequence and all the mutation (B.), Number of mutations that preserve the folding energy (C.). The robustness of the first codons is significantly higher for the real sequences. The ramps and corresponding p-values appear in the figure.

To control for folding energy in case A., for position where the mean folding energy is higher for the real sequences vs. the randomized sequences, we considered the *k* (smallest possible *k*) windows with the lowest folding energy for the real sequences and compared it to the k randomized sequences with the highest folding energy. We chose the smallest k such that the two means of folding energy were similar (similar procedure was performed for position where the mean folding energy is lower for the real sequences *vs.* the randomized sequences).

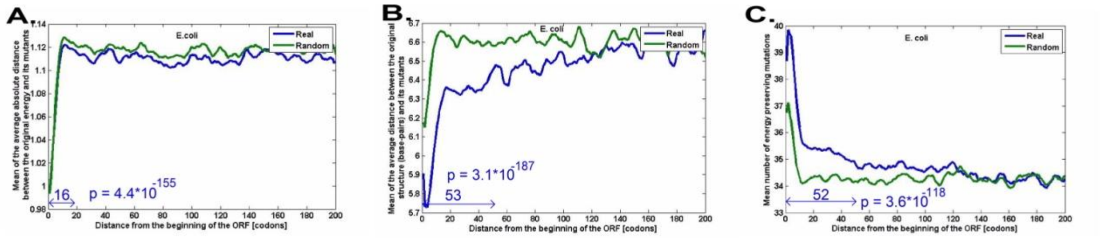

Supplementary Figure S18. tAI robustness profiles (number of mutations with identical tAI) in *S. cerevisiae* for different ranges of tAI (control for tAI).In all ranges the robustness of the first codons (the ramp) is significantly larger than the robustness of the rest of the codons. The ramps and p-values are marked in the figure.

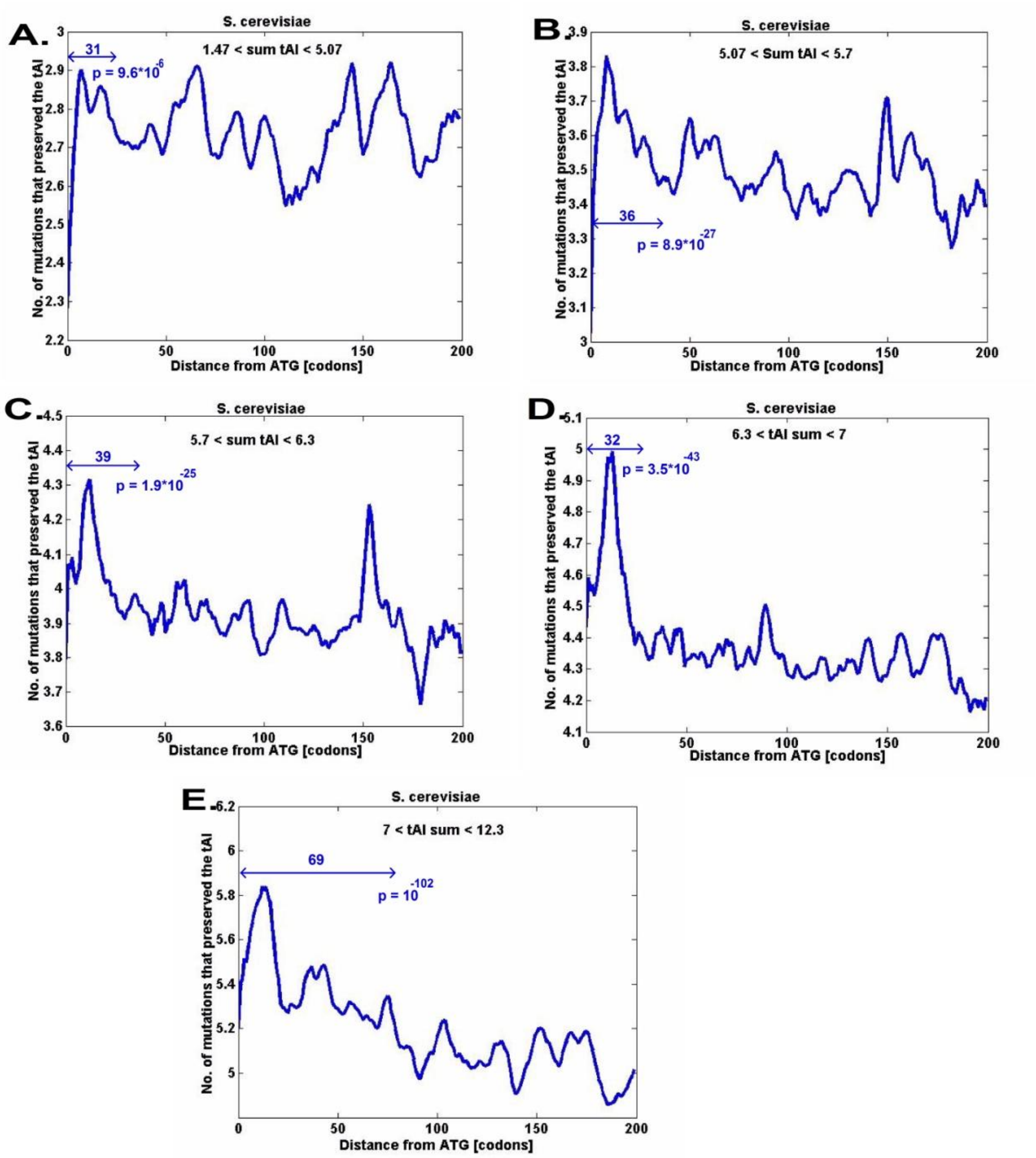

Supplementary Figure S19. tAI robustness profiles (number of mutations with identical *tAI*) in *E. coli* for different ranges of tAI (control for tAI).In all ranges the robustness of the first codons (the 'ramp') is significantly larger than the robustness of the rest of the codons. The ramps and p-values appear in the figures.

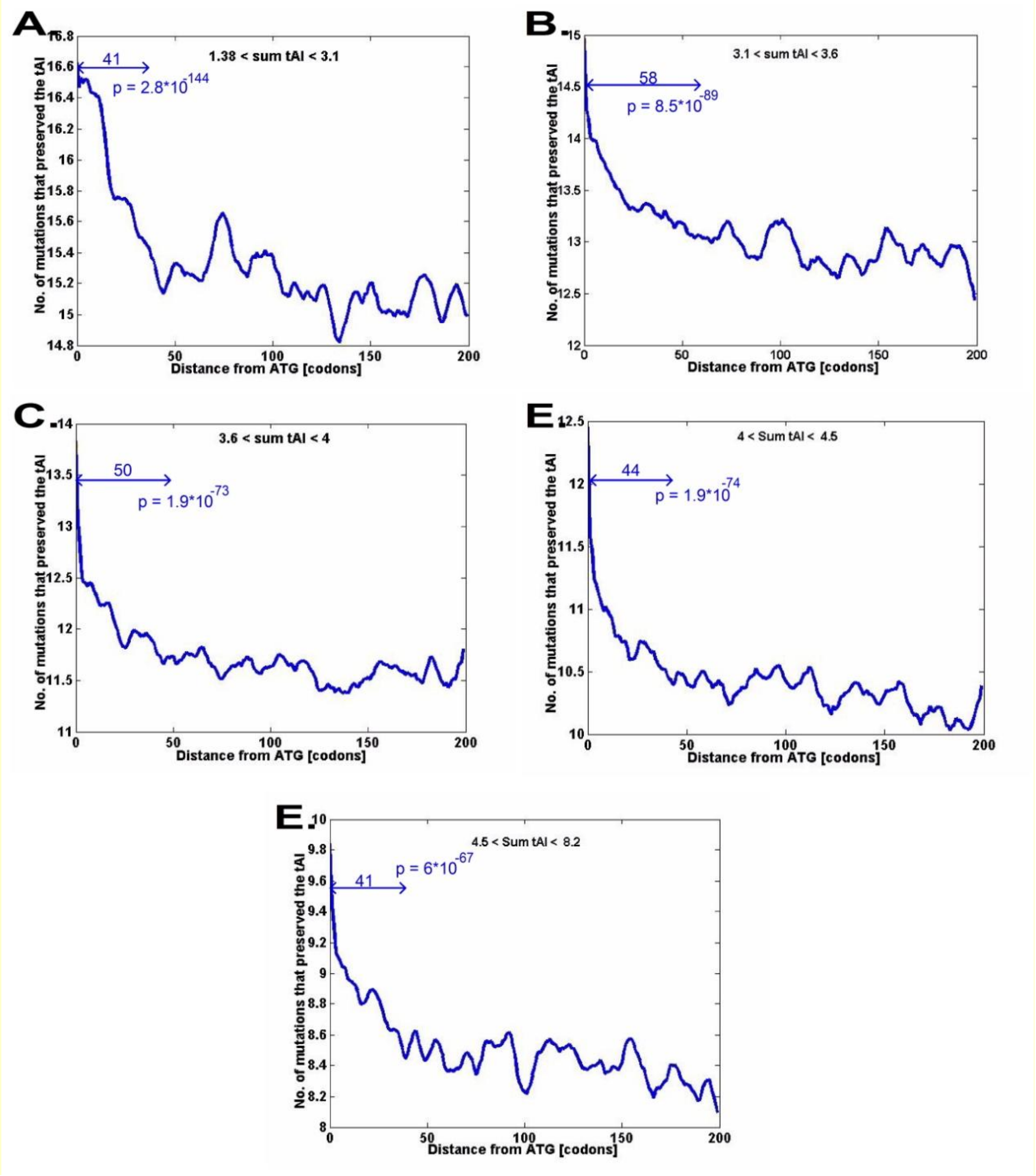

Supplementary Figure S20. tAI robustness profiles (number of mutations with identical tAI) in *E. coli.* The length of the robust region (the 'ramp') and the corresponding p-value appear in the figure.

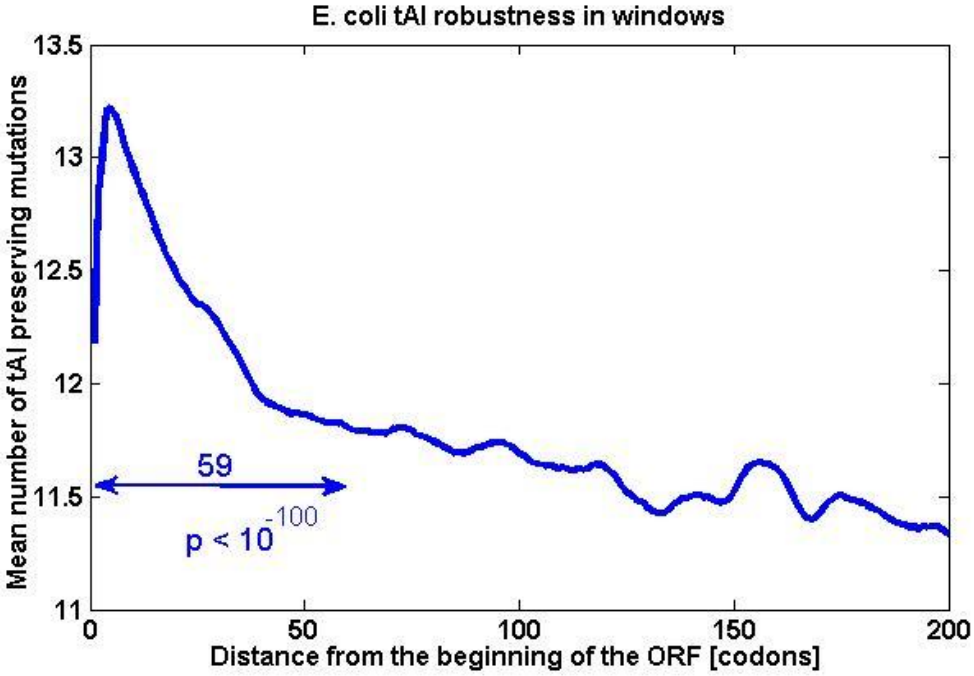

Supplementary Figure S21. tAI robustness profiles - comparison of each position to the corresponding position in a randomized version of the genome (control for amino acid bias) in *S. cerevisiae.* The robustness of the first codons is significantly higher for the real sequences. The ramp and the corresponding p-value appear in the figure.

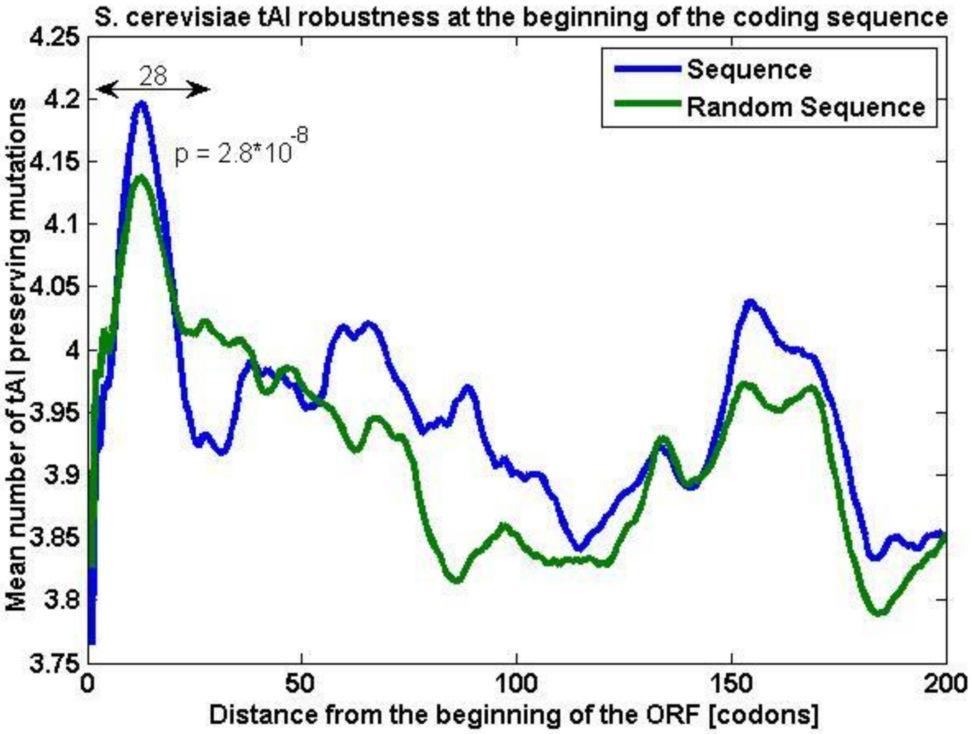

Supplementary Figure S22. tAI robustness profiles - comparison of each position to the corresponding position in a randomized version of the genome (control for amino acid bias) in *E. coli.* The length of the ramp and a corresponding p-value appear in the figure.

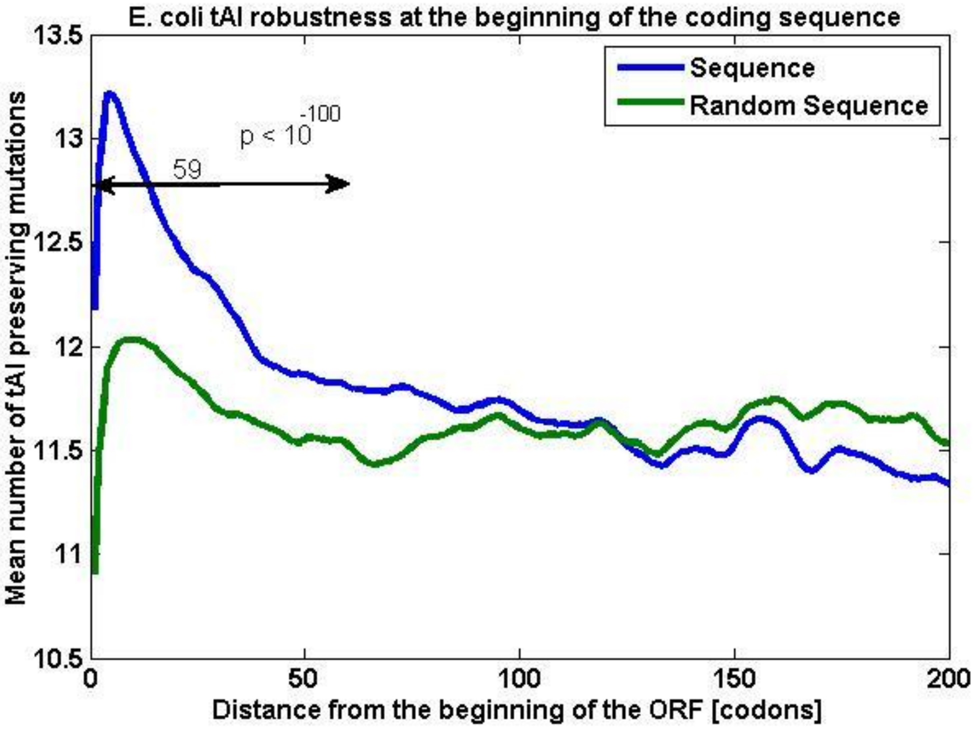

Supplementary Figure S23. Charge robustness profiles (number of mutations that do not change the charge) in *S. cerevisiae* for different ranges of charge (control for charge). In 4 out of 5 ranges the robustness of the first codons (ramp) is significantly larger than the robustness of the rest of the codons. The ramps and the corresponding p-values are marked in the figures.

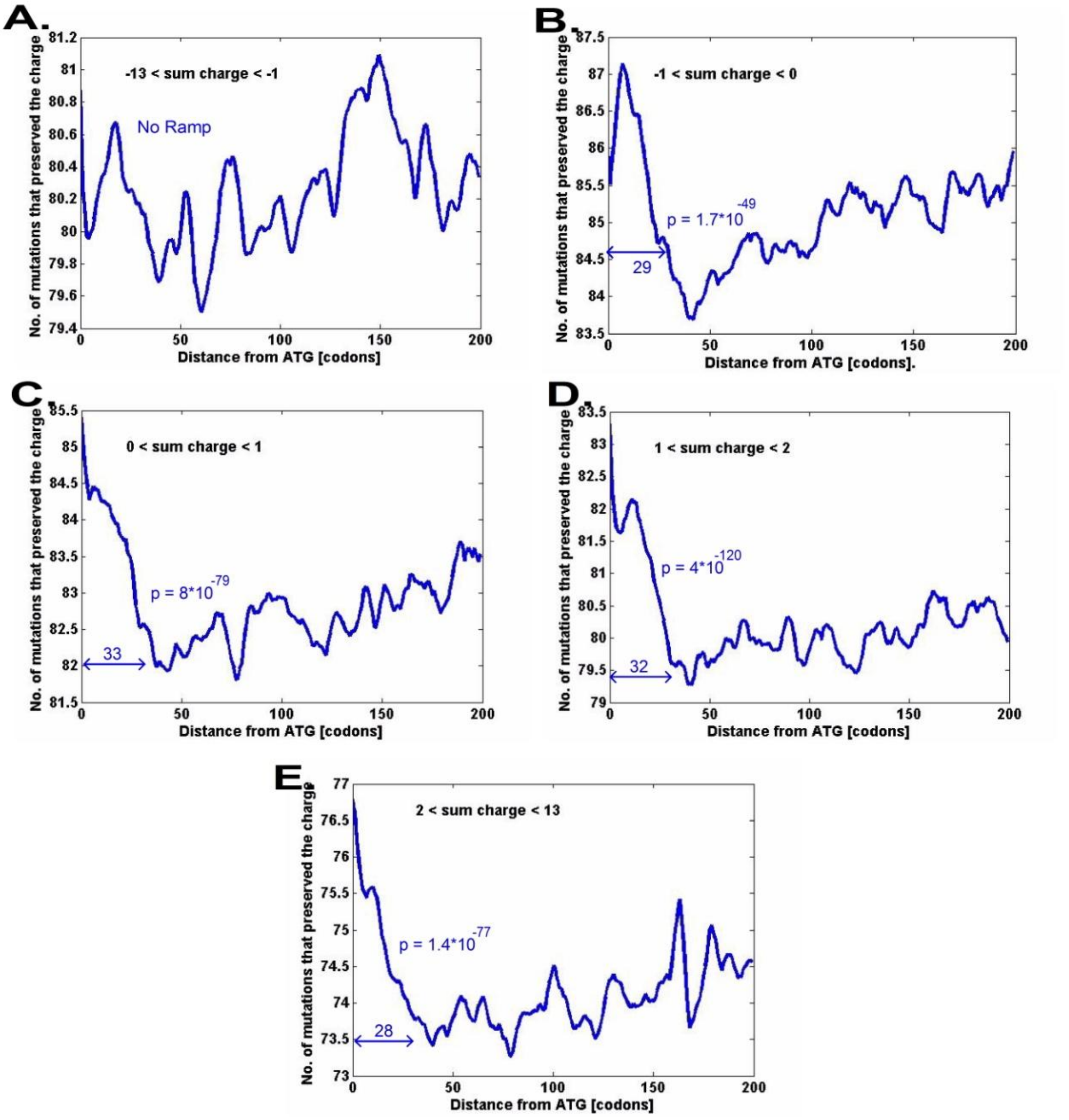

Supplementary Figure S24. Charge robustness profiles (number of mutations that do not change the charge) in *E. coli* for different ranges of charge (control for charge). In all ranges the robustness of the first codons is significantly larger than the robustness of the rest of the codons. The ramps and corresponding p-values appear in the figures.

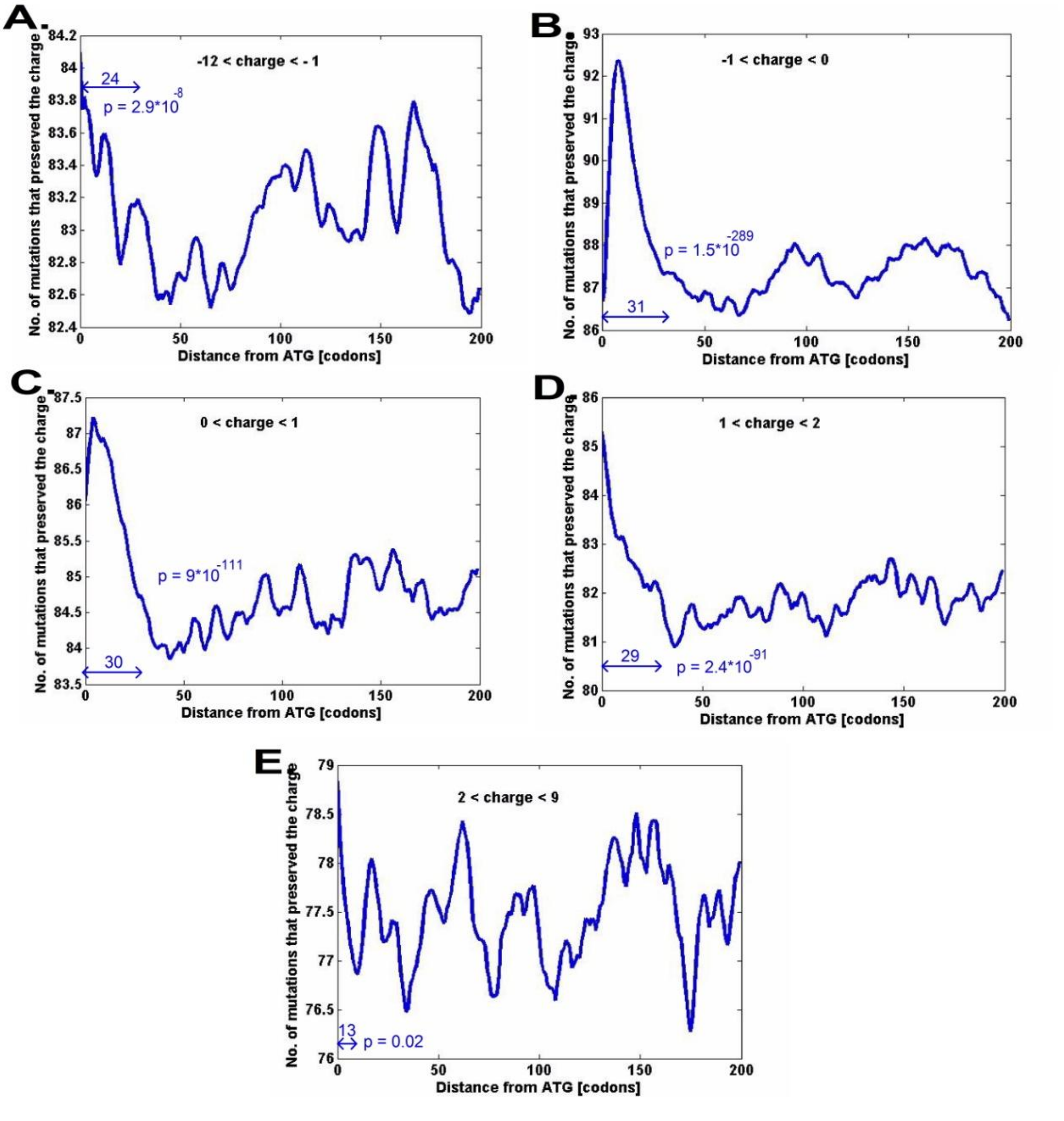

Supplementary Figure S25. Charge robustness profiles (number of mutations that do not change the charge) in *E. coli*. The length of the region with increased robustness at the beginning and the corresponding p-value appear in the figure.

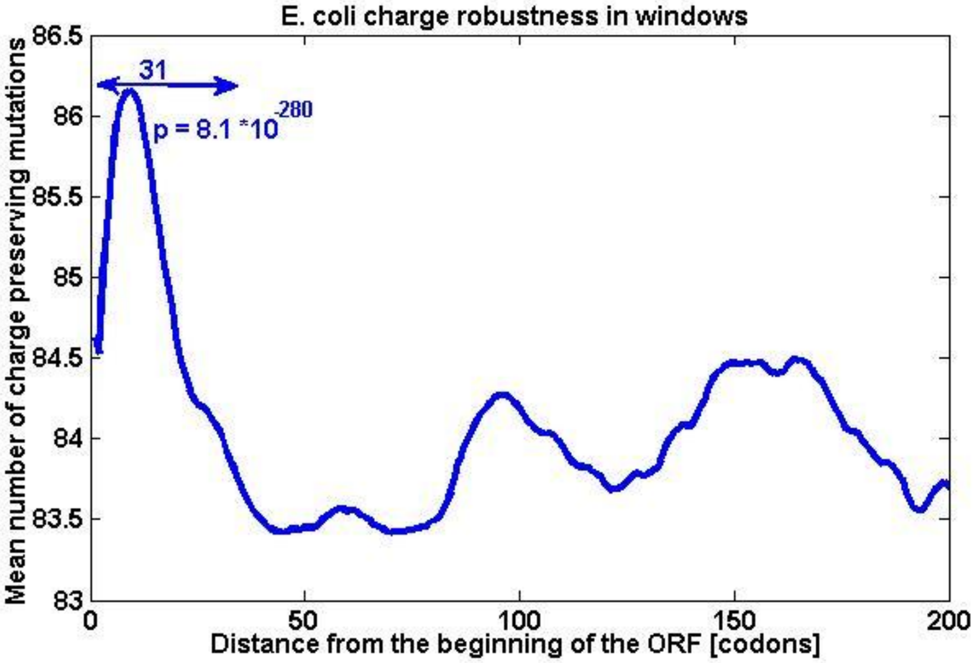

Supplementary Figure S26: mRNA folding robustness profiles (A. The mean absolute difference in folding energy; B. The mean number of changes in base-pairs; C. The mean number of mutations preserving the folding energy) in *S. cerevisiae* when giving different weights to different mutations. The green graph corresponds to giving transitions twice the weight of transversions and the blue graph is the original one (for comparison); the two graphs are very similar (correlation larger than 0.99 in all the three cases).
In all the cases the robustness of the first codons is significantly larger than the robustness of the rest of the codons. The ramps and corresponding p-values are marked in the figure.

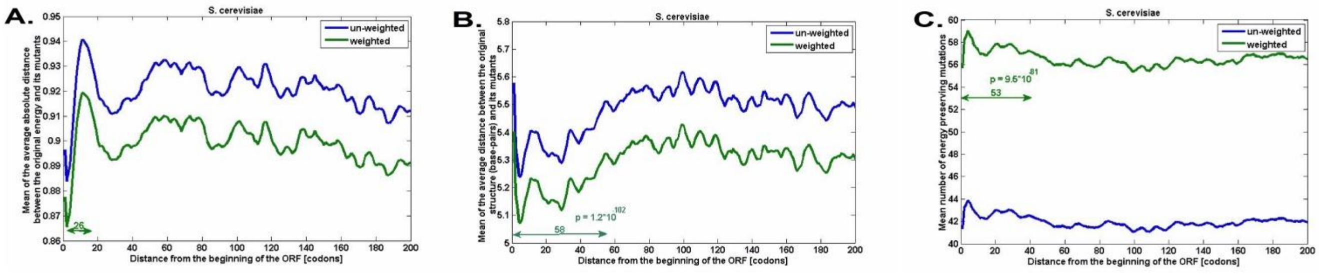

Supplementary Figure S27: mRNA folding robustness profiles (A. The mean absolute difference in folding energy; B. The mean number of changes in base-pairs; C. The mean number of mutations preserving the folding energy) in *E. coli* when giving different weights to different mutations. The green graph corresponds to giving transitions twice the weight of transversions and the blue graph is the original one (for comparison); the two graphs are very similar (correlation larger than 0.99 in all the three cases).
In all the cases the robustness of the first codons is significantly larger than the robustness of the rest of the codons. The ramps and corresponding p-values are marked in the figure.

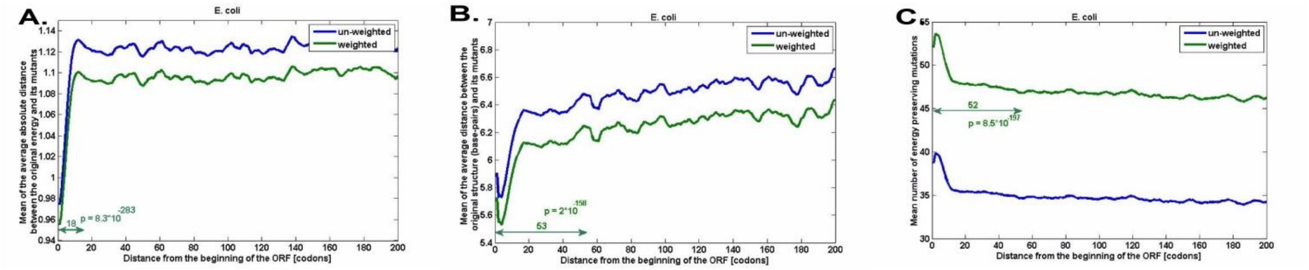

Supplementary Figure S28: tAI robustness profiles (The mean number of mutations preserving the tAI) in *S. cerevisiae* when giving different weights to different mutations. The green graph corresponds to giving transitions twice the weight of transversions and the blue graph is the original one (for comparison); the two graphs are very similar (correlation larger than 0.99).
The robustness of the first codons is significantly larger than the robustness of the rest of the codons. The ramp and corresponding p-value are marked in the figure.

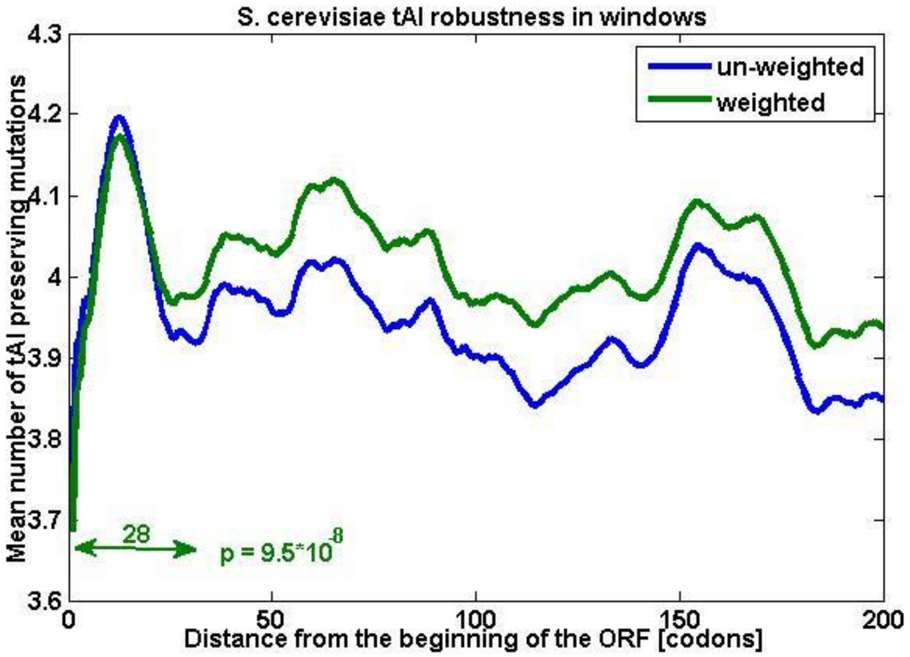

Supplementary Figure S29: *tAI* robustness profiles (The mean number of mutations preserving the tAI) in *E. coli* when giving different weights to different mutations. The green graph corresponds to giving transitions twice the weight of transversions and the blue graph is the original one (for comparison); the two graphs are very similar (correlation larger than 0.99).

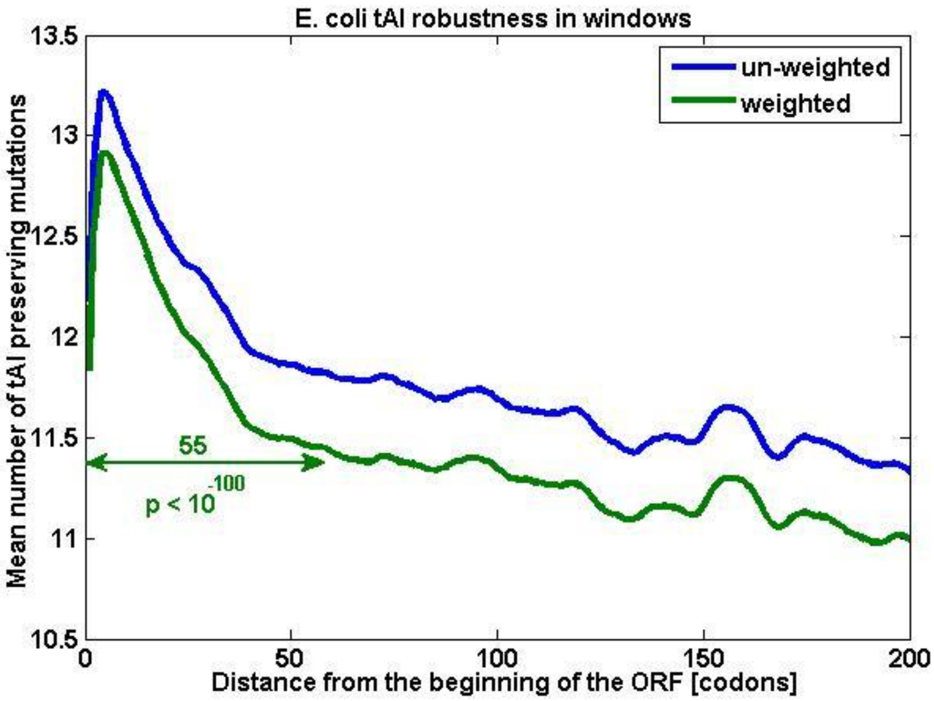

Supplementary Figure S30: charge robustness profiles (The mean number of mutations preserving the charge) in *S. cerevisiae* when giving different weights to different mutations. The green graph corresponds to giving transitions twice the weight of transversions and giving the first and the last positions of the codons a ten times higher weight; the blue graph is the original one (for comparison); the two graphs are very similar (correlation larger than 0.99).
The robustness of the first codons is significantly larger than the robustness of the rest of the codons. The ramp and corresponding p-value are marked in the figure.

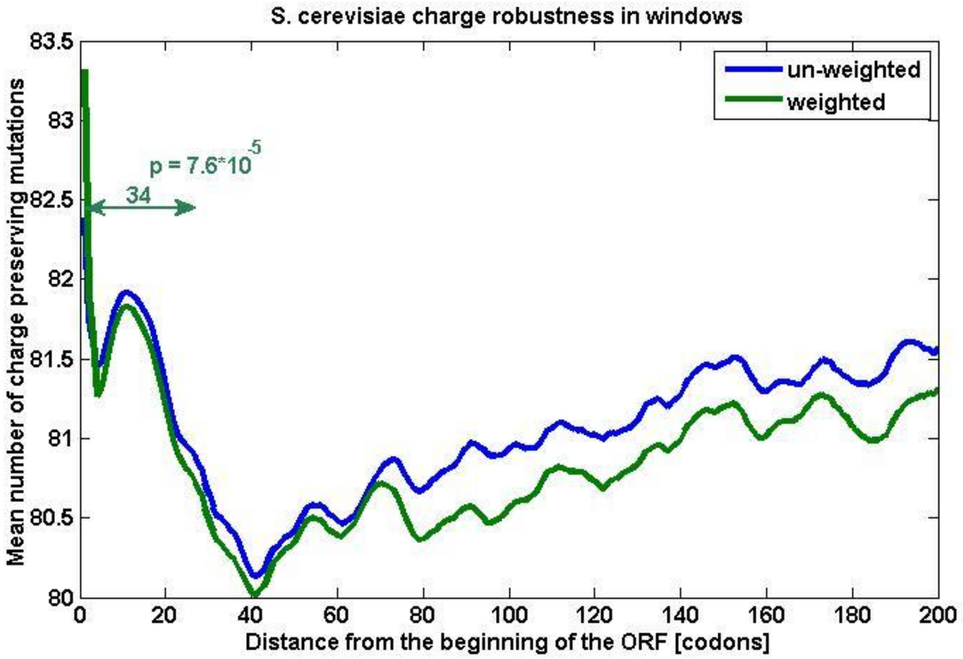

Supplementary Figure S31: charge robustness profiles (The mean number of mutations preserving the charge) in *E. coli* when giving different weights to different mutations. The green graph corresponds to giving transitions twice the weight of transversions and giving the first and the last positions of the codons a ten times higher weight; the blue graph is the original one (for comparison); the two graphs are very similar (correlation larger than 0.99).
The robustness of the first codons is significantly larger than the robustness of the rest of the codons. The ramp and corresponding p-value are marked in the figure.

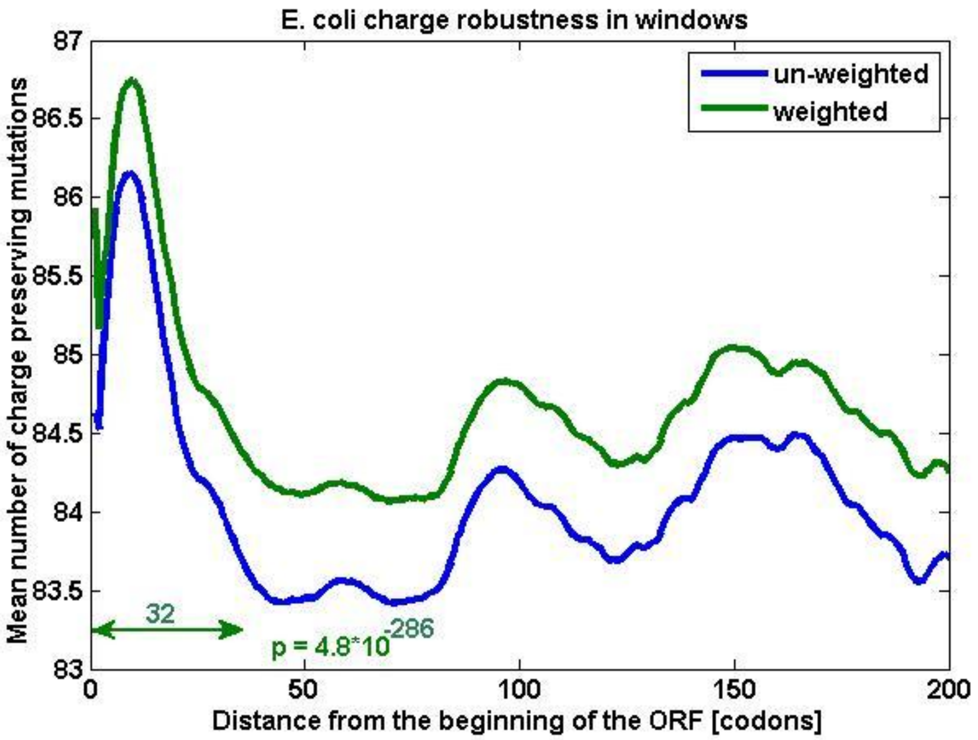

Supplementary Figure S32. Dot plots of the ribosome density profile *vs.* the genomic profiles in *S. cerevisiae*. A. Genomic ribosomal density *vs.* genomic charge profile. B. Genomic ribosomal density *vs.* genomic folding energy. C. Genomic ribosomal density *vs.* genomic 1/tAI profile. D. Genomic ribosomal density *vs.* genomic predicted ribosomal density by a regression. D. Genomic ribosomal density *vs.* genomic predicted ribosomal density by the TASEP model. The a bit unusual shape of the profile of the ribosomal density *vs.* the folding energy (B.) is due to the fact that at the beginning of the coding sequence the ribosomal density is high while there is a region with very weak folding that is followed a region very strong folding.

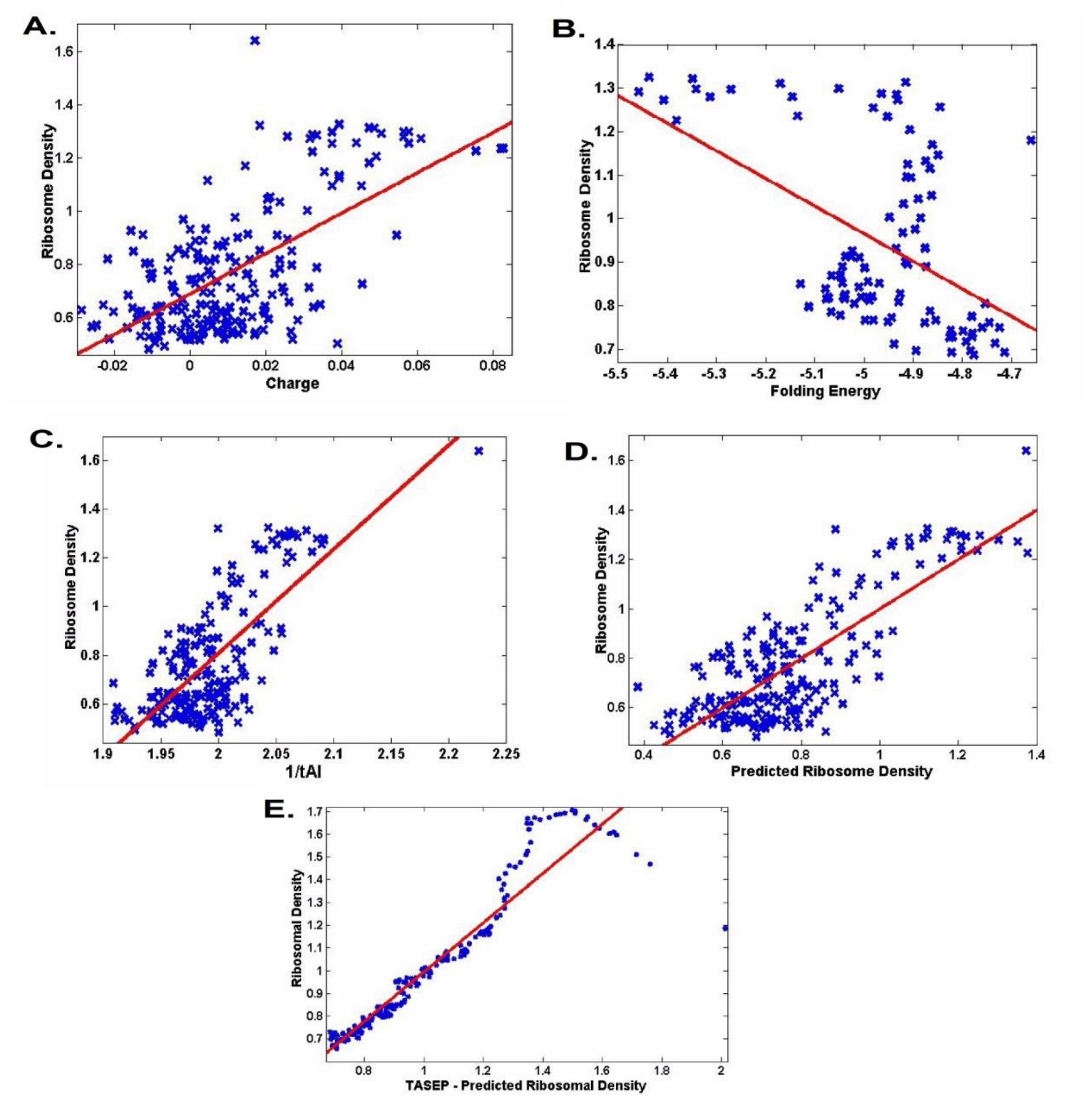

Supplementary Figure S33. The genomic profile of charge at the end of genes in S. cerevisiae.

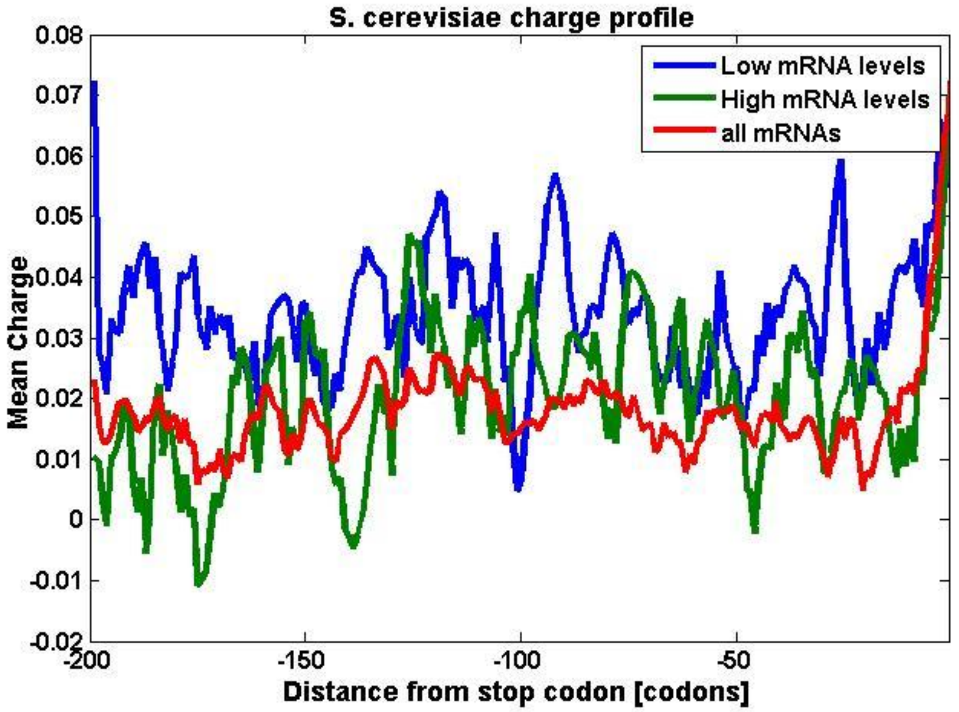

Supplementary Figure S34. The genomic profile of tAI at the end of genes in *S. cerevisiae*.

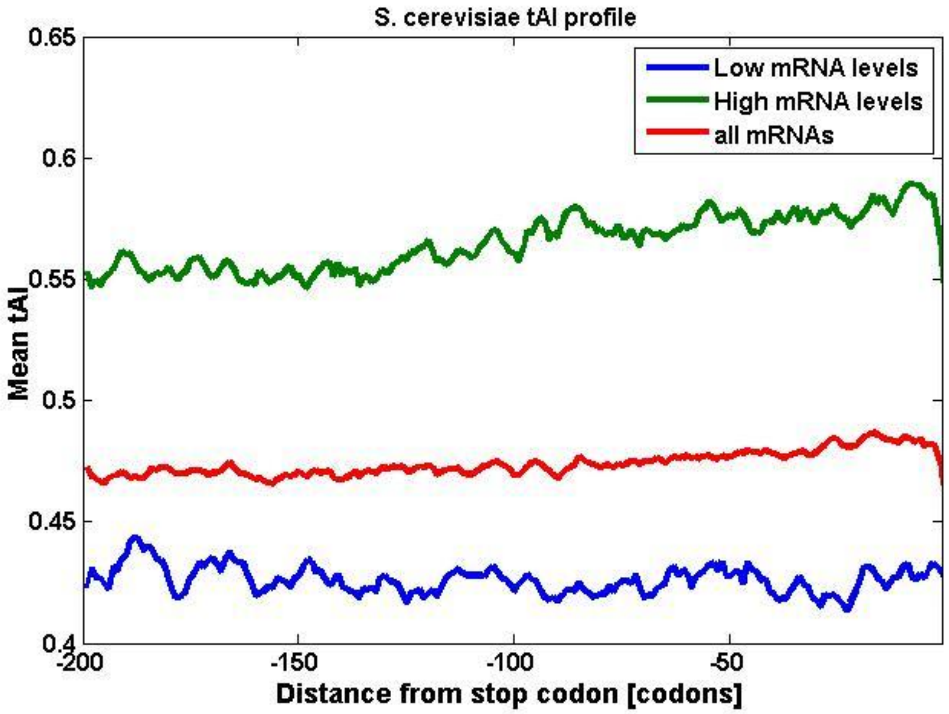

Supplementary Figure S35. The genomic profile of folding energy at the end of genes in S. cerevisiae.

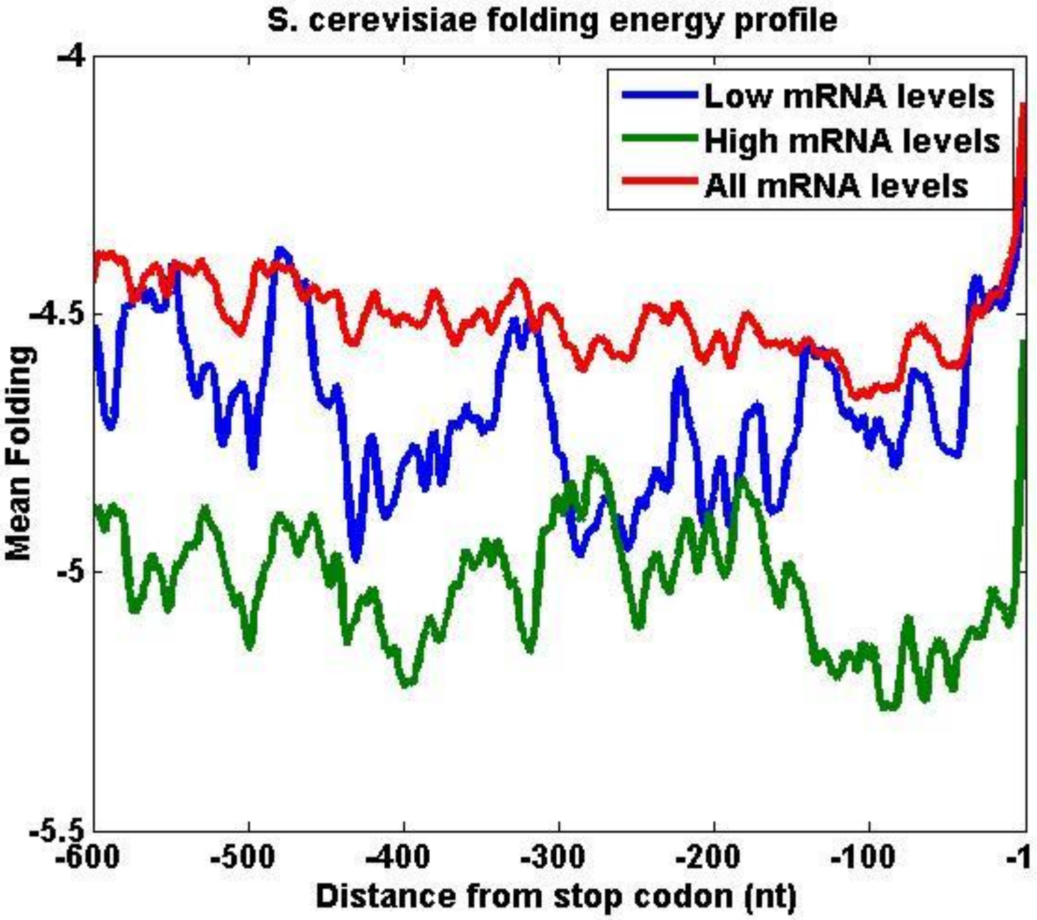

Supplementary Figure S36. Genomic profile of folding energy robustness at the end of genes in S. cerevisiae (the mean absolute distance between the robustness of the original sequences and the randomized ones).

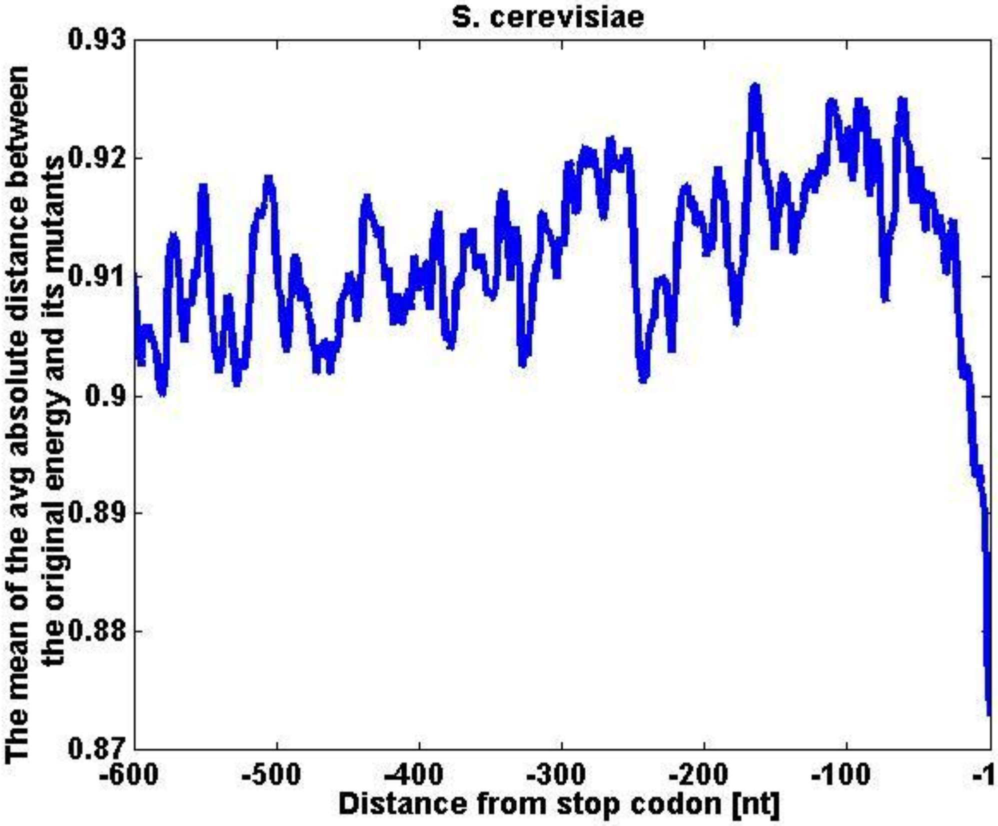

Supplementary Figure S37. mRNA folding robustness profiles (Mean difference in base pairs between the original sequence and all the mutation) in S. cerevisiae for different ranges of folding energy (control for folding energy).

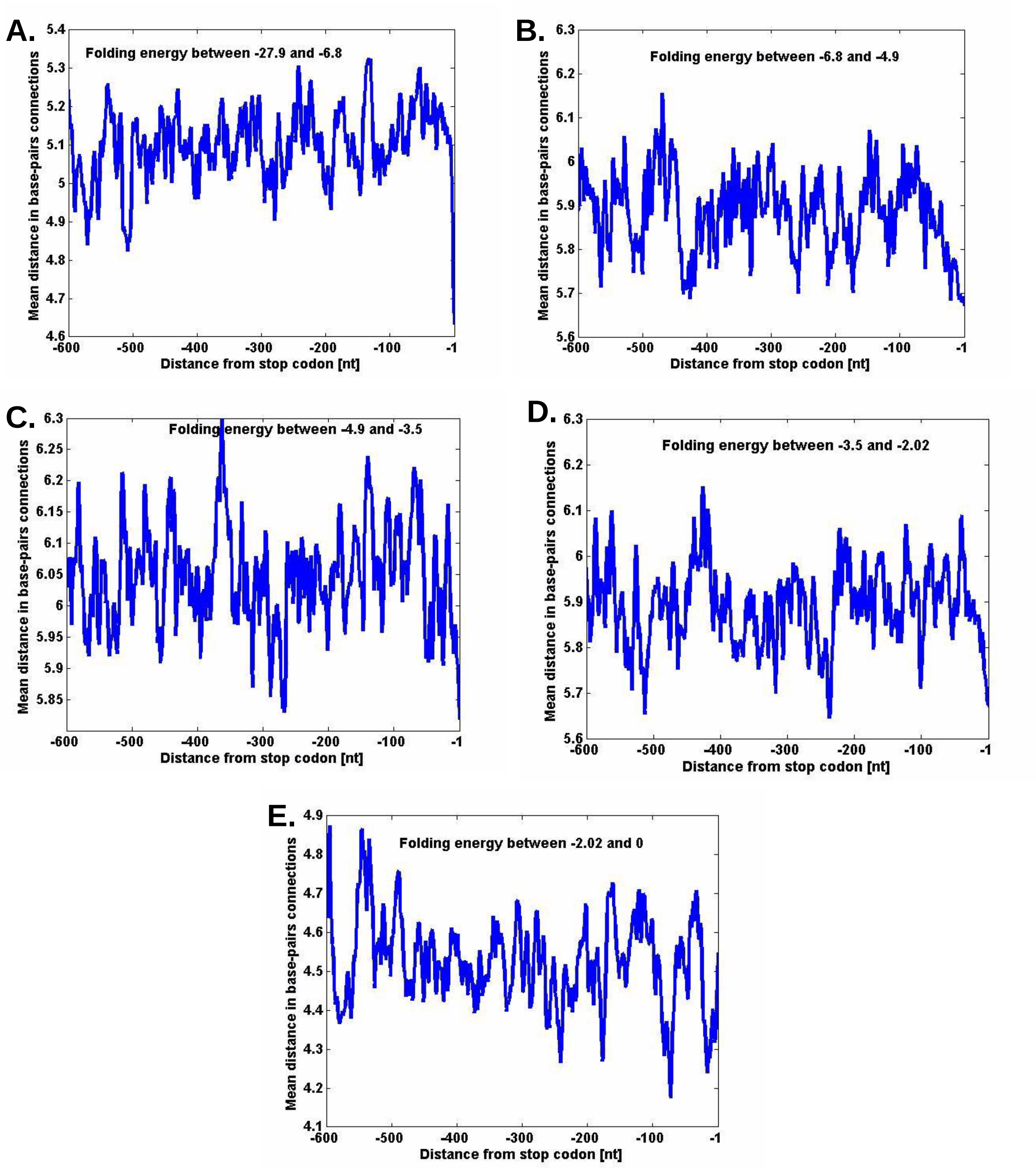

Supplementary Figure S38. The genomic profile of tAI robustness (randomized profile vs. real one) in S. cerevisiae at the end of genes. There is decrease in tAI robustness at the end of genes.

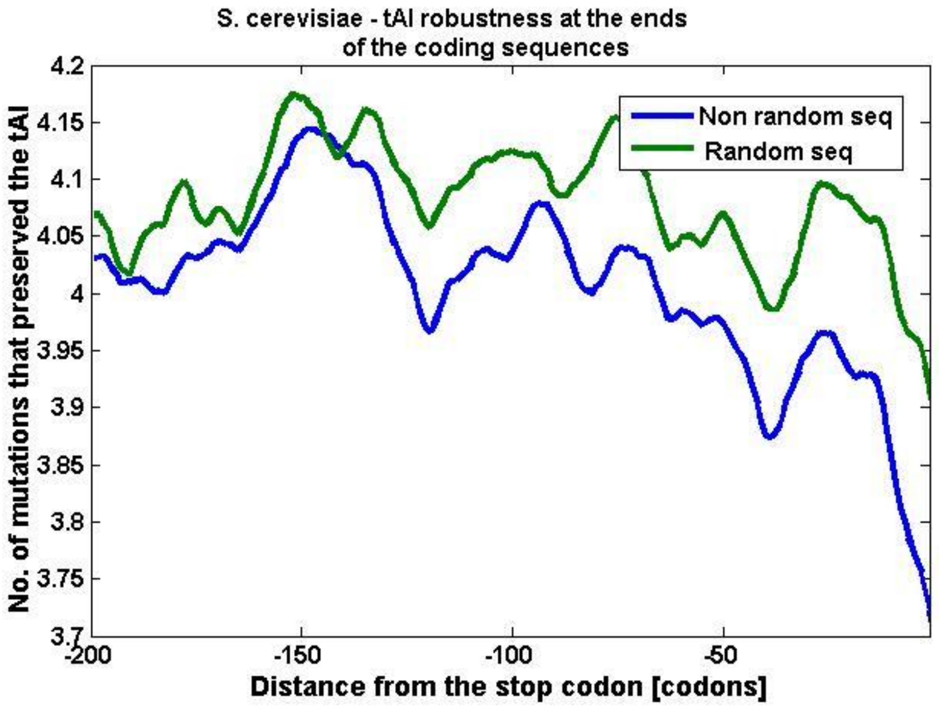

Supplementary Figure S39. The genomic profile of charge robustness (randomized profile vs. real one) in S. cerevisiae at the end of genes. There is decrease in charge robustness at the end of genes.

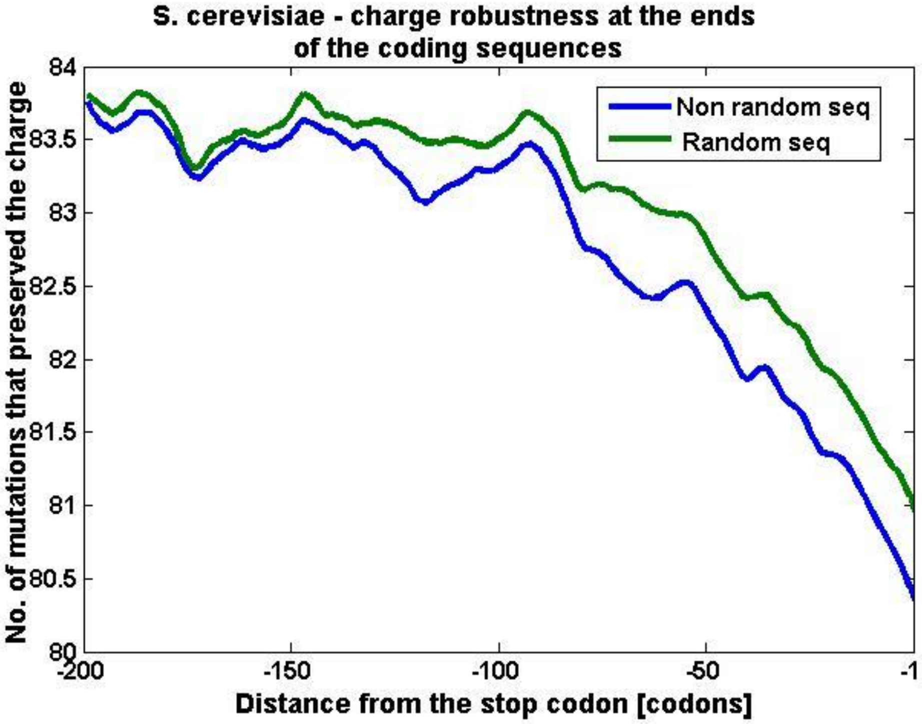

Supplementary figure S40. Genomic profile of pairs of identical slow codons in *E. coli* (blue) *vs.* the *randomized* genomic profile of pairs of identical slow codons in *E. coli* (control for amino acid bias and codon bias). There is a 'ramp' of slow pairs of identical codons at the beginning of length 15 (p = 0.0091) it is higher than the randomized profile but it is not significant (p = 0.1) .

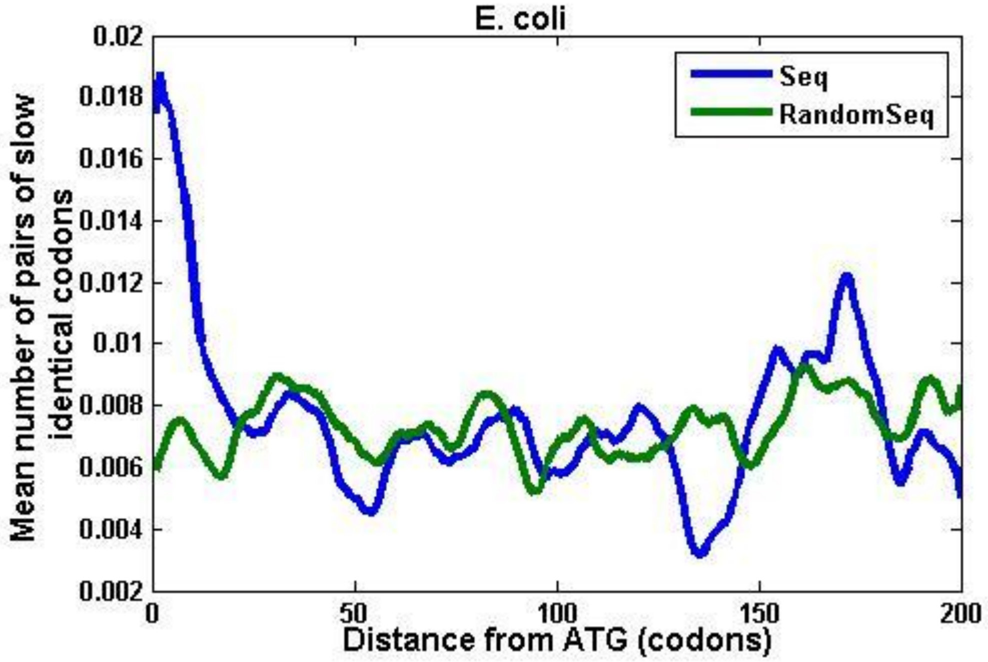

Supplementary figure S41. Genomic profile of pairs of identical slow codons in *E. coli* (blue) for highly expressed genes *vs.* the *randomized* genomic profile of pairs of identical slow codons in *E. coli* for highly expressed genes (control for amino acid bias and codon bias). The ramp is not significant under this control for highly expressed genes.

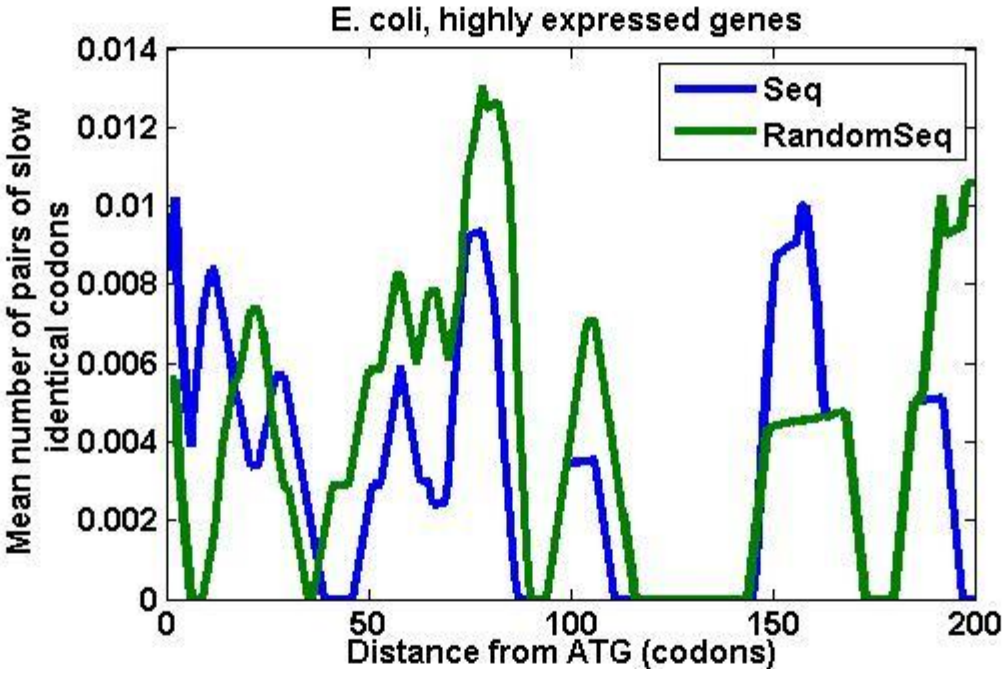

Supplementary figure S42. Genomic profile of pairs of identical slow codons in *E. coli* (blue) for lowly expressed genes *vs.* the *randomized* genomic profile of pairs of identical slow codons in *E. coli* for lowly expressed genes (control for amino acid bias and codon bias). The ramp is not significant under this control for lowly expressed genes.

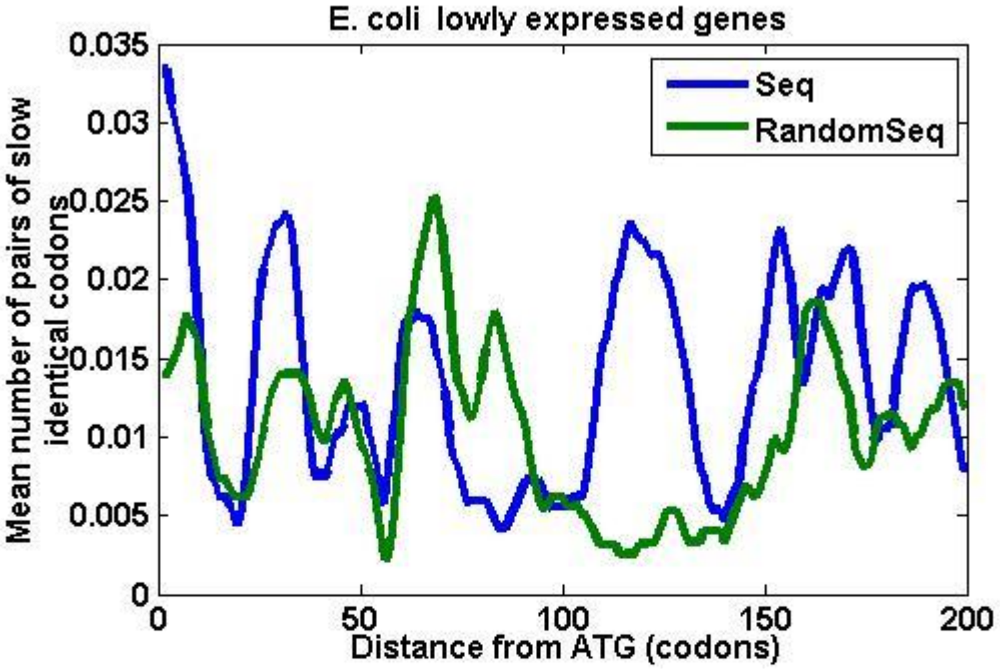

Supplementary figure S43. Genomic profile of pairs of identical slow codons in *E. coli* (blue) *vs.* the *randomized* genomic profile of pairs of identical slow codons in *E. coli* (control for the positions of the slow codons). The ramp is *not* significant when controlling for the positions of slow codons (p = 1) .

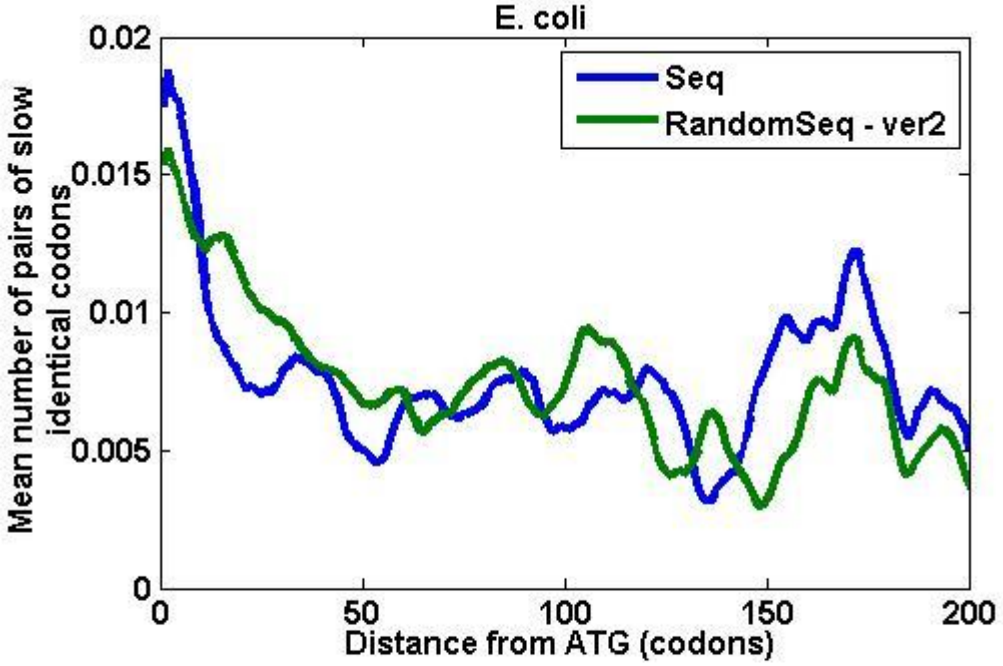

Supplementary figure S44. Genomic profile of pairs of identical slow codons in *E. coli* (blue) for highly expressed genes *vs.* the *randomized* genomic profile of pairs of identical slow codons in *E. coli* for highly expressed genes (control for the positions of slow codons). The ramp remains *lower* than expected when controlling for the positions of slow codons but it is not significant.

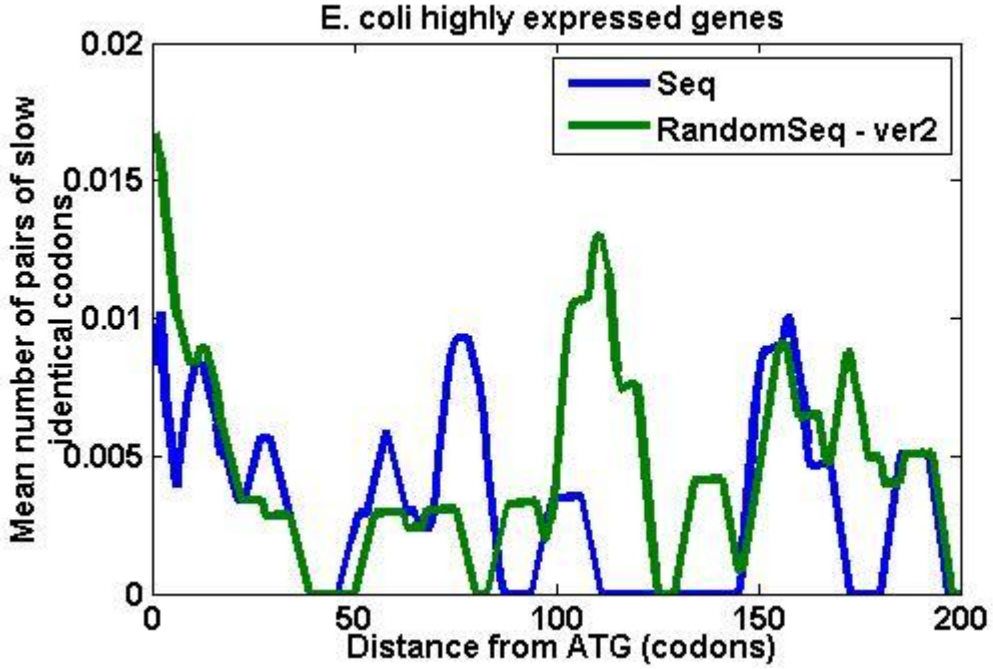

Supplementary figure S45. Genomic profile of pairs of identical slow codons in *E. coli* (blue) for lowly expressed genes *vs.* the *randomized* genomic profile of pairs of identical slow codons in *E. coli* for lowly expressed genes (control for the positions of slow codons). The ramp is not significant when controlling for the positions of slow codons.

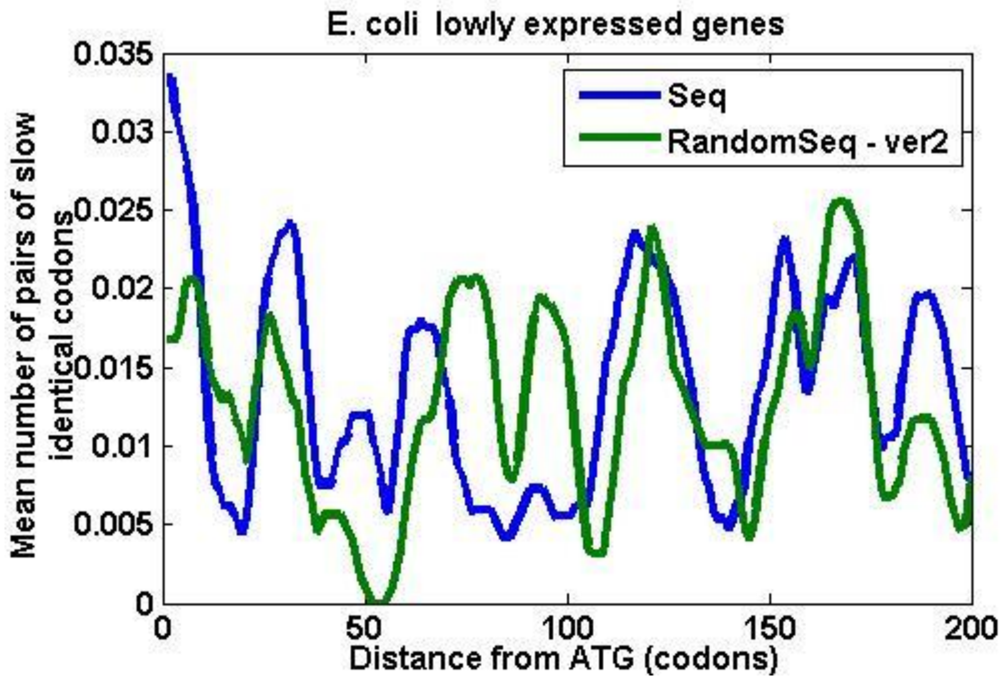

Supplementary figure S46. Gene length distribution in *S. cerevisiae* (in units of 200 codons).

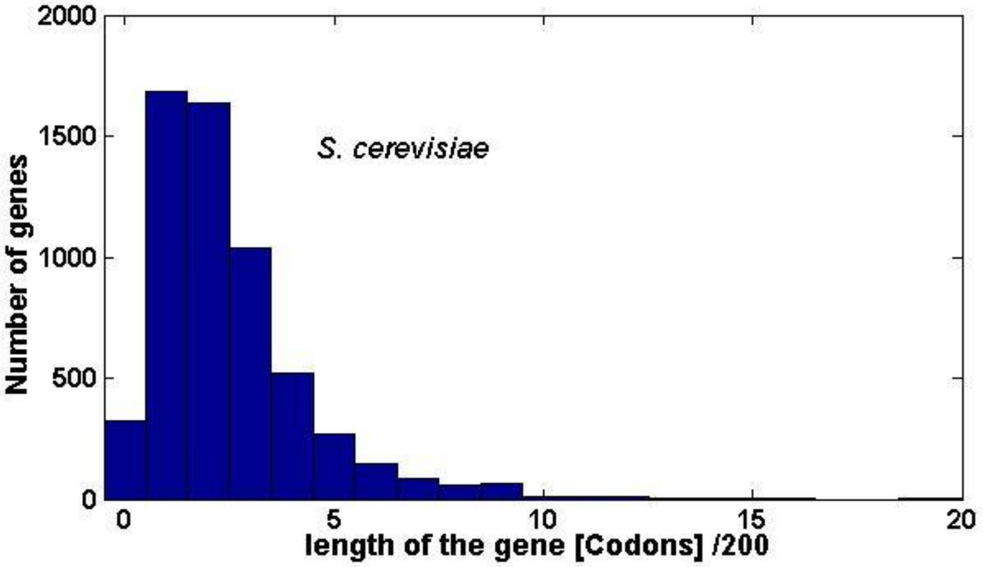

Supplement: Additional file 2 — Supplementary Figures S1 to S7 and S9 to S46. [file gb-2011-12-11-r110-S2.PDF]
