# Supplementary material for: Composite effects of gene determinants on the translation speed and density of ribosomes
Source: Genome Biol. 2011 Nov 3;12(11):R110. doi: 10.1186/gb-2011-12-11-r110 (PMC3334596; doi:10.1186/gb-2011-12-11-r110)
Supplement: Additional file 4 — Supplementary Figure S8. [file gb-2011-12-11-r110-S4.PDF]

Supplementary Figure S8 - The profiles of charge (A.), folding energy (B.), tAI (C.) for 42 GO slim groups. Each figure also includes the length of the ramp and a corresponding p-value. See also a summary in supplementary table 1.

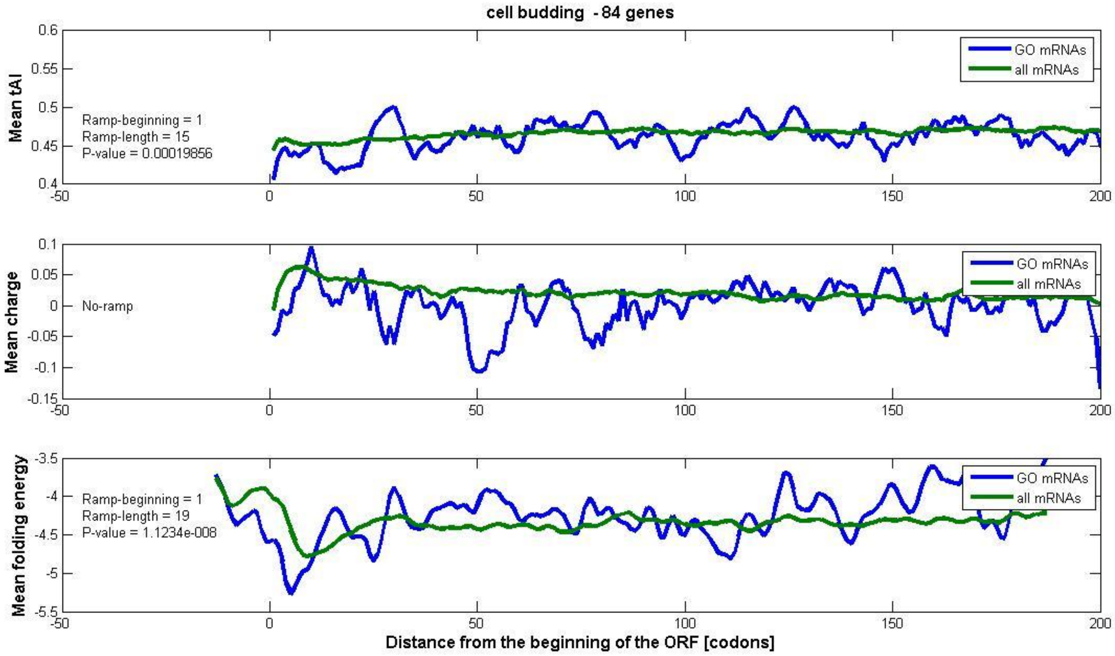

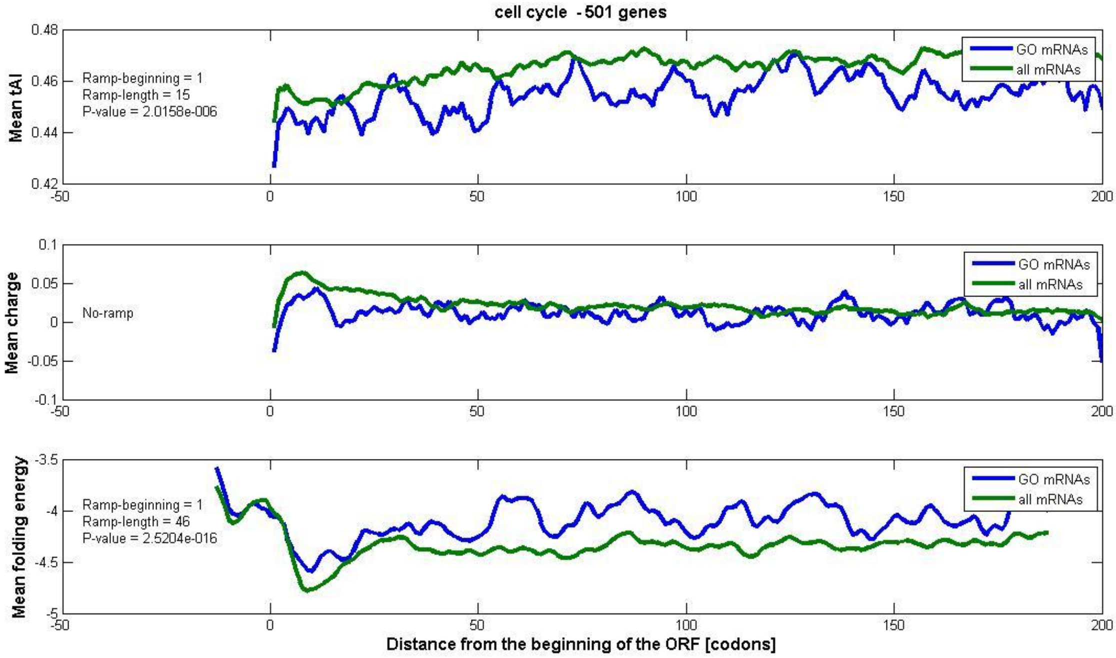

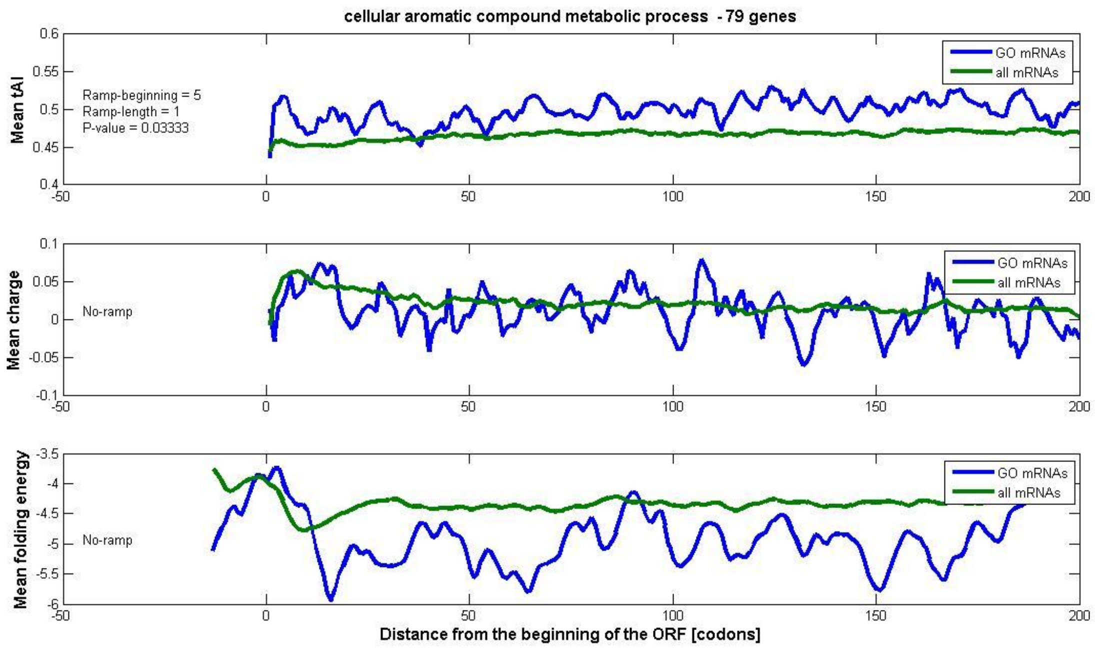

cellular aromatic compound metabolic process - 79 genes
Mean tAI
Ramp-beginning = 5
Ramp-length = 1
P-value = 0.03333
GO mRNAs
all mRNAs
Mean charge
No-ramp
Mean folding energy
No-ramp
Distance from the beginning of the ORF [codons]

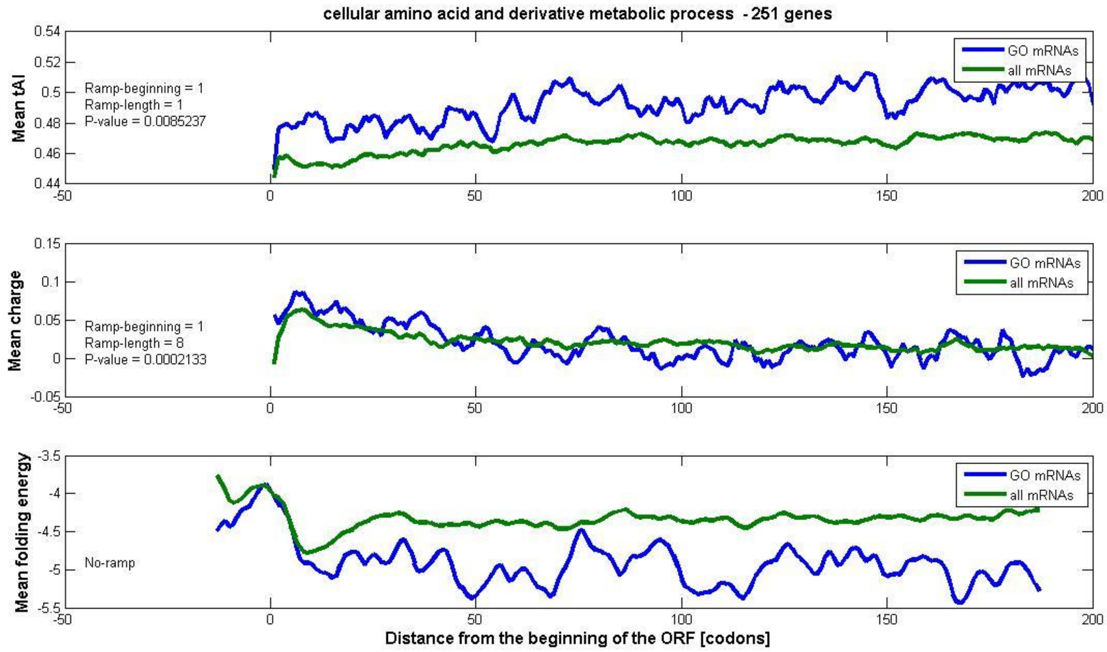

cellular amino acid and derivative metabolic process - 251 genes
Mean tAI
Ramp-beginning = 1
Ramp-length = 1
P-value = 0.0085237
GO mRNAs
all mRNAs
Mean charge
Ramp-beginning = 1
Ramp-length = 8
P-value = 0.0002133
Mean folding energy
No-ramp
Distance from the beginning of the ORF [codons]

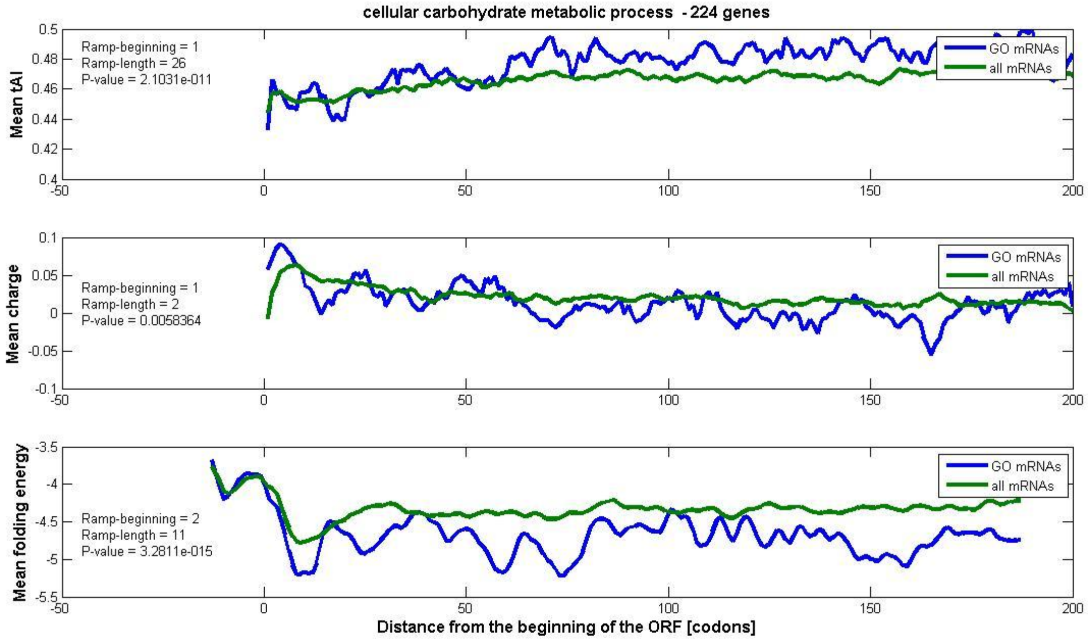
cellular carbohydrate metabolic process - 224 genes
Mean tAI
Ramp-beginning = 1
Ramp-length = 26
P-value = 2.1031e-011
GO mRNAs
all mRNAs
Mean charge
Ramp-beginning = 1
Ramp-length = 2
P-value = 0.0058364
GO mRNAs
all mRNAs
Mean folding energy
Ramp-beginning = 2
Ramp-length = 11
P-value = 3.2811e-015
GO mRNAs
all mRNAs
Distance from the beginning of the ORF [codons]

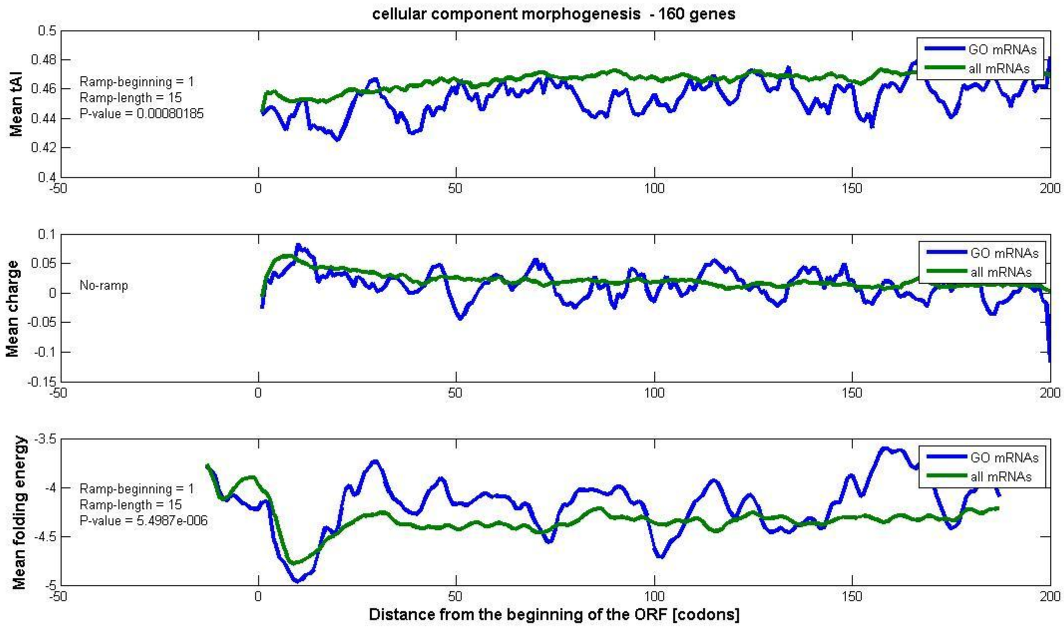
cellular component morphogenesis - 160 genes
Mean tAI
Ramp-beginning = 1
Ramp-length = 15
P-value = 0.00080185
GO mRNAs
all mRNAs
Mean charge
No-ramp
GO mRNAs
all mRNAs
Mean folding energy
Ramp-beginning = 1
Ramp-length = 15
P-value = 5.4987e-006
GO mRNAs
all mRNAs
Distance from the beginning of the ORF [codons]

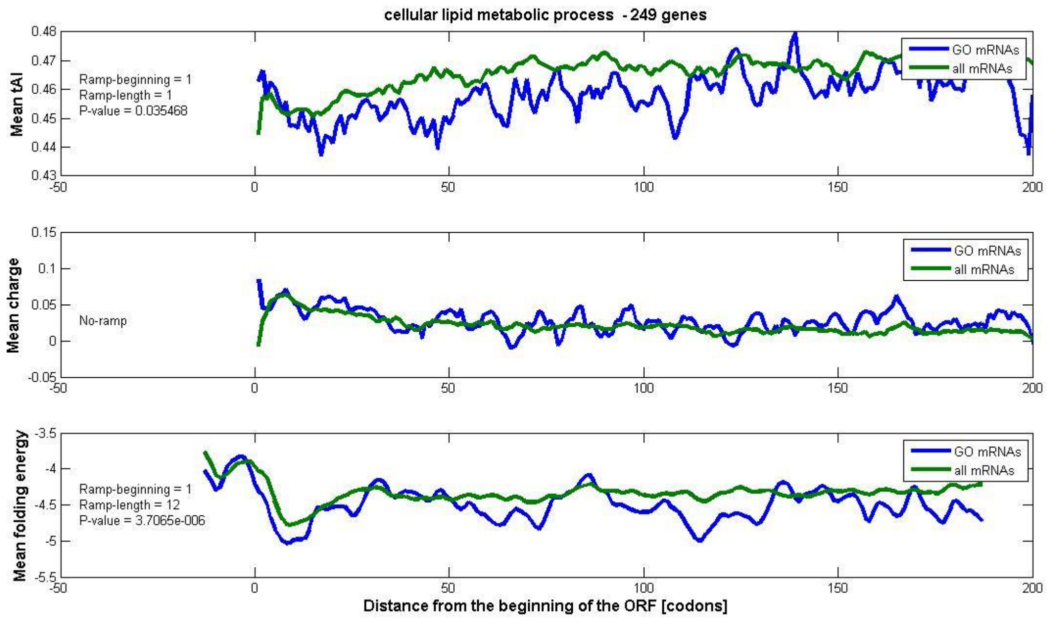
cellular lipid metabolic process - 249 genes
Mean tAI
Ramp-beginning = 1
Ramp-length = 1
P-value = 0.035468
GO mRNAs
all mRNAs
Mean charge
No-ramp
Mean folding energy
Ramp-beginning = 1
Ramp-length = 12
P-value = 3.7065e-006
Distance from the beginning of the ORF [codons]

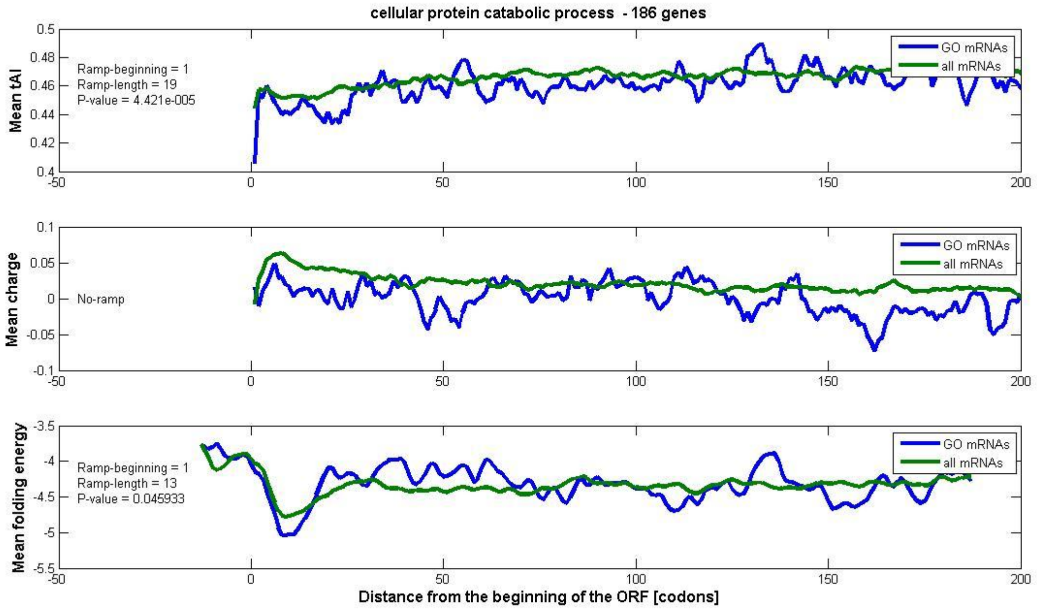
cellular protein catabolic process - 186 genes
Mean tAI
Ramp-beginning = 1
Ramp-length = 19
P-value = 4.421e-005
GO mRNAs
all mRNAs
Mean charge
No-ramp
Mean folding energy
Ramp-beginning = 1
Ramp-length = 13
P-value = 0.045933
Distance from the beginning of the ORF [codons]

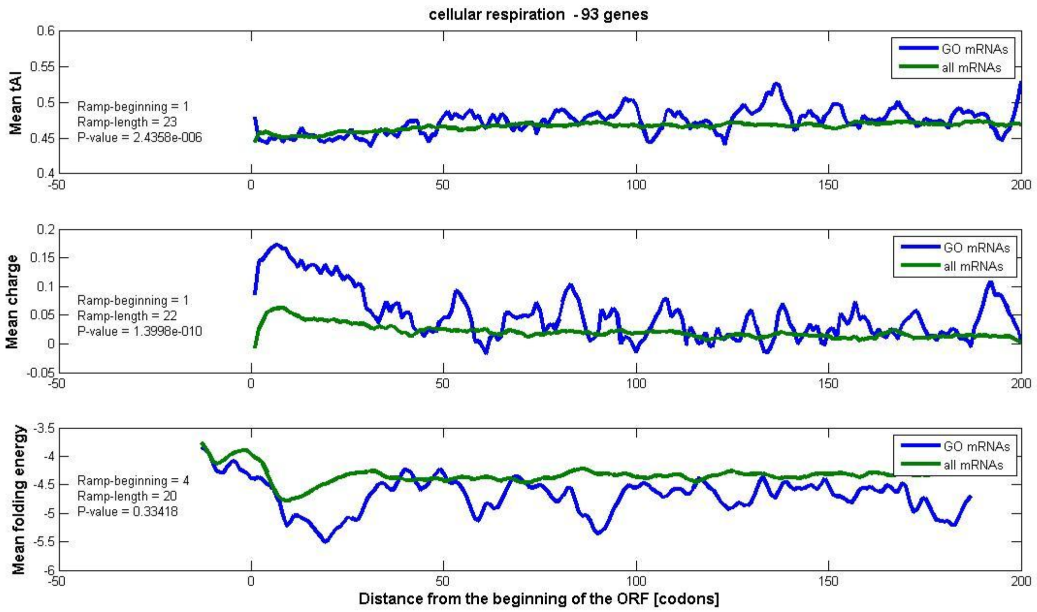
cellular respiration - 93 genes
Mean tAI
Ramp-beginning = 1
Ramp-length = 23
P-value = 2.4358e-006
Mean charge
Ramp-beginning = 1
Ramp-length = 22
P-value = 1.3998e-010
Mean folding energy
Ramp-beginning = 4
Ramp-length = 20
P-value = 0.33418
GO mRNAs
all mRNAs
Distance from the beginning of the ORF [codons]

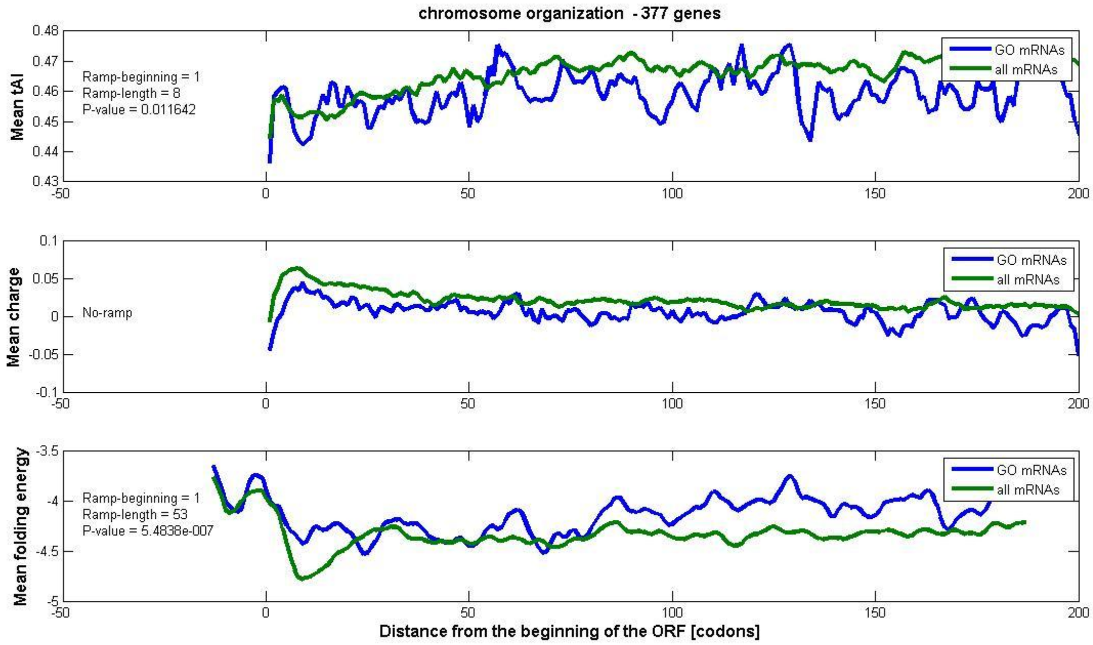
chromosome organization - 377 genes
Mean tAI
Ramp-beginning = 1
Ramp-length = 8
P-value = 0.011642
Mean charge
No-ramp
Mean folding energy
Ramp-beginning = 1
Ramp-length = 53
P-value = 5.4838e-007
GO mRNAs
all mRNAs
Distance from the beginning of the ORF [codons]

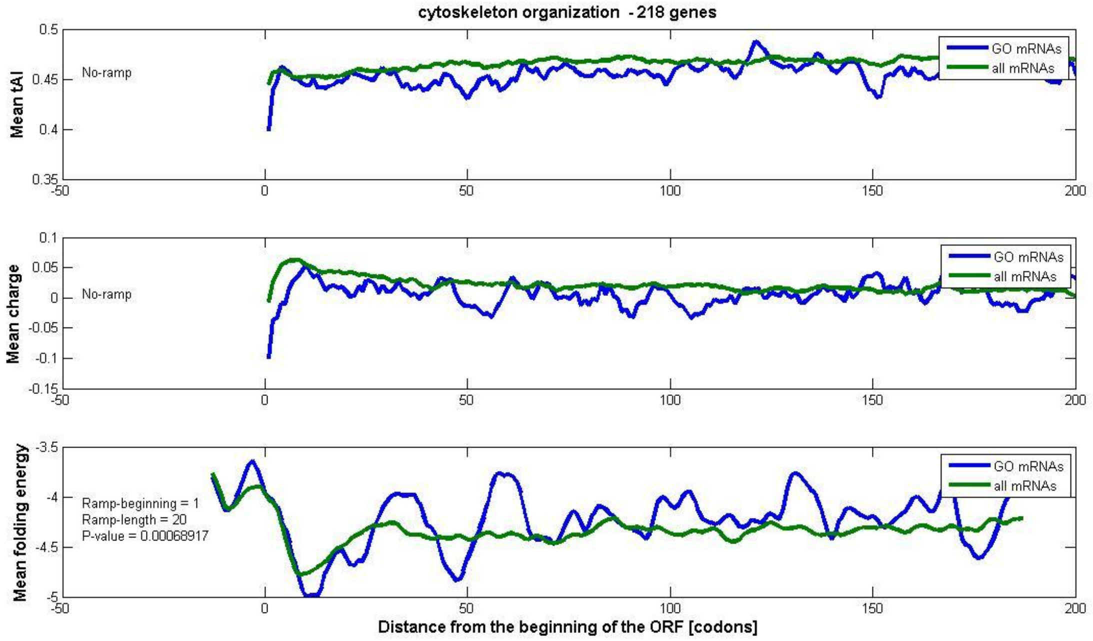
cytoskeleton organization - 218 genes
Mean tAI
No-ramp
GO mRNAs
all mRNAs
Mean charge
No-ramp
Mean folding energy
Ramp-beginning = 1
Ramp-length = 20
P-value = 0.00068917
Distance from the beginning of the ORF [codons]

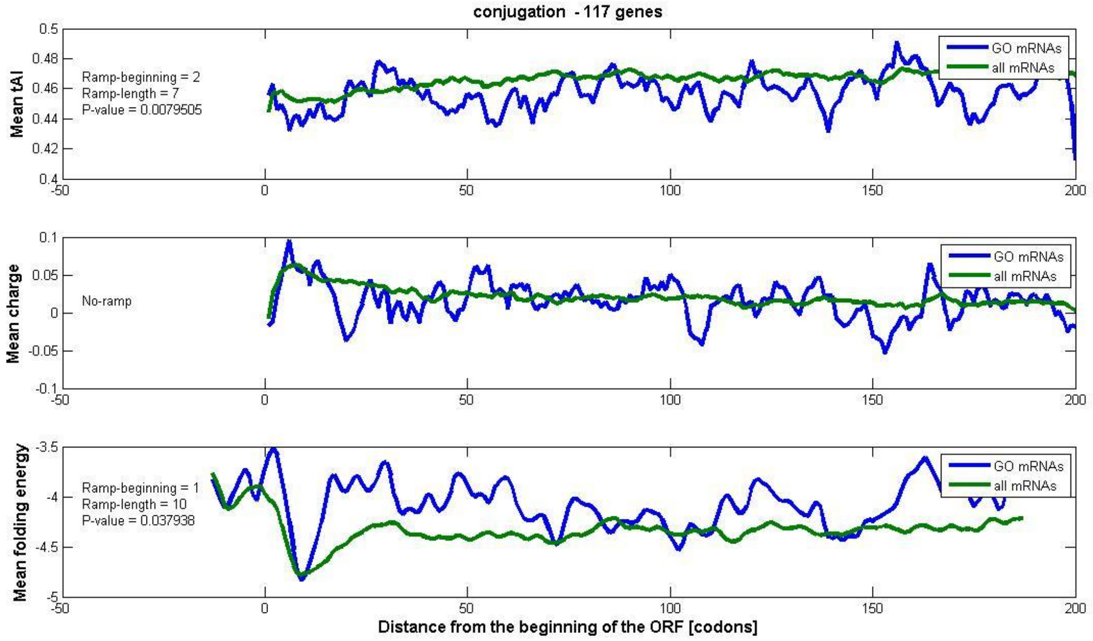
conjugation - 117 genes
Mean tAI
Ramp-beginning = 2
Ramp-length = 7
P-value = 0.0079505
GO mRNAs
all mRNAs
Mean charge
No-ramp
Mean folding energy
Ramp-beginning = 1
Ramp-length = 10
P-value = 0.037938
Distance from the beginning of the ORF [codons]

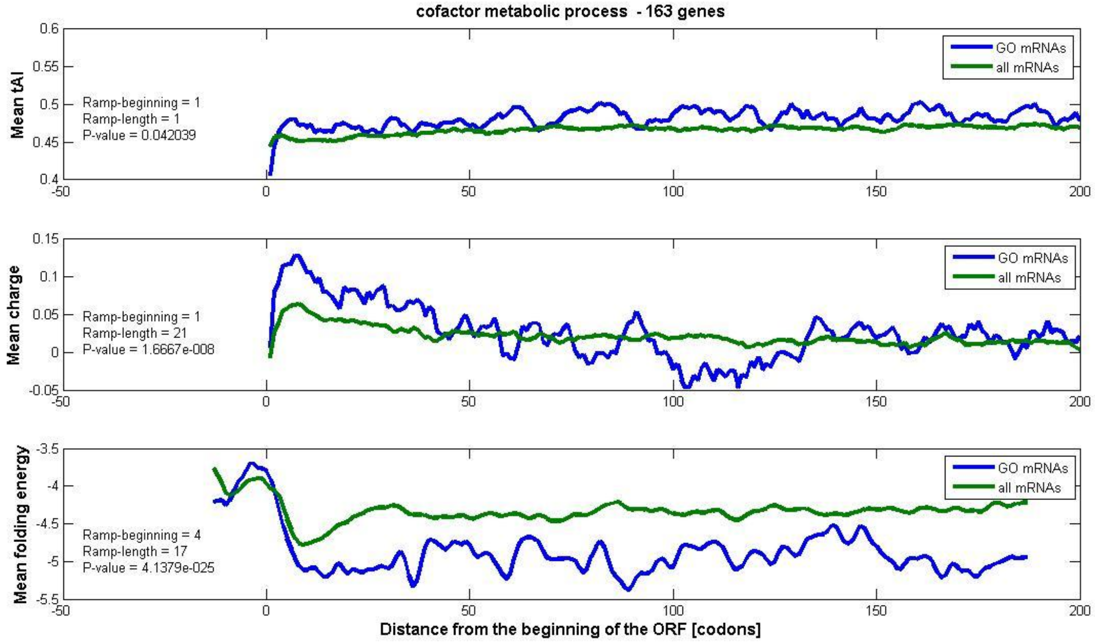
cofactor metabolic process - 163 genes
Mean tAI
Ramp-beginning = 1
Ramp-length = 1
P-value = 0.042039
Mean charge
Ramp-beginning = 1
Ramp-length = 21
P-value = 1.6667e-008
Mean folding energy
Ramp-beginning = 4
Ramp-length = 17
P-value = 4.1379e-025
GO mRNAs
all mRNAs
Distance from the beginning of the ORF [codons]

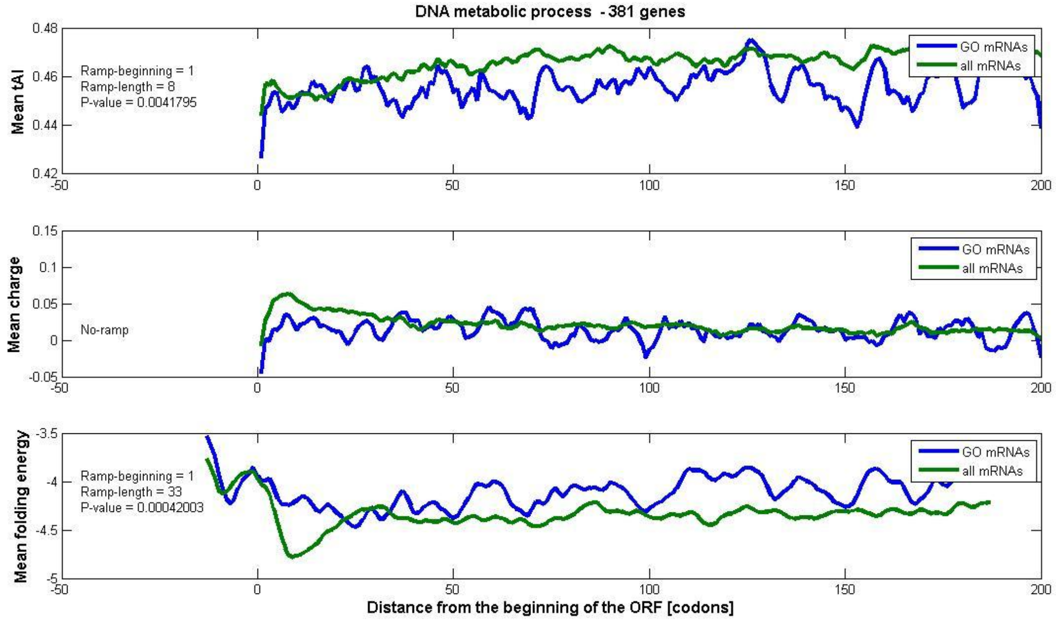
DNA metabolic process - 381 genes
Mean tAI
Ramp-beginning = 1
Ramp-length = 8
P-value = 0.0041795
Mean charge
No-ramp
Mean folding energy
Ramp-beginning = 1
Ramp-length = 33
P-value = 0.00042003
GO mRNAs
all mRNAs
Distance from the beginning of the ORF [codons]

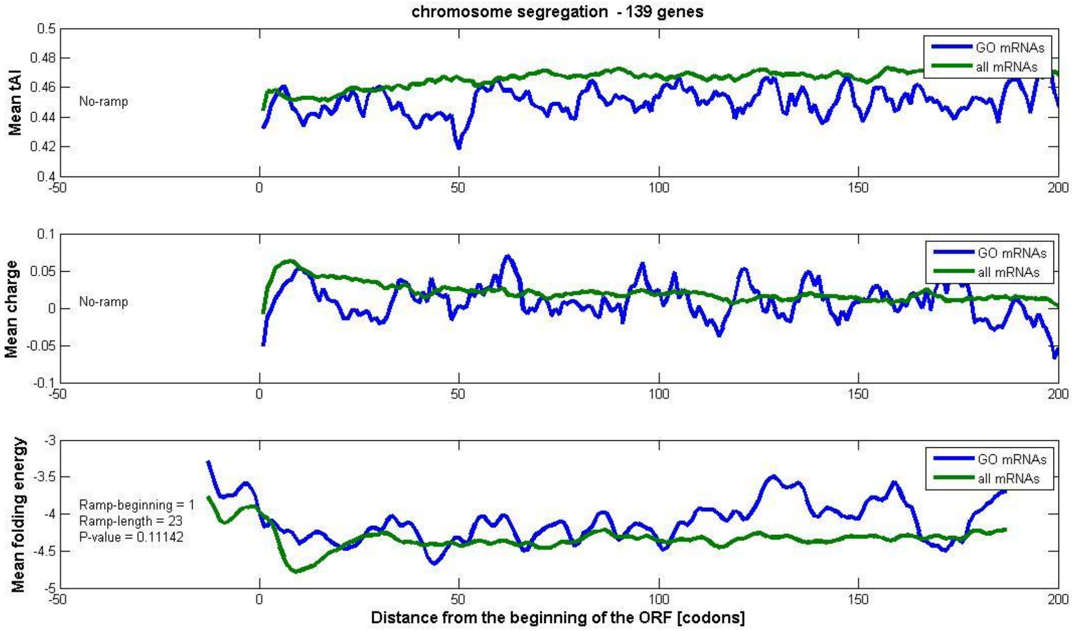
chromosome segregation - 139 genes
Mean tAI
No-ramp
GO mRNAs
all mRNAs
Mean charge
No-ramp
GO mRNAs
all mRNAs
Mean folding energy
Ramp-beginning = 1
Ramp-length = 23
P-value = 0.11142
GO mRNAs
all mRNAs
Distance from the beginning of the ORF [codons]

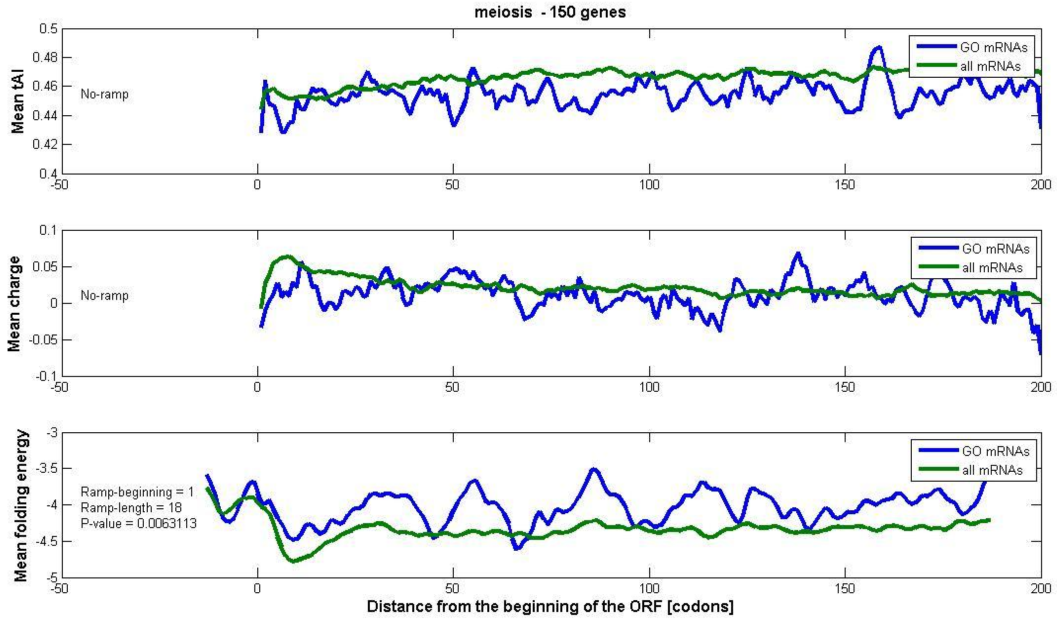
meiosis - 150 genes
Mean tAI
No-ramp
GO mRNAs
all mRNAs
Mean charge
No-ramp
GO mRNAs
all mRNAs
Mean folding energy
Ramp-beginning = 1
Ramp-length = 18
P-value = 0.0063113
GO mRNAs
all mRNAs
Distance from the beginning of the ORF [codons]

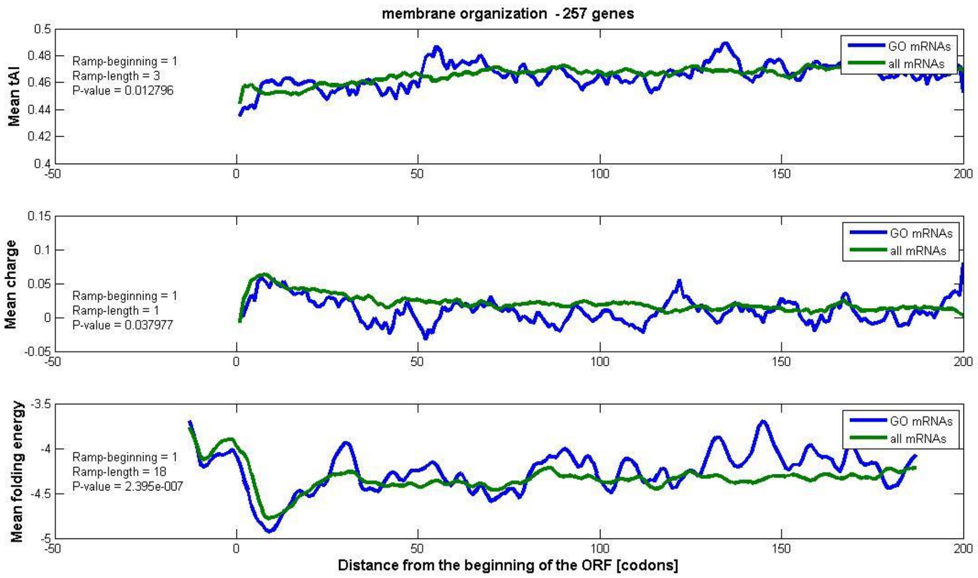
membrane organization - 257 genes
Mean tAI
Ramp-beginning = 1
Ramp-length = 3
P-value = 0.012796
GO mRNAs
all mRNAs
Mean charge
Ramp-beginning = 1
Ramp-length = 1
P-value = 0.037977
Mean folding energy
Ramp-beginning = 1
Ramp-length = 18
P-value = 2.395e-007
Distance from the beginning of the ORF [codons]

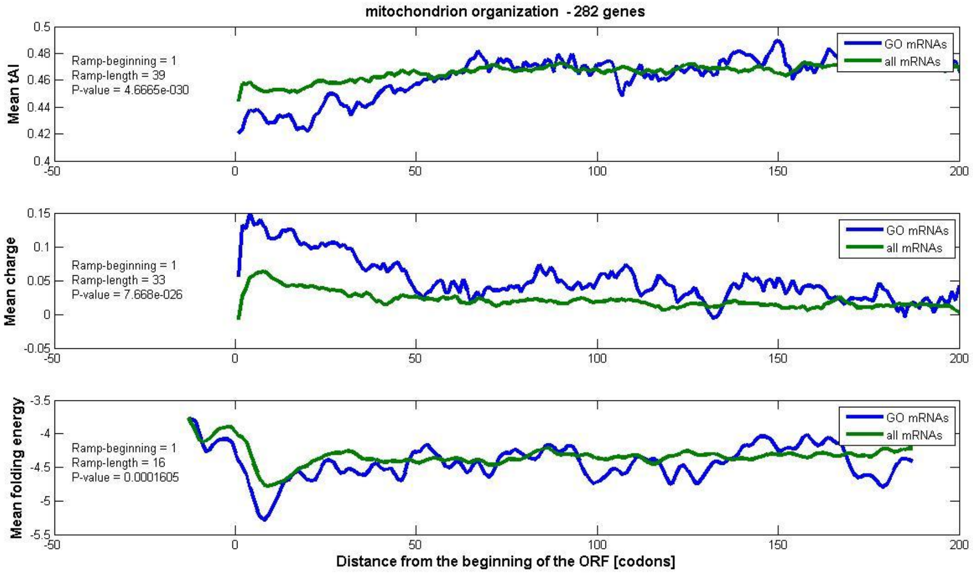
mitochondrion organization - 282 genes
Mean tAI
Ramp-beginning = 1
Ramp-length = 39
P-value = 4.6665e-030
GO mRNAs
all mRNAs
Mean charge
Ramp-beginning = 1
Ramp-length = 33
P-value = 7.668e-026
Mean folding energy
Ramp-beginning = 1
Ramp-length = 16
P-value = 0.0001605
Distance from the beginning of the ORF [codons]

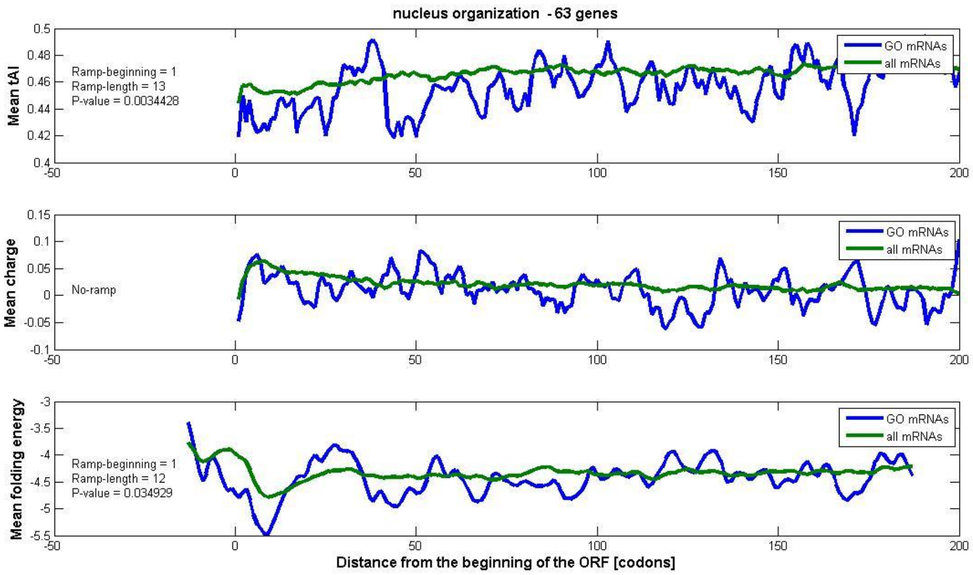
nucleus organization - 63 genes
Mean tAI
Ramp-beginning = 1
Ramp-length = 13
P-value = 0.0034428
GO mRNAs
all mRNAs
Mean charge
No-ramp
Mean folding energy
Ramp-beginning = 1
Ramp-length = 12
P-value = 0.034929
Distance from the beginning of the ORF [codons]

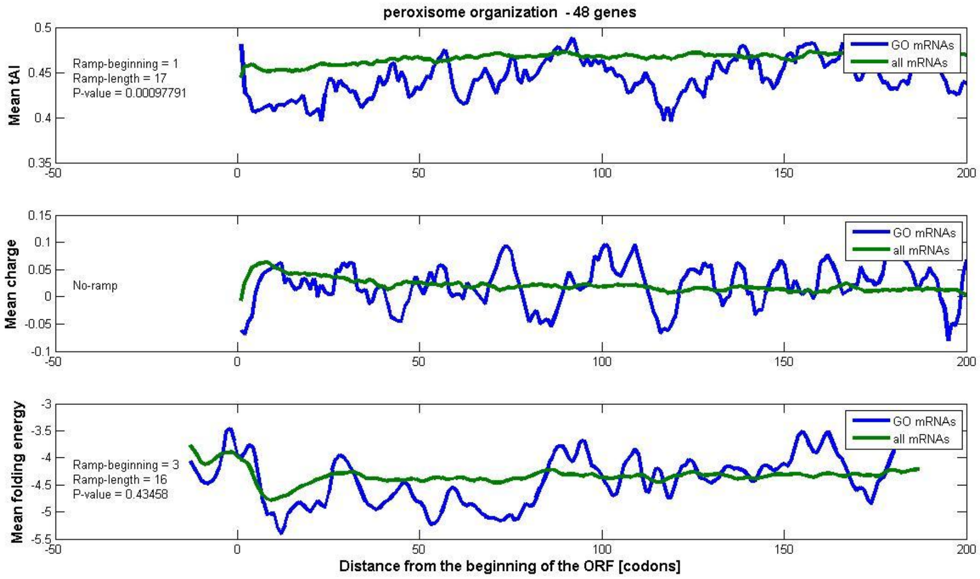
peroxisome organization - 48 genes
Mean tAI
Ramp-beginning = 1
Ramp-length = 17
P-value = 0.00097791
GO mRNAs
all mRNAs
Mean charge
No-ramp
Mean folding energy
Ramp-beginning = 3
Ramp-length = 16
P-value = 0.43458
Distance from the beginning of the ORF [codons]

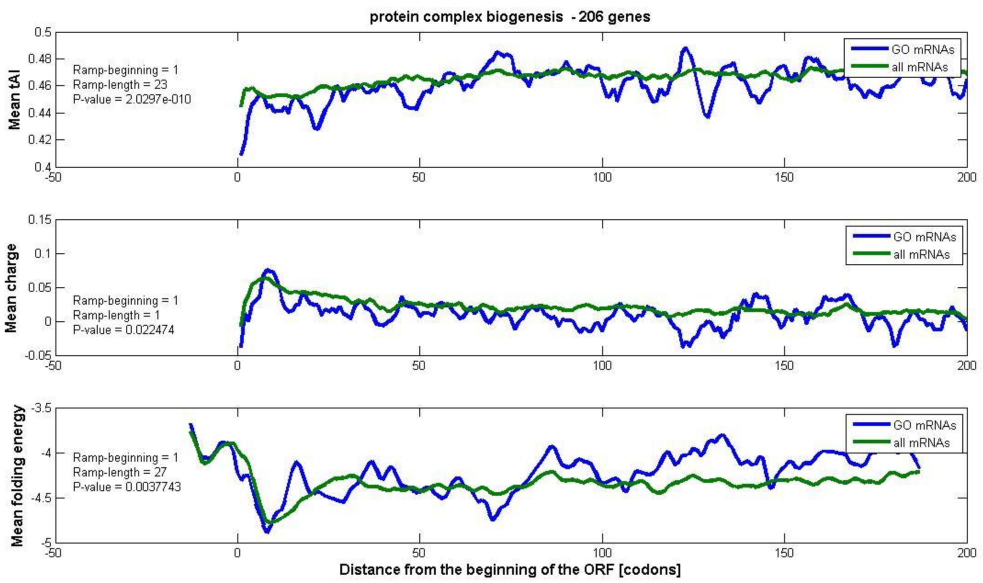
protein complex biogenesis - 206 genes
Mean tAI
Ramp-beginning = 1
Ramp-length = 23
P-value = 2.0297e-010
GO mRNAs
all mRNAs
Mean charge
Ramp-beginning = 1
Ramp-length = 1
P-value = 0.022474
GO mRNAs
all mRNAs
Mean folding energy
Ramp-beginning = 1
Ramp-length = 27
P-value = 0.0037743
GO mRNAs
all mRNAs
Distance from the beginning of the ORF [codons]

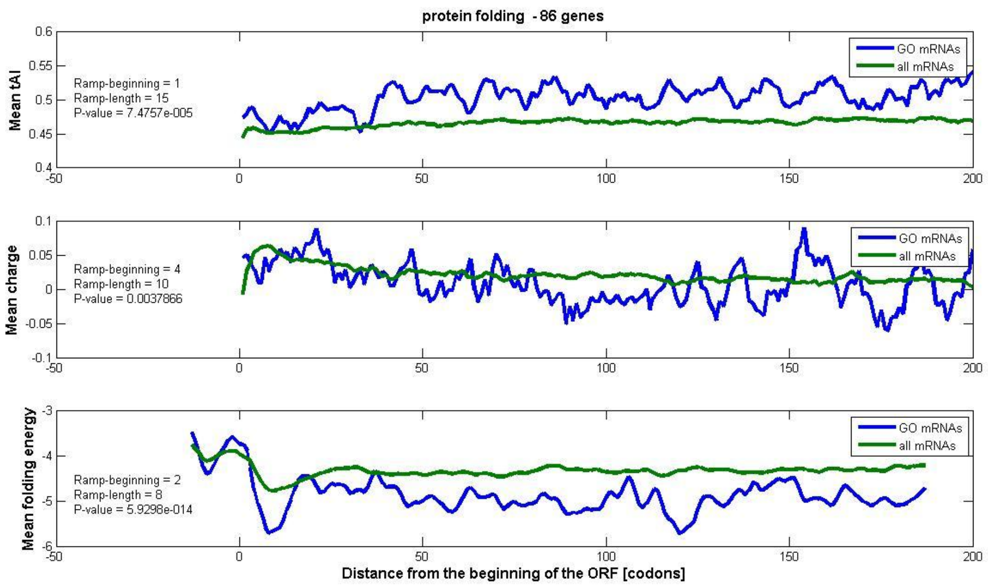
protein folding - 86 genes
Mean tAI
Ramp-beginning = 1
Ramp-length = 15
P-value = 7.4757e-005
GO mRNAs
all mRNAs
Mean charge
Ramp-beginning = 4
Ramp-length = 10
P-value = 0.0037866
GO mRNAs
all mRNAs
Mean folding energy
Ramp-beginning = 2
Ramp-length = 8
P-value = 5.9298e-014
GO mRNAs
all mRNAs
Distance from the beginning of the ORF [codons]

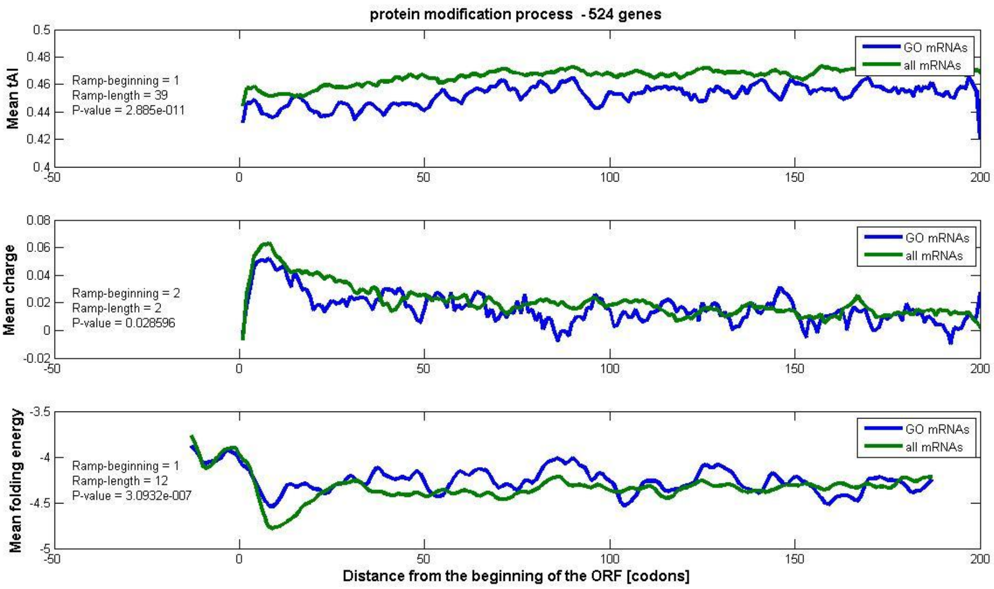
protein modification process - 524 genes
Mean tAI
Ramp-beginning = 1
Ramp-length = 39
P-value = 2.885e-011
GO mRNAs
all mRNAs
Mean charge
Ramp-beginning = 2
Ramp-length = 2
P-value = 0.028596
Mean folding energy
Ramp-beginning = 1
Ramp-length = 12
P-value = 3.0932e-007
Distance from the beginning of the ORF [codons]

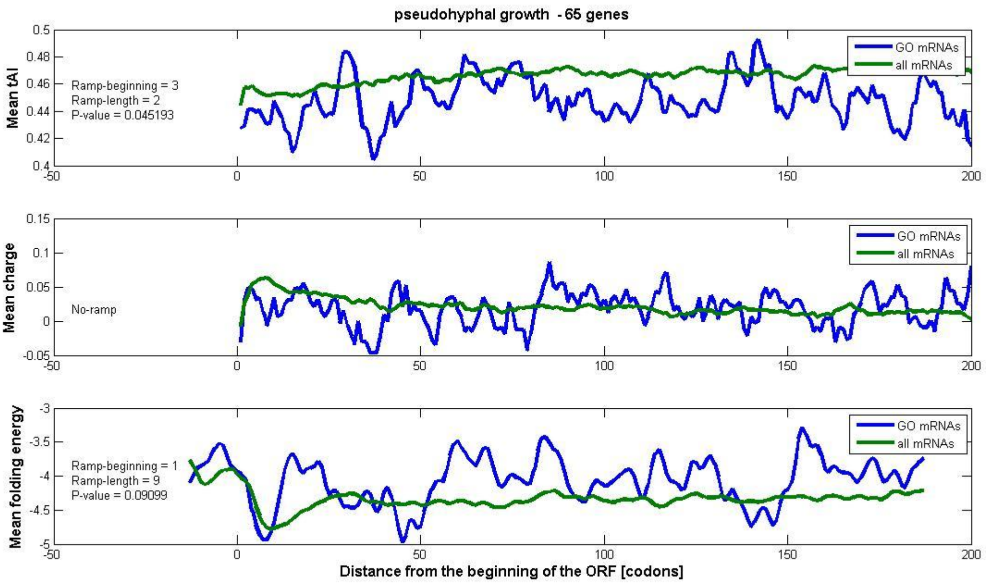
pseudohyphal growth - 65 genes
Mean tAI
Ramp-beginning = 3
Ramp-length = 2
P-value = 0.045193
GO mRNAs
all mRNAs
Mean charge
No-ramp
Mean folding energy
Ramp-beginning = 1
Ramp-length = 9
P-value = 0.09099
Distance from the beginning of the ORF [codons]

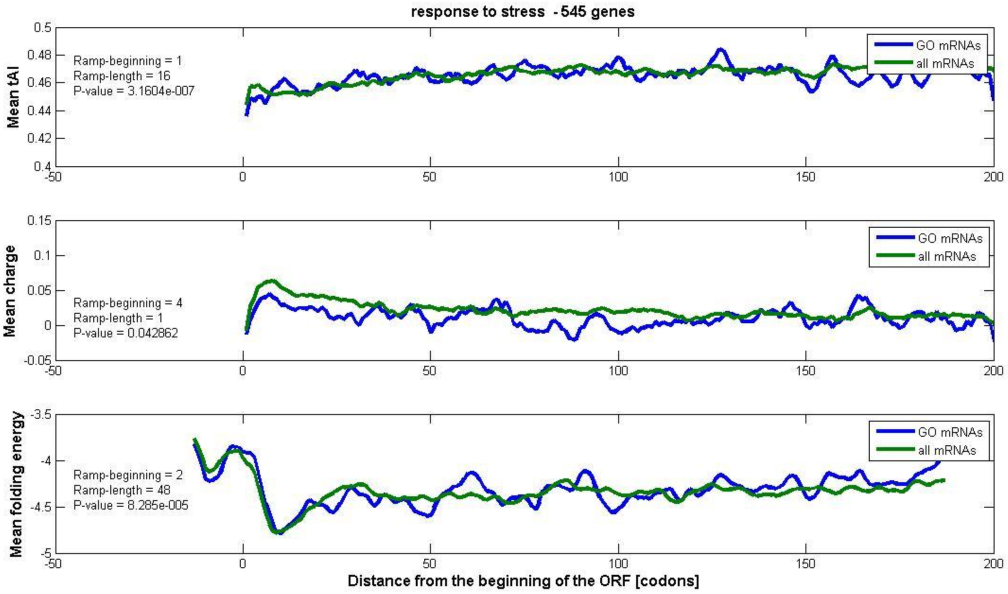
response to stress - 545 genes
Mean tAI
Ramp-beginning = 1
Ramp-length = 16
P-value = 3.1604e-007
GO mRNAs
all mRNAs
Mean charge
Ramp-beginning = 4
Ramp-length = 1
P-value = 0.042862
Mean folding energy
Ramp-beginning = 2
Ramp-length = 48
P-value = 8.285e-005
Distance from the beginning of the ORF [codons]

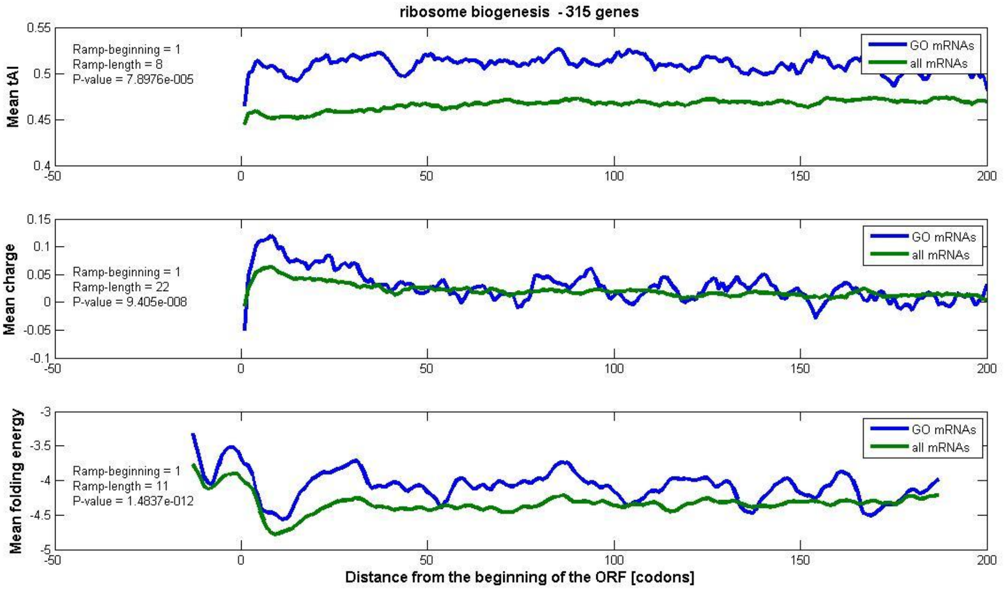
ribosome biogenesis - 315 genes
Mean tAI
Ramp-beginning = 1
Ramp-length = 8
P-value = 7.8976e-005
GO mRNAs
all mRNAs
Mean charge
Ramp-beginning = 1
Ramp-length = 22
P-value = 9.405e-008
Mean folding energy
Ramp-beginning = 1
Ramp-length = 11
P-value = 1.4837e-012
Distance from the beginning of the ORF [codons]

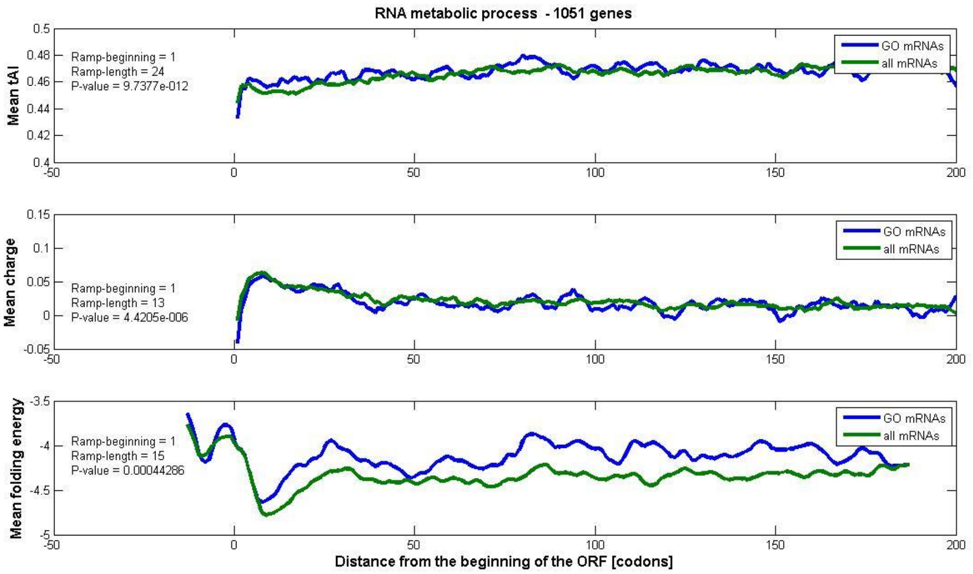
RNA metabolic process - 1051 genes
Mean tAI
Ramp-beginning = 1
Ramp-length = 24
P-value = 9.7377e-012
Mean charge
Ramp-beginning = 1
Ramp-length = 13
P-value = 4.4205e-006
Mean folding energy
Ramp-beginning = 1
Ramp-length = 15
P-value = 0.00044286
GO mRNAs
all mRNAs
Distance from the beginning of the ORF [codons]

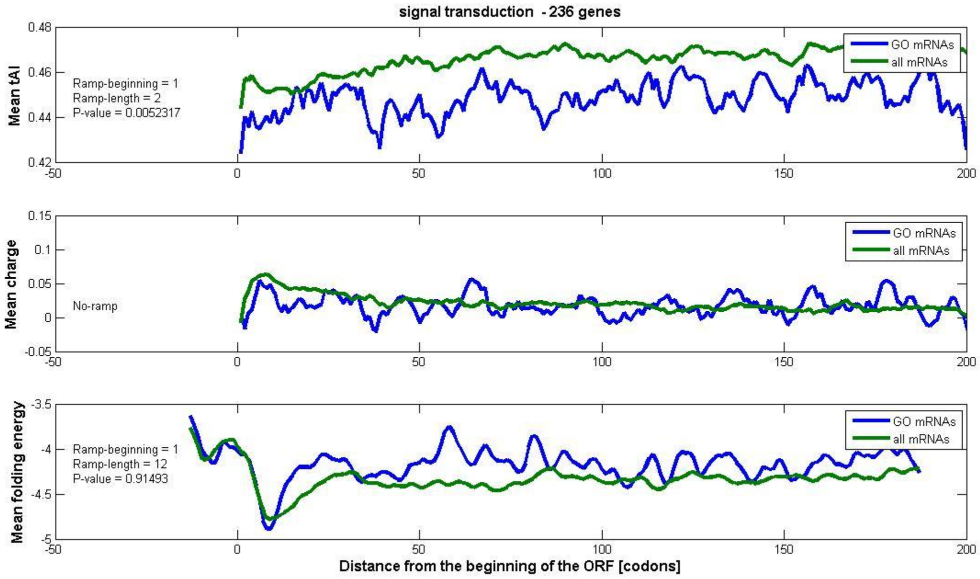
signal transduction - 236 genes
Mean tAI
Ramp-beginning = 1
Ramp-length = 2
P-value = 0.0052317
Mean charge
No-ramp
Mean folding energy
Ramp-beginning = 1
Ramp-length = 12
P-value = 0.91493
GO mRNAs
all mRNAs
Distance from the beginning of the ORF [codons]

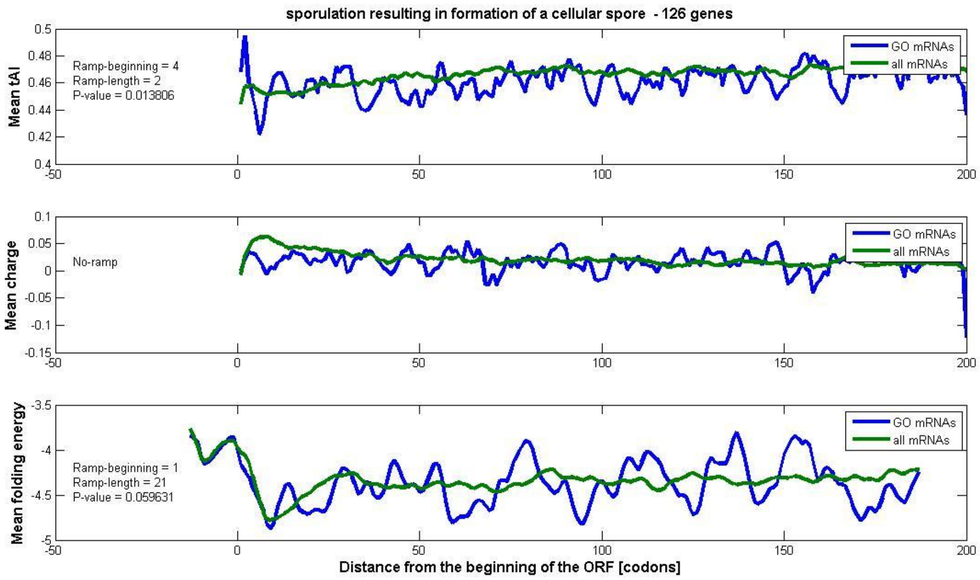
sporulation resulting in formation of a cellular spore - 126 genes
Mean tAI
Ramp-beginning = 4
Ramp-length = 2
P-value = 0.013806
GO mRNAs
all mRNAs
Mean charge
No-ramp
Mean folding energy
Ramp-beginning = 1
Ramp-length = 21
P-value = 0.059631
Distance from the beginning of the ORF [codons]

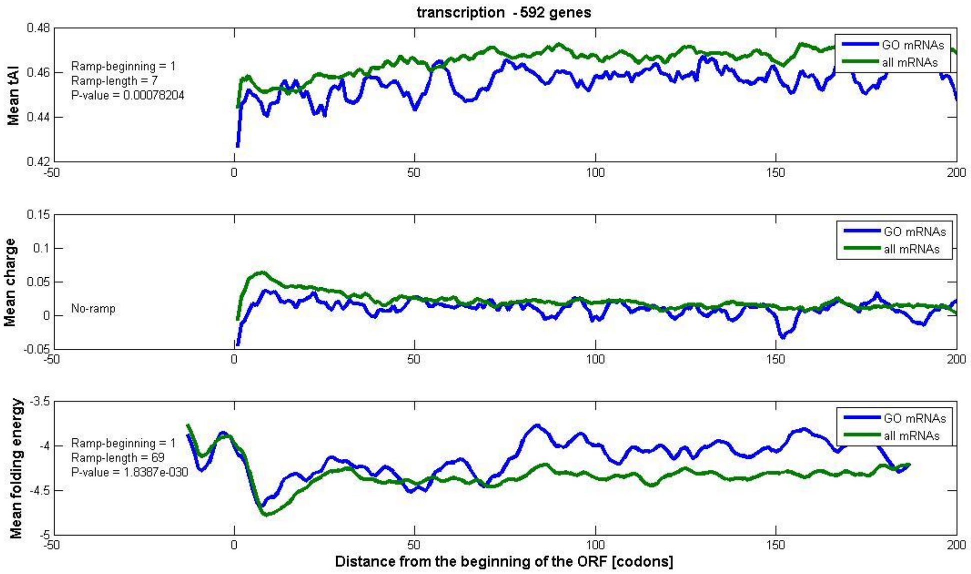
transcription - 592 genes
Mean tAI
Ramp-beginning = 1
Ramp-length = 7
P-value = 0.00078204
GO mRNAs
all mRNAs
Mean charge
No-ramp
Mean folding energy
Ramp-beginning = 1
Ramp-length = 69
P-value = 1.8387e-030
Distance from the beginning of the ORF [codons]

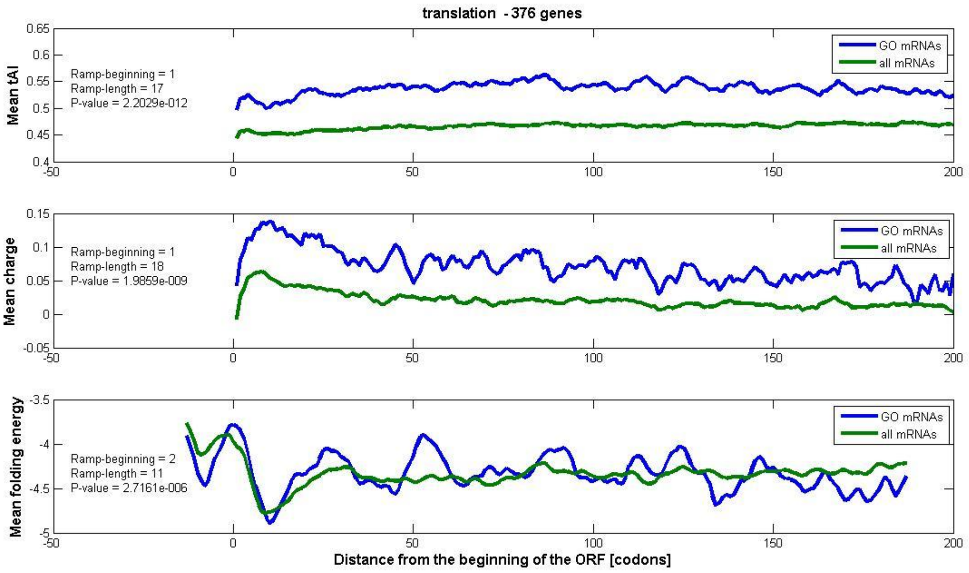
translation - 376 genes
Mean tAI
Ramp-beginning = 1
Ramp-length = 17
P-value = 2.2029e-012
GO mRNAs
all mRNAs
Mean charge
Ramp-beginning = 1
Ramp-length = 18
P-value = 1.9859e-009
GO mRNAs
all mRNAs
Mean folding energy
Ramp-beginning = 2
Ramp-length = 11
P-value = 2.7161e-006
GO mRNAs
all mRNAs
Distance from the beginning of the ORF [codons]

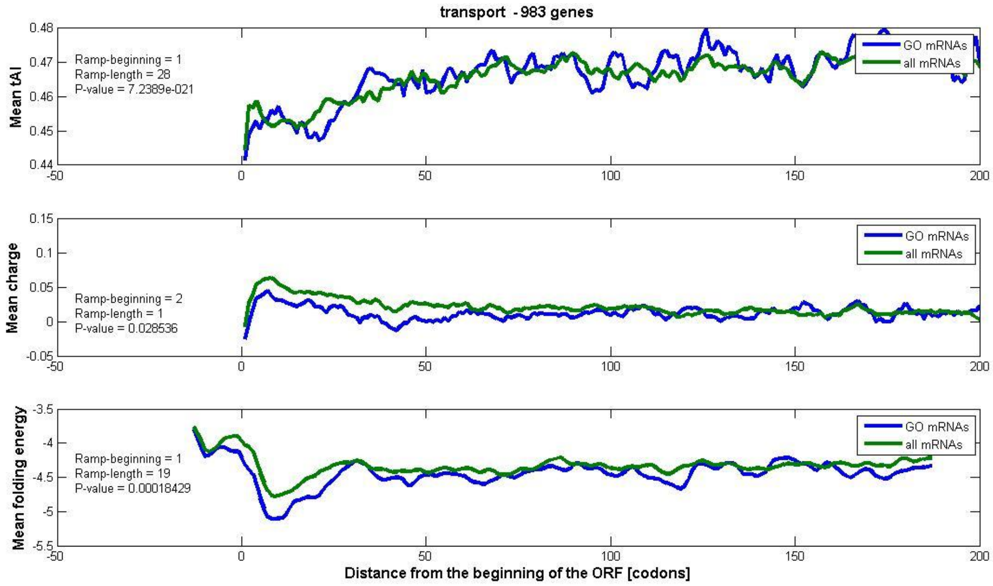
transport - 983 genes
Mean tAI
Ramp-beginning = 1
Ramp-length = 28
P-value = 7.2389e-021
GO mRNAs
all mRNAs
Mean charge
Ramp-beginning = 2
Ramp-length = 1
P-value = 0.028536
GO mRNAs
all mRNAs
Mean folding energy
Ramp-beginning = 1
Ramp-length = 19
P-value = 0.00018429
GO mRNAs
all mRNAs
Distance from the beginning of the ORF [codons]

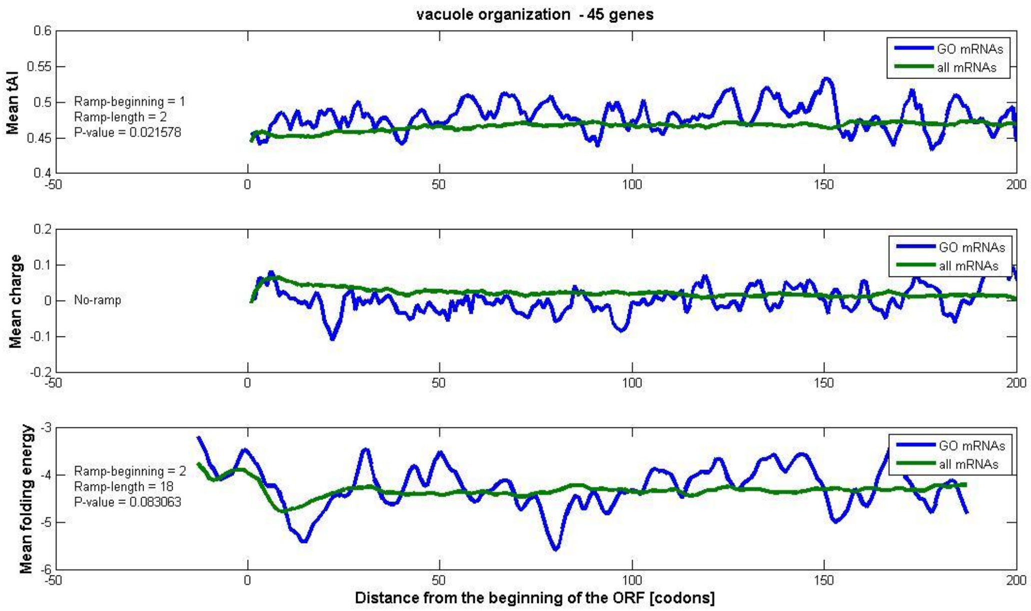

vacuole organization - 45 genes
Mean tAI
Ramp-beginning = 1
Ramp-length = 2
P-value = 0.021578
GO mRNAs
all mRNAs
Mean charge
No-ramp
Mean folding energy
Ramp-beginning = 2
Ramp-length = 18
P-value = 0.083063
Distance from the beginning of the ORF [codons]

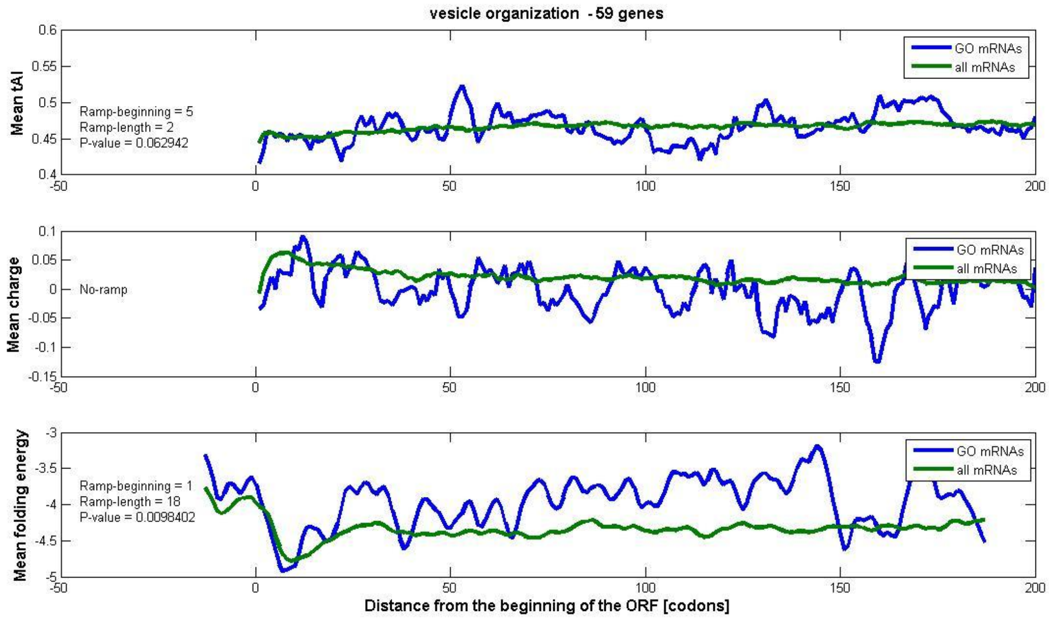

vesicle organization - 59 genes
Mean tAI
Ramp-beginning = 5
Ramp-length = 2
P-value = 0.062942
GO mRNAs
all mRNAs
Mean charge
No-ramp
Mean folding energy
Ramp-beginning = 1
Ramp-length = 18
P-value = 0.0098402
Distance from the beginning of the ORF [codons]

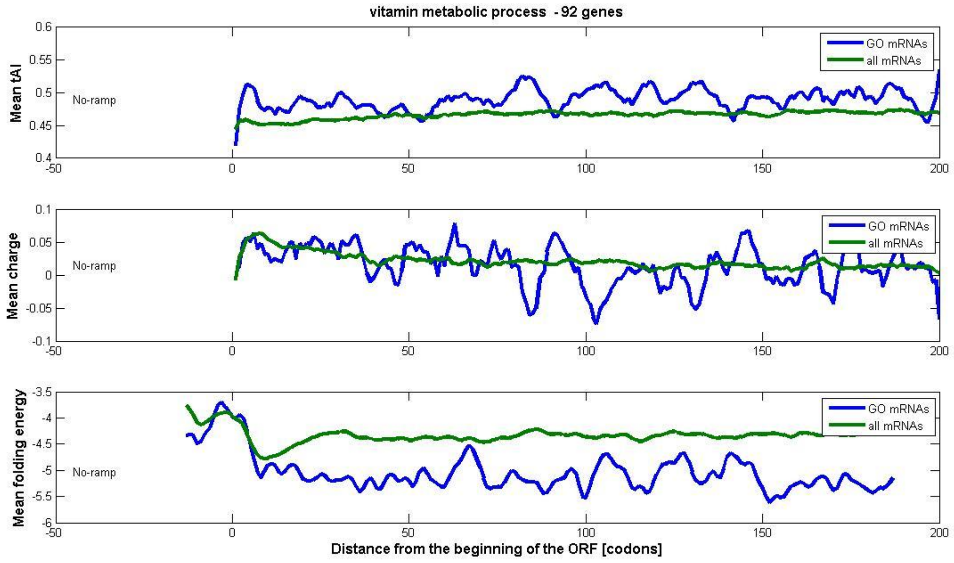
vitamin metabolic process - 92 genes
Mean tAI
No-ramp
GO mRNAs
all mRNAs
Mean charge
No-ramp
Mean folding energy
No-ramp
Distance from the beginning of the ORF [codons]

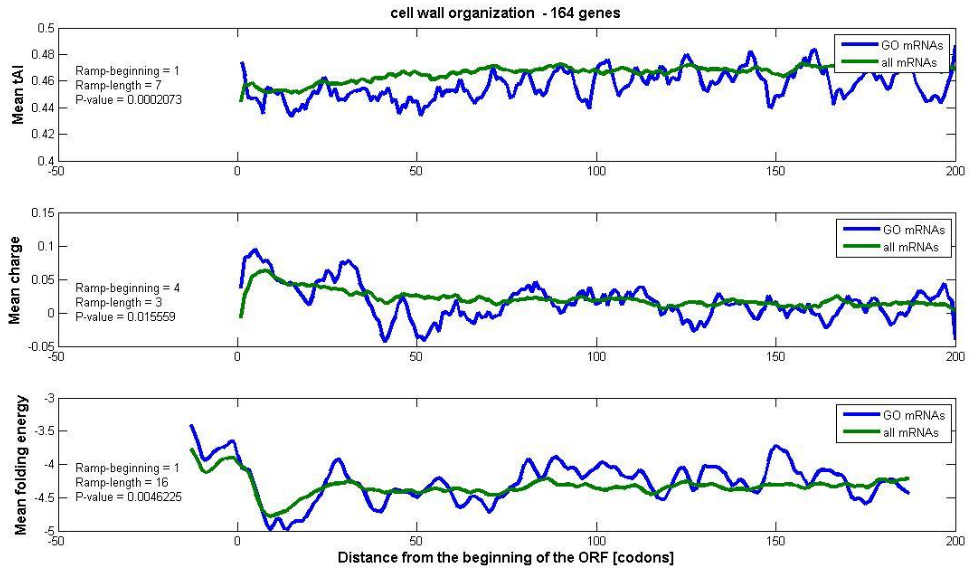
cell wall organization - 164 genes
Mean tAI
Ramp-beginning = 1
Ramp-length = 7
P-value = 0.0002073
GO mRNAs
all mRNAs
Mean charge
Ramp-beginning = 4
Ramp-length = 3
P-value = 0.015559
Mean folding energy
Ramp-beginning = 1
Ramp-length = 16
P-value = 0.0046225
Distance from the beginning of the ORF [codons]

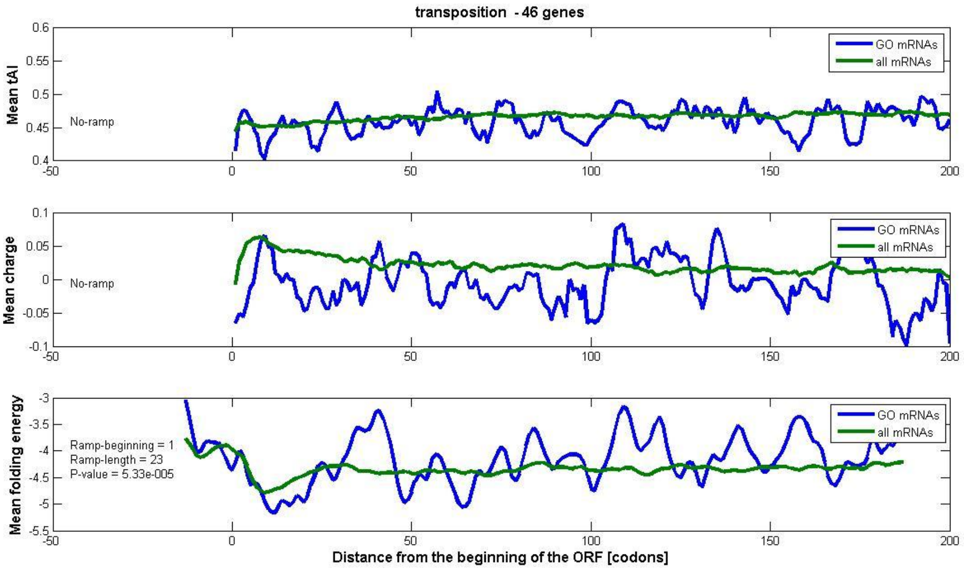
transposition - 46 genes
Mean tAI
No-ramp
GO mRNAs
all mRNAs
Mean charge
No-ramp
Mean folding energy
Ramp-beginning = 1
Ramp-length = 23
P-value = 5.33e-005
Distance from the beginning of the ORF [codons]

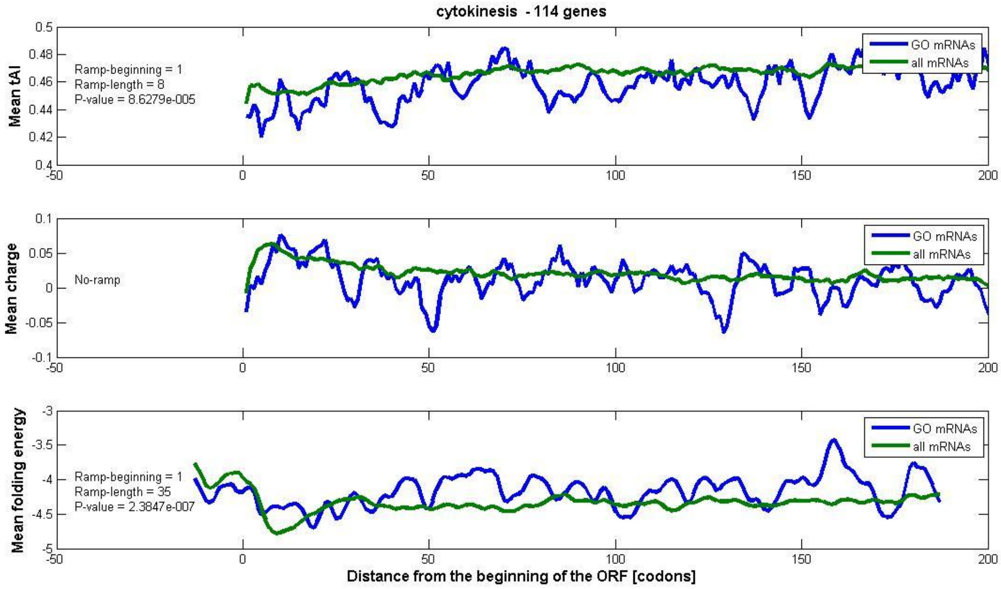
cytokinesis - 114 genes
Mean tAI
Ramp-beginning = 1
Ramp-length = 8
P-value = 8.6279e-005
GO mRNAs
all mRNAs
Mean charge
No-ramp
Mean folding energy
Ramp-beginning = 1
Ramp-length = 35
P-value = 2.3847e-007
Distance from the beginning of the ORF [codons]

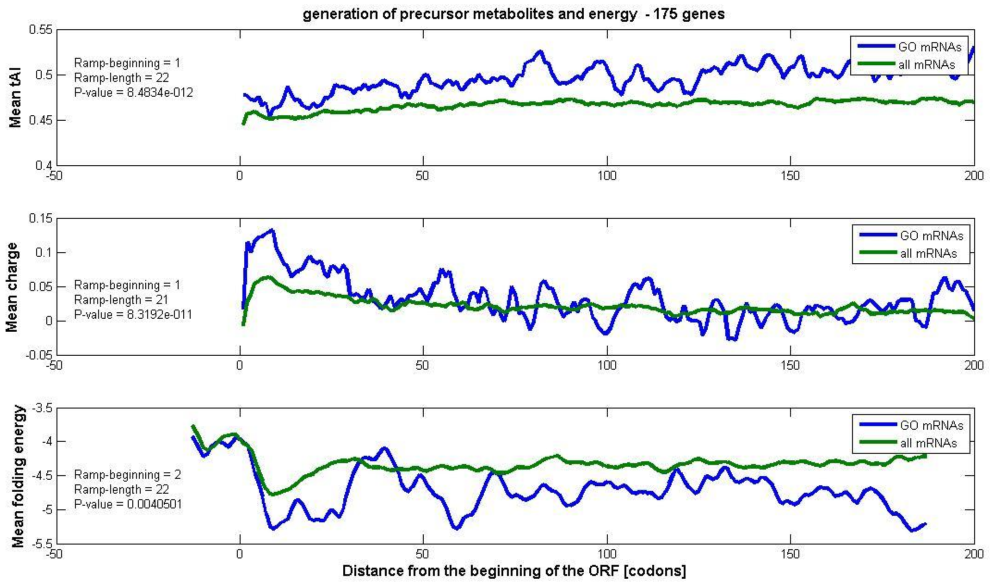
generation of precursor metabolites and energy - 175 genes
Mean tAI
Ramp-beginning = 1
Ramp-length = 22
P-value = 8.4834e-012
GO mRNAs
all mRNAs
Mean charge
Ramp-beginning = 1
Ramp-length = 21
P-value = 8.3192e-011
Mean folding energy
Ramp-beginning = 2
Ramp-length = 22
P-value = 0.0040501
Distance from the beginning of the ORF [codons]

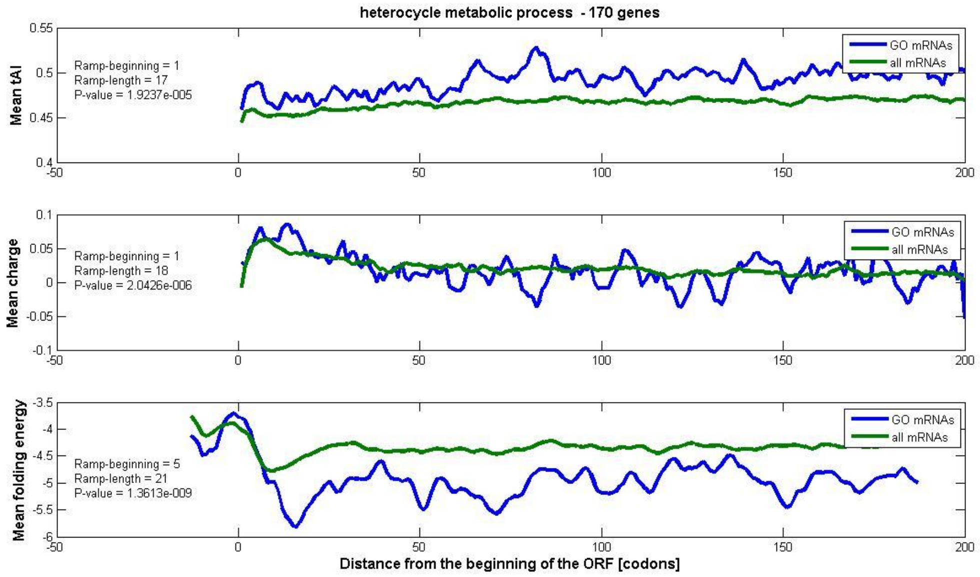
heterocycle metabolic process - 170 genes
Mean tAI
Ramp-beginning = 1
Ramp-length = 17
P-value = 1.9237e-005
GO mRNAs
all mRNAs
Mean charge
Ramp-beginning = 1
Ramp-length = 18
P-value = 2.0426e-006
Mean folding energy
Ramp-beginning = 5
Ramp-length = 21
P-value = 1.3613e-009
Distance from the beginning of the ORF [codons]

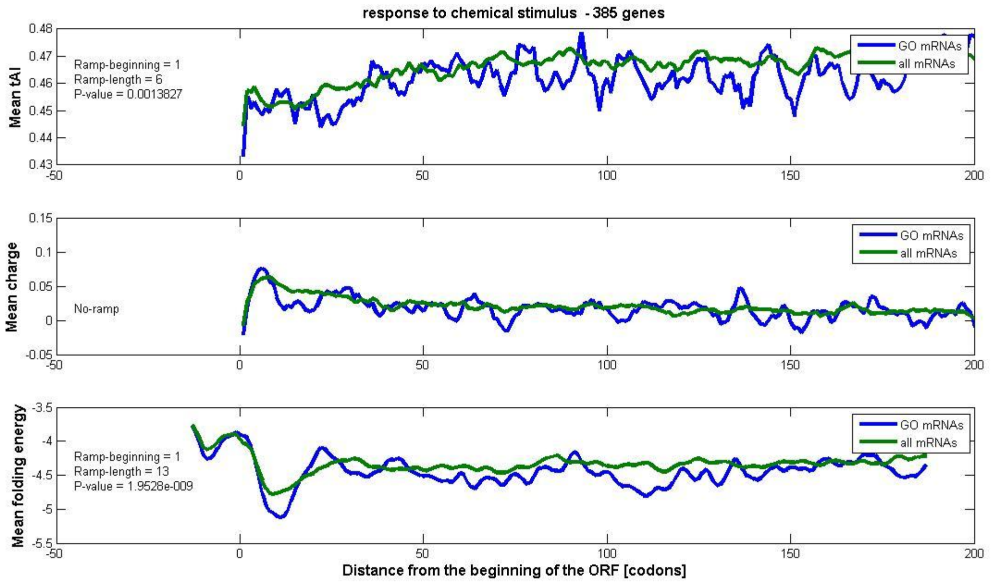
response to chemical stimulus - 385 genes
Mean tAI
Ramp-beginning = 1
Ramp-length = 6
P-value = 0.0013827
GO mRNAs
all mRNAs
Mean charge
No-ramp
Mean folding energy
Ramp-beginning = 1
Ramp-length = 13
P-value = 1.9528e-009
Distance from the beginning of the ORF [codons]

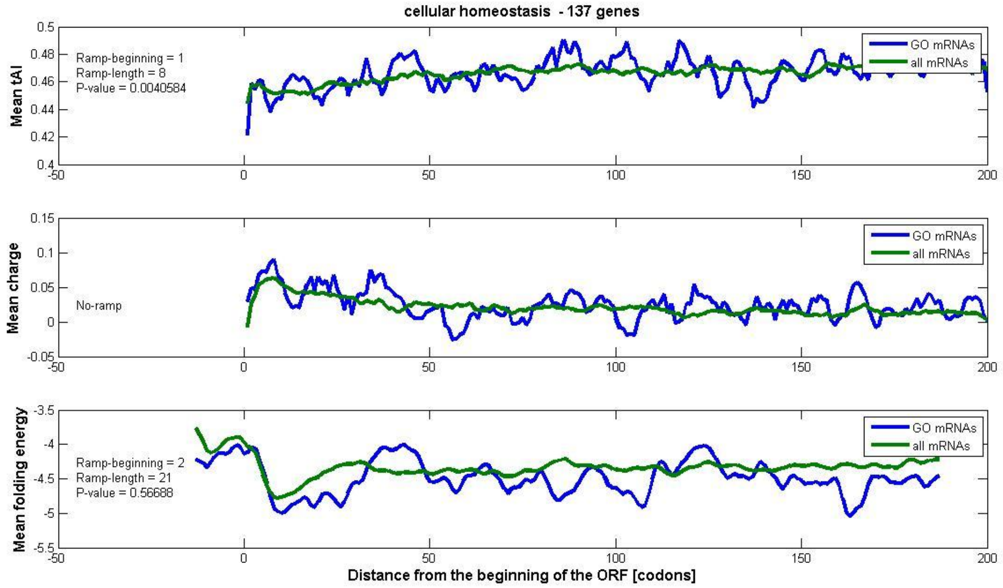
cellular homeostasis - 137 genes
Mean tAI
Ramp-beginning = 1
Ramp-length = 8
P-value = 0.0040584
GO mRNAs
all mRNAs
Mean charge
No-ramp
Mean folding energy
Ramp-beginning = 2
Ramp-length = 21
P-value = 0.56688
Distance from the beginning of the ORF [codons]
